# A Complex Recombination Pattern in the Genome of Allotetraploid *Brassica napus* as Revealed by a High-Density Genetic Map

**DOI:** 10.1371/journal.pone.0109910

**Published:** 2014-10-30

**Authors:** Guangqin Cai, Qingyong Yang, Bin Yi, Chuchuan Fan, David Edwards, Jacqueline Batley, Yongming Zhou

**Affiliations:** 1 National Key Laboratory of Crop Genetic Improvement, Huazhong Agricultural University, Wuhan, China; 2 Key Laboratory of Rapeseed Genetics and Breeding of Agriculture Ministry of China, Huazhong Agricultural University, Wuhan, China; 3 School of Agriculture and Food Sciences, University of Queensland, St Lucia, Queensland, Australia; Universidad Miguel Hernández de Elche, Spain

## Abstract

Polyploidy plays a crucial role in plant evolution. *Brassica napus* (2n = 38, AACC), the most important oil crop in the *Brassica* genus, is an allotetraploid that originated through natural doubling of chromosomes after the hybridization of its progenitor species, *B. rapa* (2n = 20, AA) and *B. oleracea* (2n = 18, CC). A better understanding of the evolutionary relationship between *B. napus* and *B. rapa*, *B. oleracea*, as well as Arabidopsis, which has a common ancestor with these three species, will provide valuable information about the generation and evolution of allopolyploidy. Based on a high-density genetic map with single nucleotide polymorphism (SNP) and simple sequence repeat (SSR) markers, we performed a comparative genomic analysis of *B. napus* with Arabidopsis and its progenitor species *B. rapa* and *B. oleracea*. Based on the collinear relationship of *B. rapa* and *B. oleracea* in the *B. napus* genetic map, the *B. napus* genome was found to consist of 70.1% of the skeleton components of the chromosomes of *B. rapa* and *B. oleracea*, with 17.7% of sequences derived from reciprocal translocation between homoeologous chromosomes between the A- and C-genome and 3.6% of sequences derived from reciprocal translocation between non-homologous chromosomes at both intra- and inter-genomic levels. The current study thus provides insights into the formation and evolution of the allotetraploid *B. napus* genome, which will allow for more accurate transfer of genomic information from *B. rapa*, *B. oleracea* and Arabidopsis to *B. napus*.

## Introduction

Polyploidy plays a crucial role in plant evolution [1], [2], [3]. Most flowering plants, including the majority of agricultural crops, are polyploid [2], [4]. Polyploidization can result in chromosomal rearrangements, changes in gene expression, reductions in chromosome numbers and evolution of the centromere [5], [6], [7], [8], [9]. However, the genetic, genomic and cytological factors determining nascent polyploid formation remain to be elucidated.

The *Brassica* genus consists of three elementary diploid species, *Brassica rapa* (AA, 2n = 2x = 20), *B. nigra* (BB, 2n = 2x = 16), and *B. oleracea* (CC, 2n = 2x = 18), and three amphidiploid species derived from the three diploids, *B. napus* (AACC, 2n = 4x = 38), *B. juncea* (AABB, 2n = 4x = 36), and *B. carinata* (BBCC, 2n = 2x = 34) [10]. *B. napus*, as the most important oil crop among the six species in the U-triangle, is estimated to have been generated 5,000 to 10,000 years ago by the natural hybridization of its two progenitor diploids, *B. rapa* and *B. oleracea* [11], [12]. *B. rapa* and *B. oleracea* were produced by extensive triploidization of their ancestral species at the genomic level [13]. The three species are believed to share a common ancestor with *Arabidopsis thaliana* (2n = 2x = 10) [9], [14]. *B. napus* is a relatively young species in terms of its evolutionary age and has a short history of artificial domestication (about 400–500 years) [12], [15]. Therefore, *B. napus* is an ideal model species to study the evolution of allopolyploidy [6], [16].

Compared to diploidy, the study of genomic structure in polyploidy is much more difficult and complex. With the vast amount of information gained from the genome sequences of an ever-increasing number of plant species, comparative genomics has proved to be a useful tool for understanding the complicated polyploid genome through the transfer of information and resources of related species [17], [18]. Therefore, it is especially important to conduct efficient comparative genomic studies of *Brassica* crops with Arabidopsis, which is a model plant for the whole plant kingdom [19], although complete genome sequences are currently still lacking for *B. napus*.

The conserved blocks of the Arabidopsis genome or ancestral karyotype (AK, 2n = 2x = 16) have been demonstrated for most of the *Brassica* species by comparative genomic analysis [9], [12]. Such comparative mapping with Arabidopsis normally uses markers with known sequences, such as restriction fragment length polymorphisms (RFLPs) [18], [20], [21], [22], [23], [24], [25], [26], intron polymorphisms (IPs) [27], EST-SSR markers [28], [29], EST-based SNP markers [30], [31] and gene-specific markers [32]. Recently, we developed a method for comparative mapping with Arabidopsis with SSR markers in *B. napus* [33]. Delourme et al. used a SNP Infinium array to construct a high-density integrated genetic map for comparative analysis with Arabidopsis [34].

Another important aspect of comparative genomic studies in Brassicaceae is the comparison within the agronomically important species of the *Brassica* genera, especially between the three elementary species and their respective aggregated species, for example, the collinear relationship between the A and C genomes of *B. napus* [18], [27], [35]. Wang et al. mapped the sequence-tagged markers from an integrated linkage map of *B. napus* onto the *B. rapa* A genome and identified discrepancies and inconsistent regions (maybe deletion, inversion and translocation) between the *B. napus* A genome and the *B. rapa* A genome [26]. Similarly, Chen et al. aligned 3,116 SNPs that were on a *B. napus* ultrahigh-density SNP bin map to the *B. rapa* reference genome and also identified inversions and insertion/deletion fragments [36]. Jiang et al. identified several genomic rearrangement events covering totally at least 5% of the A genome between *B. napus* and *B. rapa* [37]. Xiong et al. used cytological methods to study homoeolog pairing and chromosome rearrangements, aneuploidy, and homoeologous chromosome compensation in resynthesized *B. napus* [16]. The completion of the genome sequences of *B. rapa* [14] and *B. oleracea* [38], and the availability of 6K [39] and 60K [40], [41] SNP arrays for *B. napus* offer new opportunities for comparative genomic research on *B. napus* and its ancestral species, *B. rapa* and *B. oleracea*, as well as Arabidopsis using whole-genome high-throughput data.

In this study, a 6K SNP array (Illumina Infinium HD Assay) for *B. napus* [39] was applied to genotyping of a doubled haploid (DH) population and its parental lines [42]. A high-density genetic map with SNP and SSR markers was constructed and used for comparative genomic analysis with the *B. rapa*, *B. oleracea* and Arabidopsis genomes. The conserved blocks of Arabidopsis, as well as the homoeologous collinear fragments of *B. rapa* and *B. oleracea* were identified in the *B. napus* genetic map by screening the homoeologous loci of the markers in the genetic map. With this information, we were able to dissect the genetic composition in the A and C genomes of *B. napus* and uncover their evolutionary relationships with the ancestral species at the genomic level.

## Results and Discussion

### Construction of a high-density genetic map

SSR markers and a 6K SNP array containing 5,306 probes for *B. napus* [39] were used to genotype the HJ DH population and its parental lines. From 2,400 SSR primer pairs, 406 (16.9%) exhibited high quality polymorphism between the two parental lines, generating 473 SSR loci in the population. The loci were subsequently used for constructing the framework of the genetic map.

The call rate of the 6K SNP array was >0.7 for all 192 samples (190 DH lines and 2 parents), with an average of 0.86. There were 578 probes (10.9%) that were detected in less than 80% of samples and thus not included in further analysis. The remaining 4728 SNPs were used for cluster analysis using the GenomeStudio software. A total of 1850 SNPs from the array showed polymorphisms between the parental lines Hua 5 and J7005.

**Figure 1:**
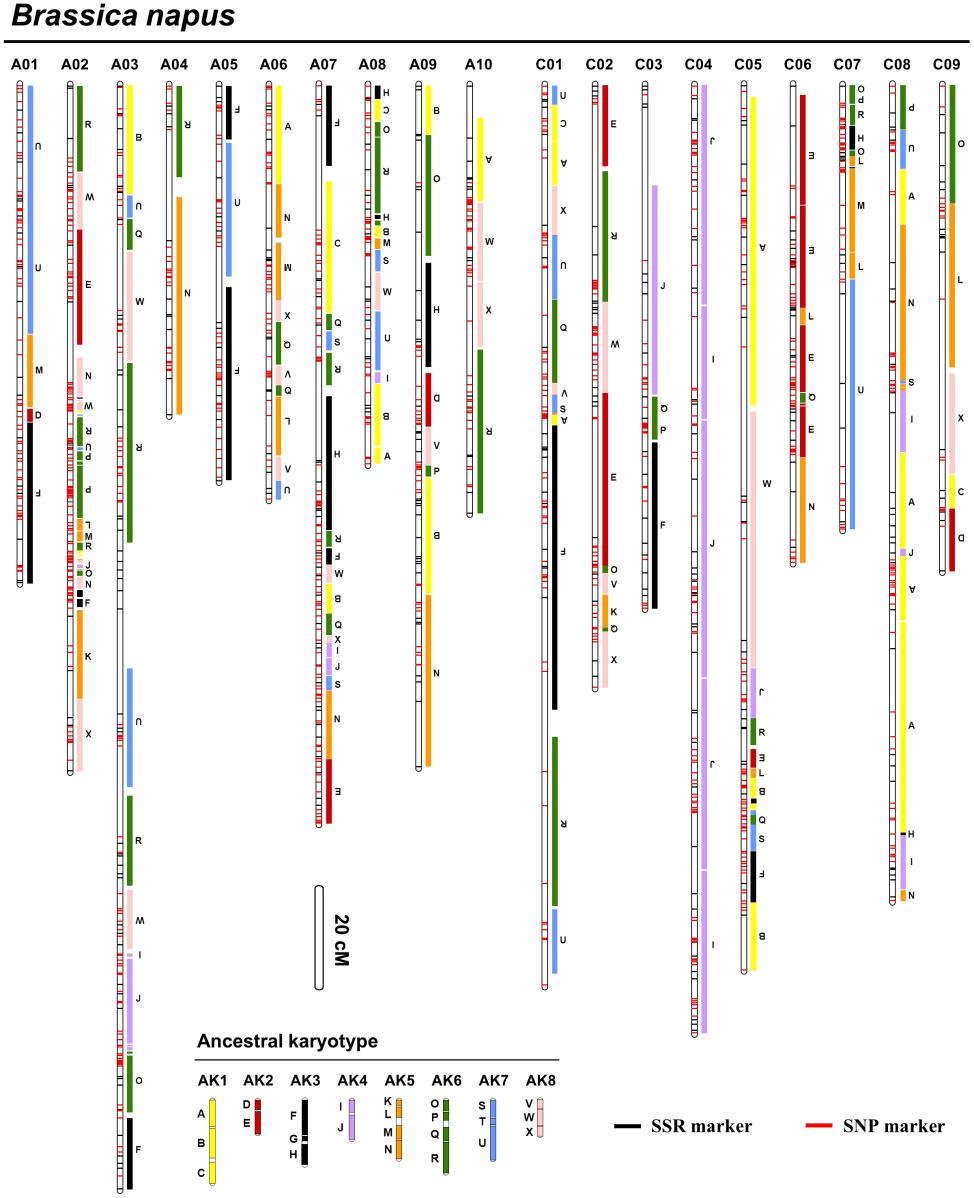
Conserved blocks of Brassicaceae Ancestral Karyotype on *Brassica napus* genetic map. For each linkage group (LG), the left vertical bar represents the LG with mapped markers (red dashes for single nucleotide polymorphisms (SNPs) and black for simple sequence repeat (SSR)). The conserved Arabidopsis blocks are listed on the right of each LG. The length of LG bars is proportional to their genetic distances. The conserved blocks are identified according to their positions in the Arabidopsis genome (see Materials and Methods) and depicted with colors based on the Ancestral Karyotype chromosome positions as described by Schranz et al. [9]. Inverted letters for respective conserved blocks indicate inversions in the LGs relative to Arabidopsis chromosomes. The length of each vertical bar for Ancestral karyotype chromosome is proportional to its physical length.

Linkage analysis was conducted with the 2,323 polymorphic loci (1850 SNPs and 473 SSR loci), and 2,115 markers (1667 SNPs and 448 SSR loci) were mapped onto 19 linkage groups (LGs) of *B. napus* (Table 1, Figure 1, Table S1). The total length of the genetic map was 2,477.4 cM, with an average distance of 1.27 cM between the markers (Table 1). The marker density (1.07 cM/marker) on the A genome of *B. napus* (designated as BnA-genome thereafter) was higher than that (1.49 cM/marker) on the C genome of *B. napus* (designated as BnC-genome thereafter).

**Table 1:** Comparative genomic analysis of *B. napus* with *B. rapa*, *B. oleracea* and Arabidopsis (the source of the data come from Table S1).

*B. napus*						Homoeologous collinear locus			Block	Island
LG	SSR marker	SNP marker	Total marker	Length (cM)	Average distance (cM/marker)	Arabidopsis	*B. rapa*	*B. oleracea*		
**A01**	19	110	129	105.1	0.81	74	104	0	5	0
**A02**	15	175	190	144.8	0.76	106	43	44	14	18
**A03**	47	149	196	233.2	1.19	99	95	45	11	4
**A04**	11	40	51	69.5	1.36	14	33	0	1	1
**A05**	16	58	74	83.5	1.13	35	44	4	3	0
**A06**	16	62	78	87.4	1.12	50	56	0	7	3
**A07**	33	154	187	155.9	0.83	89	112	14	12	5
**A08**	17	64	81	79.9	0.99	49	62	0	7	7
**A09**	36	61	97	143.9	1.48	40	67	0	7	2
**A10**	20	73	93	90.3	0.97	50	50	0	4	2
**Subtotal**	**230**	**946**	**1176**	**1193.5**	**1.07**	**606**	**666**	**107**	**71**	**42**
**C01**	18	99	117	190.1	1.62	50	9	37	7	6
**C02**	21	81	102	127.1	1.25	42	25	17	8	1
**C03**	22	37	59	110.4	1.87	20	0	33	3	1
**C04**	40	102	142	200.2	1.41	68	34	58	5	0
**C05**	27	113	140	186.9	1.34	77	50	27	13	3
**C06**	27	72	99	100.7	1.02	52	29	35	7	1
**C07**	15	97	112	93.7	0.84	41	0	73	8	2
**C08**	32	99	131	172.3	1.32	63	13	76	8	7
**C09**	16	21	37	102.5	2.77	14	0	20	2	3
**Subtotal**	**218**	**721**	**939**	**1283.9**	**1.49**	**427**	**160**	**376**	**61**	**24**
**Total**	**448**	**1667**	**2115**	**2477.4**	**1.27**	**1033**	**826**	**483**	**132**	**66**

### Comparative genomic analysis of *B. napus* with Arabidopsis

To identify the conservation and variation of the *B. napus* chromosomes compared with Arabidopsis or Ancestral Karyotype genome, the conserved blocks of Arabidopsis [9] in *B. napus* were identified through comparative genomic analysis of *B. napus* with Arabidopsis. For SSR markers, the locus sequences were inferred with the aid of homoeologous collinear loci in the *B. rapa/B. oleracea* genomes and then subjected to a BLASTn analysis with the Arabidopsis genome as described by Cai et al. [33]. For the SNP markers, the sequences of individual probes (300 bp on average) were directly used in the BLASTn analysis with Arabidopsis (E-value ≤ 1E-10). We identified 2,115 loci on the *B. napus* genetic map that could be matched to 1,930 loci in the Arabidopsis genome (Table S1). Conserved blocks and islands of Arabidopsis in *B. napus* were then detected using these loci. A conserved block on the map refers to a region harboring at least three molecular markers that includes at least two homoeologous loci in a 2 Mb fragment of one of the 24 defined blocks in the Arabidopsis genome for every 10 cM of the *B. napus* genetic map. If an Arabidopsis conserved block in *B. napus* had only two corresponding homoeologous loci, the block was classified as an island and named according to the block to which it belonged. In total, there were 132 conserved blocks and 66 islands that were deduced from 1033 Arabidopsis homoeologous loci (Figure 1, Table 1, 2 and S1). Together, these conserved blocks and islands covered 2,252 cM of the genetic linkage map of *B. napus*, accounting for approximately 90.9% of the total length (Figure 1, Table S1). The block T was not detected and the block G was only detected once (Table 2) in the map. The other 23 blocks were detected with an average frequency of 5.7 (Table 2).

**Table 2:** The copy number of the 24 identified conserved blocks and islands in the *B. napus* genetic map.

Conserved Block a	Copy	
	Block b	Island
**A**	8	4
**B**	6	4
**C**	4	2
**D**	2	1
**E**	10	0
**F**	11	1
**G**	1	0
**H**	6	2
**I**	5	3
**J**	7	4
**K**	1	1
**L**	7	1
**M**	3	4
**N**	6	4
**O**	4	5
**P**	5	2
**Q**	7	5
**R**	9	7
**S**	4	3
**T**	0	0
**U**	12	2
**V**	3	3
**W**	7	3
**X**	4	5
**Total**	**132**	**66**

a The conserved blocks are defined by Schranz et al. [9].

b The method for identification of the conserved block and island is described in the Materials and Methods section.

Interestingly, the LG C04 of *B. napus* (BnC04) was composed of only blocks I and J, indicating that it may entirely originate from the AK4 (ancestral karyotype) chromosome of the ancestral species (Figure 1, Table S1). The BnA04 and BnA05 were composed of the blocks and islands from two AK chromosome fragments, the BnC03 and BnC06 from three, the BnA01, BnA10, BnC02 and BnC07 from four, the BnA06 and BnC09 from five, the BnA03, BnA09, BnC01 and BnC08 from six, and the BnA08 was from seven. The BnA02, BnA07, and BnC05 were the most complex LGs/chromosomes and each of them contained all eight AK chromosomes (Table 1, Figure 1 and Table S1). These results suggest that *B. napus* chromosomes may vary greatly in terms of their origins of the ancestral genetic components. Understanding the mechanisms underlying the phenomenon will provide insights on how natural and artificial selection could shape the genetic variations in *B. napus* [43], [44].

### Dissection of the genomic composition of B. napus through comparative mapping with *B. rapa* and *B. oleracea*

*B. napus* is an allotetraploid species that is believed to have originated 5,000–10,000 years ago by natural doubling of chromosomes after the hybridization of its progenitor species, *B. rapa* and *B. oleracea* [10], [14]. With genetic maps constructed using RFLP and SSR markers, a large number of homoeologous collinear loci were identified in the BnA-genome and BnC-genome in addition to replacement, duplication, inversion, and translocation events [17], [27], [37], [45], [46],[47]. It was also proposed that there is a close collinear relationship of the BnA-genome and BnC-genome with the genomes of *B. rapa* and *B. oleracea* [9], [26], [36], [48], [49]. Although several studies have been conducted based on such an assumption [39], [50], [51], [52], [53], [54], the relationship between the BnA- and BnC- genomes and their relationships to their counterparts in the progenitor species are still elusive.

To conduct comparative genomic analyses with *B. rapa* and *B. oleracea*, the sequences of SNP markers on the map were aligned with *B. rapa* and *B. oleracea* genome sequences by means of the BLASTn tool. A locus with an E-value ≤ 1E-20 (best hit) in the *B. rapa* or *B. oleracea* genomes was considered to be a homoeologue to the query sequence of the SNP locus on the map. The loci in the *B. rapa* and *B. oleracea* genomes that were homoeologous to SSR markers were identified with the method described by Cai et al. [33]. In total 1,923 of the 2,115 markers (90.9%) on the map were matched to their homoeologous loci in the *B. rapa* and *B. oleracea* genomes (Table S1).

**Figure 2:**
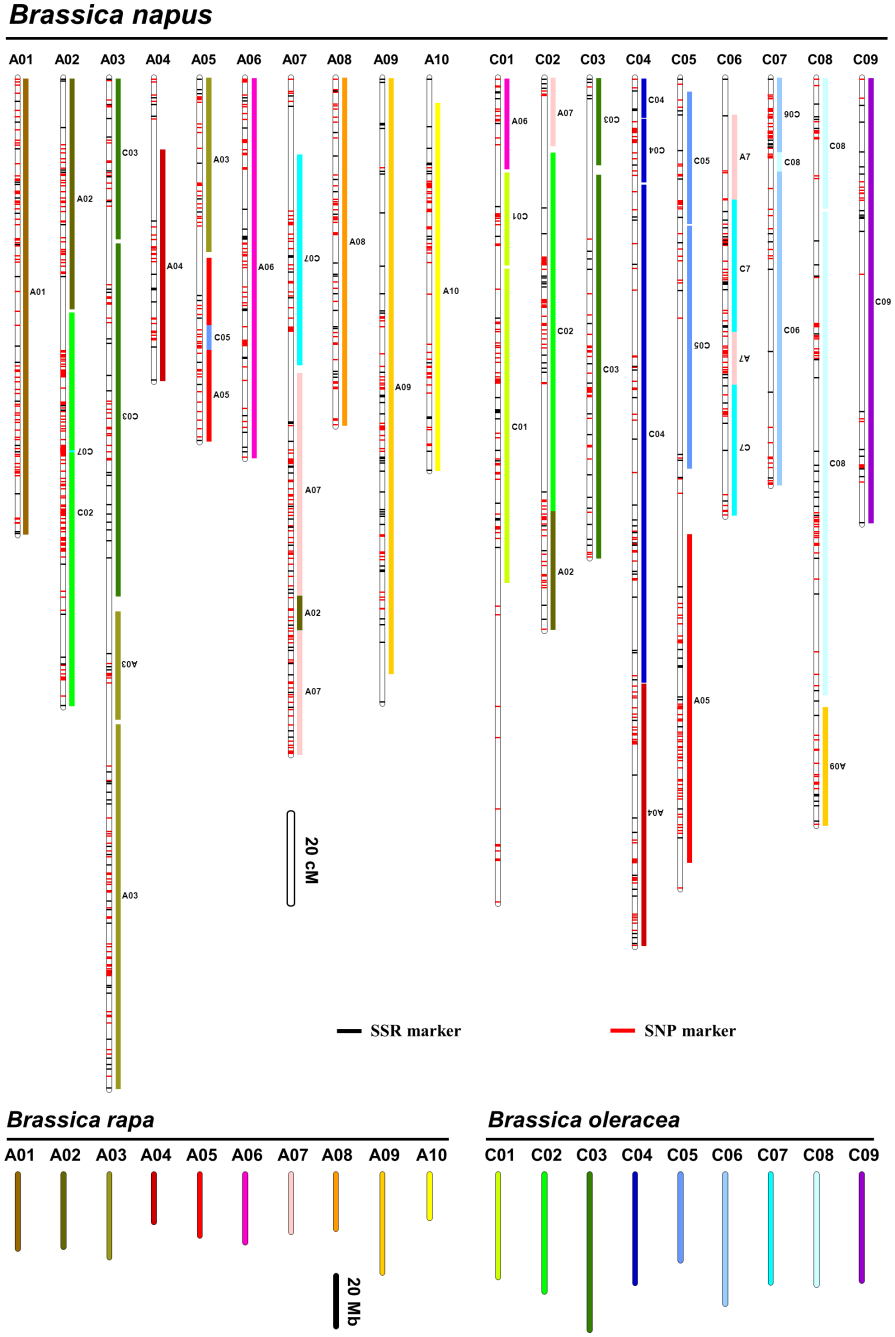
The relationship of the *Brassica napus* genetic map to the *B. rapa* and *B. oleracea* genomes. For each *B. napus* linkage group (LG), the left vertical bar represents the LG with mapped markers (red dashes for single nucleotide polymorphisms (SNPs) and black for simple sequence repeat (SSR)). The length of LG bars is proportional to their genetic distances. The homoeologous collinear fragments of *B. rapa* and *B. oleracea* identified in the *B. napus* genetic map are listed on the right, and colored based on the positions on *B. rapa* or *B. oleracea* chromosomes. Inverted letters for respective homoeologous collinear fragments indicate inversions in the LGs relative to *B. rapa* or *B. oleracea* chromosomes. The length of each vertical bar for *B. rapa* and *B. oleracea* chromosome is proportional to its physical length.

**Figure 3:**
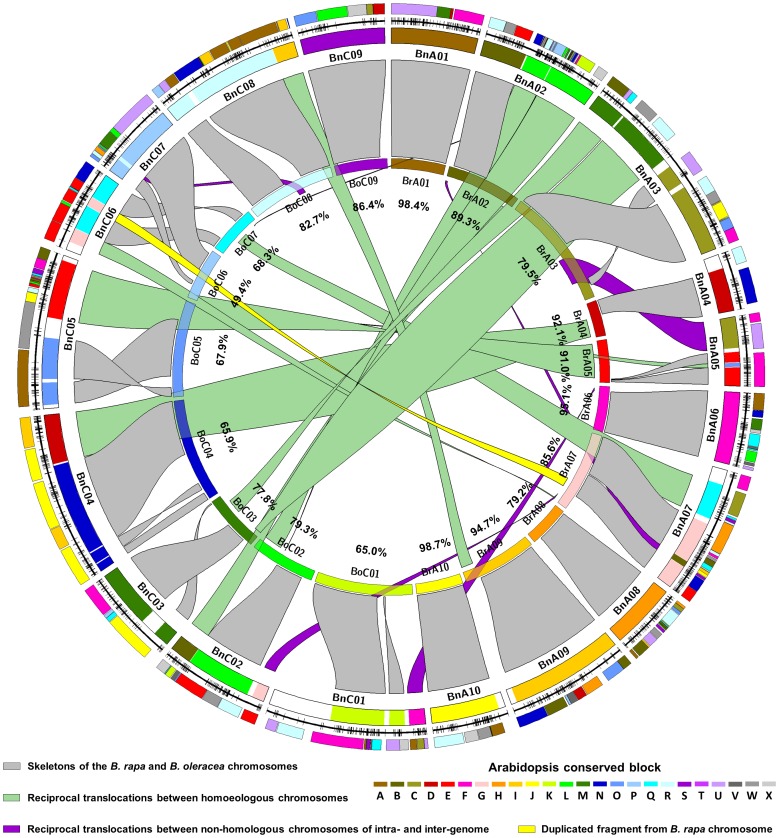
Evolutionary relationship between *Brassica napus* and its progenitor species *B. rapa* and *B. oleracea*. Schematic diagram of the *B. napus* genome as revealed by a genetic linkage map comprised of simple sequence repeat (SSR) and single nucleotide polymorphisms (SNP) markers and comparative analyses with the *B. rapa*, *B. oleracea* and Arabidopsis genomes. The colored blocks at the outermost circle represent the Arabidopsis conserved blocks in the *B. napus* genome identified with the genetic linkage groups of *B. napus*, which is represented in the second outer circle (all circles were orientated clockwise). The third circle (from the outermost one) represents the *B. napus* genome that is reconstructed with 46 homoeologous collinear fragments of *B. rapa* and *B. oleracea*. Each homoeologous collinear fragment of *B. napus* (the third circle) is the same color as the corresponding chromosome of *B. rapa* and *B. oleracea* in the inner circle. The ribbons between the third and inner circle depict the origins of the homoeologous collinear fragments from *B. rapa* and *B. oleracea*. The inverted homoeologous collinear fragments are indicated with twisted ribbons. The gray ribbons represent the skeletons from the *B. rapa* and *B. oleracea* genomes retained in *B. napus* genome; The green ribbons represent the reciprocal translocations between homoeologous chromosomes from the A and C genomes; The purple ribbons represent the reciprocal translocations between non-homologous chromosomes from the A and C genomes; The yellow ribbon represents the repeat fragment from *B. rapa*/*B. oleracea* chromosome. The numbers in the inner circle (under each *B. rapa* or *B. oleracea* chromosome) are the percentages of all homoeologous collinear fragments of *B. rapa* or *B. oleracea* retained in the *B. napus* genome relative to the physical length of the corresponding *B. rapa* (chromosome_v1.5) or *B. oleracea* (chromosome_v1.0) chromosome.

Based on the above analysis, we further identified homoeologous collinear fragments of *B. rapa* and *B. oleracea* in the *B. napus* genome using a similar method described by Parkin et al. [18]. Homoeologous collinear fragments of *B. rapa*/*B. oleracea* in *B. napus* were defined as DNA sequences that included at least four molecular markers in every 5 cM of the map, and simultaneously contained at least one homoeologous locus in a 2.5 Mb region of the corresponding *B. rapa*/*B. oleracea* genomes. Using this criterion, 22 homoeologous collinear fragments of *B. rapa* and 24 of *B. oleracea* were identified in *B. napus* (Figure 2, Table 3 and Table S1), which corresponded to 1,309 loci (Table 1, Table S1) and covered 2,237.1 cM (90.3%) of the whole *B. napus* genome (Table 3, Figure 2). Except for a 2.74 Mb fragment in chromosome A07 of *B. rapa* (BrA07) that had one duplicated copy located on BnC06 (yellow ribbon in Figure 3, Table 3), the rest of the 21 *B. rapa* fragments and 24 *B. oleracea* fragments appeared only once on each of the 19 LGs/chromosomes of *B. napus* (Table 3). These homoeologous collinear fragments of *B. rapa* and *B. oleracea* identified in *B. napus* genome accounted for 90.3% and 71.4% of the total length of the *B. rapa* and *B. oleracea* genomes, respectively, based on the known physical lengths of the two species (Figure 2, Table 3 and Table S1).

**Table 3:** The detailed information of 46 homoeologous collinear fragments of *B. rapa* and *B. oleracea* genomes identified in the *B. napus* genetic map (the source of the data come from Table S1).

*B. napus*		Homoeologous collinear fragment							
LG a	Length b	*B. rapa*				*B. oleracea*			
		Chr c	Span (bp)	% d	Length b	Chr c	Span (bp)	% d	Length b
**BnA01**	105.1	BrA01	284,970–28,424,481	98.4	105.1				
**BnA02**	144.8	BrA02	1,510,618–14,212,023	45.6	54.3	BoC02	25,050,175–41,757,926	37.9	90.1
						BoC07	36,597,360–37,488,062	2.2	0.5
**BnA03**	233.2	BrA03	501,255–16,229,444	49.6	84.1	BoC03	24,221–7,268,049	12.5	47.9
		BrA03	26,608,007–28,648,208	6.4	27.4	BoC03	32,383,728–47,645,315	26.4	38.4
**BnA04**	69.5	BrA04	276,812–7,920,797	40.3	57.5				
**BnA05**	83.5	BrA05	22,210,227–23,831,506	6.8	40.4	BoC05	32,350,587–32,701,848	1.1	5.4
		BrA03	18,834,614–26,272,710	23.5	38.7				
**BnA06**	87.4	BrA06	2,993,650–26,241,942	88.5	87.4				
**BnA07**	155.9	BrA07	277,845–11,178,806	48.3	50.2	BoC07	15,896,409–26,012,560	24.9	50.4
		BrA07	11,472,403–18,261,993	30.1	28.9				
		BrA02	14,309–810,600	2.9	8.0				
**BnA08**	79.9	BrA08	759,127–17,858,014	79.2	79.9				
**BnA09**	143.9	BrA09	936,877–29,820,627	77.8	137.0				
**BnA10**	90.3	BrA10	135,760–17,501,817	98.7	90.3				
**Subtotal**	**1193.5**				**889.2**				**232.6**
**BnC01**	190.1	BrA06	1,168,297–2,902,867	6.6	22.4	BoC01	10,671,090–11,302,996	1.6	34.6
						BoC01	13,821,490–38,372,637	63.3	57.2
**BnC02**	127.1	BrA02	15,879,592–27,246,329	40.8	26.6	BoC02	6,520,290–24,759,788	41.4	83.7
		BrA07	20,511,170–21,918,751	6.2	16.8				
**BnC03**	110.4					BoC03	8,598,996–29,818,084	36.7	86.9
						BoC03	55,727,445–56,984,210	2.2	23.5
**BnC04**	200.2	BrA04	8,431,313–18,248,518	51.8	60.2	BoC04	28,531–2,985,740	7.2	10.5
						BoC04	4,503,191–7,283,093	6.8	16.5
						BoC04	19,590,343–40,800,903	51.9	113.0
**BnC05**	186.9	BrA05	1,492,477–21,646,834	84.2	75.0	BoC05	283,542–3,205,578	8.9	37.3
						BoC05	7,007,360–26,021,073	57.9	34.0
**BnC06**	100.7	BrA07	14,970,124–17,705,637	12.1	13.0	BoC07	302,532–8,913,646	21.2	30.4
		BrA07	21,961,835–22,193,291	1.0	17.1	BoC07	27,513,541–35,677,613	20.1	29.7
**BnC07**	93.7					BoC06	12,451,594–15,878,853	7.1	17.0
						BoC06	24,097,329–44,570,444	42.3	72.7
						BoC08	23,087,748–26,244,524	7.6	4.0
**BnC08**	172.3	BrA09	30,571,654–36,860,095	16.9	27.3	BoC08	1,501,208–18,653,316	41.3	30.2
						BoC08	27,420,490–41,433,239	33.8	114.8
**BnC09**	102.5					BoC09	171,077–34,857,697	86.4	102.5
**Subtotal**	**1283.9**				**258.4**				**898.5**
**Total**	**2477.4**				**1147.5**				**1131.0**

a Linkage group.

b The unit of the length is cM.

c Chromosome.

d Percentage. The percentage refers that the proportion of the physical length of the homoeologous collinear fragment accounts for the whole physical length of the corresponding *B. rapa* (chromosome_v1.5) or *B. oleracea* (chromosome_v1.0) chromosome.

### Evolutionary relationship between the genomes of *B. napus* and its progenitor species *B. rapa* and *B. oleracea*

Further analysis was conducted to determine the relationships of the identified homoeologous collinear fragments of *B. napus* with the genomes of their two progenitor species (Table 3). Based on the comparison of the homoeologous collinear fragments with the genomes of *B. rapa* and *B. oleracea*, a total of 393.5 Mb of genomic components from 10 chromosomes of *B. rapa* and 9 of *B. oleracea* were identified to be conserved (Table 3 and Table S1), which formed a basic skeleton for each of 19 *B. napus* LGs/chromosomes (grey ribbons in Figure 3), corresponding to a total length of 1,736.9 cM of the *B. napus* map and 29 homoeologous collinear fragments (Table 3, grey ribbons in Figure 3, and Table S1). The remainder of the *B. napus* genome could result from chromosome re-arrangements (exchanges), including fragment duplication (yellow ribbon in Figure 3), inversions within a chromosome (twisted ribbons in Figure 3, Table S1) and translocations among different chromosomes (green and purple ribbons in Figure 3, Table S1).

Since *B. napus* is an allotetraploid containing the A-genome and C-genome from its progenitor species of *B. rapa* and *B. oleracea*, it would be expected that chromosome translocations could happen at both intra- and inter-genome levels. To distinguish the origins of translocation events in *B. napus* genome, we considered the reciprocal translocations occurred between the homoeologous chromosomes of the A-genome and C-genome [18] as homoeologous recombination. On the other hand, all other translocations between non-homologous chromosomes within the A-genome or C-genome (intra-genome) as well as between the A-genome and C-genome (inter-genome) were regarded as non-homologous recombination. Under such definitions, the reciprocal translocations between homoeologous chromosomes in the *B. napus* genome covered 438.3 cM of the genetic distance on the *B. napus* map, and could be linked to 11 corresponding homoeologous collinear fragments of *B. rapa* and *B. oleracea* that were equal to 97.5 Mb of the genomic components from *B. rapa* and *B. oleracea* (green ribbons in Figure 3, Table 3). On the other hand, the reciprocal translocations through non-homologous recombination of intra- and inter-genome covered 90.4 cM of the genetic distance on the *B. napus* map, and could be linked to 6 corresponding homoeologous collinear fragments of *B. rapa* and *B. oleracea* that were equal to 15.4 Mb of genomic components (purple ribbons in Figure 3, Table 3).

Based on the origins of the chromosome fragments, eight LGs/chromosomes of *B. napus* (BnA01, BnA04, BnA06, BnA08, BnA09, BnA10, BnC03 and BnC09) were found to contain only the skeletons from the corresponding chromosomes of *B. rapa* or *B. oleracea* (Figure 3, Table 3). These eight chromosomes of *B. napus* all contained intact genetic components of their progenitor species' chromosomes, except BnC03 on which one inversion and one translocation occurred (Figure 3). The rest of the 11 LGs/chromosomes of *B. napus* were composed of various chromosome fragments along with a skeleton of the progenitor species' chromosome (Figure 3, Figure 2 and Table 3). Interestingly, the DNA sequences in BrA01, BrA08 and BrA10, and BoC01, BoC04, BoC06 and BoC9 were only identified in their corresponding *B. napus* LGs/chromosomes of BnA01, BnA08 and BnA10, and BnC01, BnC04, BnC06 and BnC09 (Figure 3, Table 3).

Three types of the chromosome re-arrangements among the 11 LGs/chromosomes that consisted of a skeleton and various chromosome fragments from different origins were found. The first type could be defined as rearrangement from the reciprocal translocations between the homoeologous chromosomes of the A and C genomes, as well as intra-chromosome recombination (inversion and translocation), which were identified in BnA03, BnC04, BnC05, BnC06, and BnC08 (Figure 3 and Table 3). For example, BnC06 was composed of two different chromosomal fragments from BoC07 and BrA07. The chromosome contained a skeleton from BoC07, but the skeleton was divided into two reversed fragments with a BrA07 insertion that was duplicated once in a reversed orientation (Figure 2 and 3). BnC06 has previously been found to be aligned with BoC07, as well as BnC07 with BoC06 [27], [33]. Such reversed nomenclatures are based on the nomenclatures from Parkin et al. [17], [18]. It is possible now the obviously reversed nomenclatures of BnC06-BoC07 and BnC07-BoC06 could be corrected with the aids of sequencing and cytology.

With the genome sequences of *B. rapa* [14] and *B. oleracea* [38] publically available, we were able to examine the consistency of comparative mapping of *B. napus*-Arabidopsis (Figure 1) and *B. napus*-two progenitor species (Figure 2 and 3), To that end, we took BnC04 as an example. BnC04 was composed of a BoC04 skeleton and a large section of BrA04 at the distal end (Figure 2 and 3). The BoC04 skeleton on BnC04 consisted of two close but separated fragments at its upper end from the upper part of BoC04 and one large fragment at its middle part from the lower part of BoC04 (all circles were orientated clockwise in Figure 3). At the same time, there was a BrA04 fragment at the distal end of BnC04, which was originated from the distal end of BrA04 (Figure 3). Such an origin and distribution could explain why BnC04 only contained I and J blocks from AK4 in the comparison with Arabidopsis (Figure 1). The most recent study of *de novo* sequencing of *B. oleracea* and collinearity analysis of *B. rapa* and *B. oleracea* with Arabidopsis has showed that the upper and lower parts of the BoC04 (Supplementary Figure 22 of Liu et al. [38]) only contained I and J blocks, and the lower half of BrA04 was only composed of I and J blocks (Supplementary Figure 23 of Liu et al. [38]). Our results are consistent with these new sequencing data.

The second type of chromosome rearrangements in the *B. napus* genome was derived from reciprocal translocations between non-homologous chromosomes of the intra- and inter-genome, for example BnC01 and BnC07. These were formed by two reciprocal translocations between the non-homologous chromosomes in the A and C genomes and one intra-chromosomal inversion (Figure 2 and 3).

The third type of chromosome rearrangements included reciprocal translocations between the homoeologous chromosomes and between the non-homologous chromosomes, including BnA02, BnA05, BnA07 and BnC02. For example, BnA07 consisted of three different chromosomal sources, i.e. the skeleton of BrA07, a homologous fragment of BoC07, and a non-homologous fragment of BrA02 through intra-genomic translocation (Figure 2 and 3).

Overall, in the *B. napus* genome studied, there were 21 fragments from 10 *B. rapa* chromosomes and 24 fragments from 9 *B. oleracea* chromosomes. During the formation of the 19 *B. napus* chromosomes from the 45 progenitor fragments, 1 duplication (Figure 3, yellow ribbon), 17 inter-chromosomal reciprocal translocations (Figure 3, green and purple ribbons), 13 inversions (Figure 2, the fragments with inverted letters) and 3 intra-chromosomal translocations (Figure 3; BnA03, BnC03, and BnC05) were identified (Figure 2, Figure 3, Table 3 and Table S1). These results advanced our understanding of the formation and evolution of the *B. napus* genome, and allowed for a more effective utilization of *B. rapa* and *B. oleracea* genomic information in future *B. napus* genetic and genomic research, especially for fine mapping and the identification of genes for economically valuable traits in *B. napus*. However, the breakpoints of chromosomal rearrangements in *B. rapa* and *B. oleracea*, the evolution of the centromere, and the process of the formation of the new chromosomes await further in-depth study.

Based on the genetic distances, approximately 70.1% (1,736.9 cM) of the genetic components in the newly formed genome of *B. napus* was derived from the corresponding skeletons of the chromosomes of *B. rapa* and *B. oleracea*, 17.7% (438.3 cM) from homoeologous chromosome reciprocal translocations between the A and C genomes, and only 3.6% (90.4 cM) from non-homologous chromosome of intra- and inter- genomic translocations (Table 3 and Table S1). We found a higher number of genomic rearrangement events in the A genome (10%) than a previous report (5%) by Jiang et al. [37]. This is likely to be because of the lower density genetic map and absence of the C genome (*B. oleracea*) reference sequence at that time. The proportion of reciprocal translocations between homoeologous and non-homologous chromosomes in *B. napus* in this study may be overestimated. Firstly, the density of the HJ DH population genetic map was not saturated, which may result in an incomplete resolution on variations. Secondly, only two *B. napus* lines were analyzed. Thirdly, the current comparative analysis is only based on one diploid progenitor species sequences. Both *B. rapa* and *B. oleracea* are known to be two of the most genetically diverse diploid *Brassica* species. Fourth, *B. napus* in China has been modified by the frequent interspecific crossing with *B. rapa* in recent times. It remains to be determined how the mapping population in this study is similar or different compared with other germplasm of *B. napus* in terms of chromosome re-arrangements. Therefore, it would be difficult to establish if the variation observed in the *B. napus* genome was produced by the variation of the progenitor species, *B. rapa* and *B. oleracea*, or was derived from the modifications and selections during the demonstration of the *B. napus* genome.

Based on our analysis, approximately 90.3% and 71.4% of the genomic components of the *B. rapa* and *B. oleracea* genomes were conserved, respectively (Figure 3, Table 3). These differences in the genetic conservation of the A and C genomes may result from the partial loss of the C genome sequences during evolution. The stabilities of the A, B, and C genomes (or nucleolar dominance) in the *Brassica* genus were different, and the B genome was generally considered to be the most stable, whereas the C genome was considered to be the least stable (B>A>C) [55], [56], [57]. Therefore, a partial loss of the C genome sequences during the formation and demonstration of *B. napus* may not be surprising. Another possible explanation is that there are fewer differences in the C genome between the two parental lines used in this study. *B. rapa*, as one of the ancestors of *B. napus*, has been extensively planted in China with a great deal of variability. Chinese breeders introduced various components of the *B. rapa* genome for the improvement of various traits of *B. napus* [58], [59], resulting in a rich variation in the *B. napus* A genome. On the other hand, chromosome re-arrangement in *B. napus* has been reported not only in the accessions originated from China, but also in the materials originated from other regions such as European countries [17], [26], [27], [36], [37], [45], [46], [47], [59]. However, it is still not clear how the chromosome rearrangements among different *B. napus* accessions could vary. Further studies are needed to reveal how extensive the changes are in *B. napus* globally and to elucidate the evolutionary divergence between the A and C genomes of *B. napus*.

## Conclusions

With the high-resolution genetic map constructed with SSR and SNP markers, we were able to conduct a comparative genomic analysis of *B. napus* and its ancestral species, *B. rapa* and *B. oleracea*, as well as Arabidopsis. Compared to the other LGs/chromosomes of *B. napus*, LG C04 (BnC04) varied the least during the process of evolution; all of the genetic information of BnC04 came from chromosome 4 of the Ancestral Karyotype. Furthermore, the BnA02, BnA07, and BnC05 were the most complicated. According to the analysis of the homoeologous collinear fragments of *B. rapa* and *B. oleracea* identified in the *B. napus* genetic map, approximately 2/3 of the *B. napus* genome consists of the skeleton components of the chromosomes of *B. rapa* and *B. oleracea*, while approximately 1/5 consists of sequences reciprocal translocated between homoeologous chromosomes, and 1/20 consists of sequences reciprocal translocated between non-homologous chromosomes of the intra- and inter-genome. Our study advances the understanding of the complex pattern of the evolution of the *B. napus* genome, and allows for a more effective utilization of *B. rapa* and *B. oleracea* genomic information in *B. napus* genetic and genomic research.

## Materials and Methods

### Plant materials

The HJ DH population was produced from microspore culture of F1 buds of the cross between Huashuang 5 (Hua 5), a semi-winter type *B. napus* variety, and J7005, a winter-type *B. napus* pure line. The two parents were purified by microspore culture before hybridization. A total of 254 DH lines were obtained, and a random subset of 190 DH lines was sampled for the subsequent experiments. Detailed information about this population was described in Wu et al. [41]. The plant materials used in this study will be available to interested researchers according to PLoS ONE's requirements.

### Molecular markers and SNP array genotyping

Primer sequences for the SSR markers used for genetic map construction were described by Cai et al. [33] and the sequence information of all SSR markers is provided in Table S2.

The genotyping of SNPs was performed using 6K Illumina Infinium HD Assay SNP arrays of *B. napus* (Illumina Inc., San Diego, CA) developed by the University of Queensland. The high-quality DNA was extracted from young leaf tissues as described by Porebski et al. [60]. Each DNA sample was diluted to a final concentration of 50 ng/ µl using ddH_2_O. The SNP genotyping was carried out in accordance with the Illumina protocol (Infinium HD Assay Ultra Protocol Guide, http://www.illumina.com/).

All the SNP array data were analyzed using the Illumina GenomeStudio software (Illumina Inc., San Diego, CA), which were clustered and visualized for further analysis. Each SNP was re-checked manually to determine if any error was observed during the clustering analysis. The data processing is described by Raman et al. [39].

### Construction of genetic linkage map

Linkage analysis with all markers was performed using MAPMAKER/EXP 3.0 [61] and MSTmap [62] softwares. The MSTmap software can process more than 10,000 markers at one-time, while the MAPMAKER/EXP 3.0 can only process no more than 101 markers for one group. We firstly used MSTmap to process all the loci, and group the loci at 5.0 of log likelihood of the odds (LOD) score. Marker orders of each group were then calculated by finding the minimum spanning tree of a graph based on pairwise recombination frequencies [62]. At the same time, each group through MSTmap was calculated again through MAPMAKER/EXP 3.0 with a minimum LOD score of 11.0 and a maximum distance of 25 cM. The marker orders of each group obtained by MSTmap and MAPMAKER/EXP 3.0 were compared and the consistent regions of marker orders were retained. For inconsistent regions of marker orders, adjustments were made through re-calculating with more rigorous parameters (a minimum LOD score greater than 15, and a maximum distance less than 20 cM) by MAPMAKER/EXP 3.0. Genetic distances between markers were calculated using the Kosambi mapping function [63]. The nomenclature of LGs follows the rules proposed by The Multinational *Brassica* Genome Project (http://www.brassica.info/index.php). The linkage groups and corresponding graphs were drawn using the softwares MapDraw [64] and circos-0.62 (http://www.circos.ca/).

### Identification of Arabidopsis conserved blocks and homoeologous collinear fragments of *B. rapa* and *B. oleracea* genomes in the *B. napus* genetic map

The method of identifying the homoeologous locus in Arabidopsis with the SSR markers in the *B. napus* genetic map was as described in Cai et al. [33]. The SNP probe sequences were used as queries in searching for homoeologues using the BLASTn program [65] against TAIR10 (http://www.arabidopsis.org/) with an E-value threshold of 1E-10. The best-hit locus of BLASTn results was the homoeologous locus with Arabidopsis of each SNP locus in *B. napus* genetic map. A conserved block in the *B. napus* genome was defined as a region that, for every 10 cM of the *B. napus* genetic map with at least three molecular marker loci, at least two of the loci are homoeologous with a 2 Mb fragment of one of the 24 Arabidopsis conserved blocks [9]. If the region had only 2 homoeologous loci related to an Arabidopsis conserved block, then the block was classified as an island. Each island was named according to the block to which it belonged [66].

The method of identifying the loci in the *B. rapa* and *B. oleracea* genomes that are homoeologous and collinear with the SSR markers in the *B. napus* genetic map was described by Cai et al. [33]. The SNP probe sequences (the length of the sequence corresponding to each SNP probe was 300 bp on average) were used as queries in searching for homoeologues using the BLASTn program [65] against the *B. rapa* (http://brassicadb.org/brad/index.php, chromosome_v1.5) [14] and *B. oleracea* (http://www.ocri-genomics.org/bolbase/, chromosome_v1.0) [38] genomes with an E-value threshold of 1E-20. The best-hit locus of BLASTn results was the potential homoeologous collinear locus of each SNP locus in the *B. napus* genetic map. A homoeologous collinear fragment of the *B. rapa*/*B. oleracea* genome in *B. napus* was defined as a region that, in every 5 cM of the *B. napus* genetic map, has at least 4 molecular markers that simultaneously contained at least one homoeologous locus in the 2.5 Mb of the corresponding *B. rapa/B. oleracea* genomes.

## Supporting Information

Table S1 The detailed information of the genetic linkage map of the DH population constructed with SNP and SSR markers, the homoeologous loci and homoeologous collinear loci identified in *B. rapa*, *B. oleracea* and Arabidopsis, the homoeologous collinear fragments, and the conserved blocks and islands.
(PDF)

Table S2 The primer sequences of the SSR markers used in the HJ DH population genetic linkage map construction.
(PDF)
