# Supplementary material for: A Complex Recombination Pattern in the Genome of Allotetraploid Brassica napus as Revealed by a High-Density Genetic Map
Source: PLoS One. 2014 Oct 30;9(10):e109910. doi: 10.1371/journal.pone.0109910 (PMC4214627; doi:10.1371/journal.pone.0109910)
Supplement: Table S1 — The detailed information of the genetic linkage map of the DH population constructed with SNP and SSR markers, the homoeologous loci and homoeologous collinear loci identified in B. rapa, B. oleracea and Arabidopsis, the homoeologous collinear fragments, and the conserved blocks and islands. (PDF) [file pone.0109910.s001.pdf]

Table S1. Detailed information of the genetic linkage map of the HJ DH population, comparative genomic analysis with *B. rapa*, *B. oleracea* and Arabidopsis

B. napus			***B. rapa*/*B. oleracea***			***A. thaliana***		
**LG**	**Marker**	**Position**	**Homoeologous locus**	**Homoeologous collinear locus**		**Homoeologous locus**	**Conserved block/island**	
BnA01	bna1059	0	BrA01_284970_301 [a]	BrA01 [b]	284,970	AT2G29995		
BnA01	bna1060	0.7	BrA01_316954_302	BrA01	316,954	AT4G04800		
BnA01	bna5902	1.4	BrA01_344735_301	BrA01	344,735	AT1G22600		
BnA01	bna5487	3.3	BrA01_405988_295	BrA01	405,988	AT4G40040	AT4G40040	U [c]
BnA01	BEN334	5.2				AT4G36930	AT4G36930	U
BnA01	BGO001	5.2	BrA01_759441_243	BrA01	759,441	AT4G37250	AT4G37250	U
BnA01	bna4875	7.3	BrA01_806580_171	BrA01	806,580	AT3G44510		
BnA01	bna5488	7.3	BrA01_855235_301	BrA01	855,235	AT4G37080	AT4G37080	U
BnA01	bna1065	8.7	BrA01_922051_301	BrA01	922,051	AT4G36870	AT4G36870	U
BnA01	bna5005	8.7	BrA01_974796_295	BrA01	974,796	AT4G15093		
BnA01	bna1067	8.7	BrA01_975490_301	BrA01	975,490	AT4G12870		
BnA01	BEN9	10	BrA01_1040762_198	BrA01	1,040,762	AT4G36550	AT4G36550	U
BnA01	bna1070	11.8	BrA01_1103533_255	BrA01	1,103,533	AT4G36360	AT4G36360	U
BnA01	bna1073	14.1	BrA01_1291584_301	BrA01	1,291,584	AT4G35840	AT4G35840	U
BnA01	bna1074	14.7	BrA01_1322683_301	BrA01	1,322,683	AT4G35790	AT4G35790	U
BnA01	bna5432	14.7	BoC01_1878304_252			AT4G35320	AT4G35320	U
BnA01	bna1076	15.4	BrA01_1439859_301	BrA01	1,439,859	AT4G35310	AT4G35310	U
BnA01	bna1093	15.4	BrA01_1459501_301	BrA01	1,459,501	AT4G35270	AT4G35270	U
BnA01	NIAB_SSR106	16.1	BrA01_1466151_286	BrA01	1,466,151	AT4G35270	AT4G35270	U
BnA01	bna5906	19.6	BoC01_2488980_301			AT4G34138	AT4G34138	U
BnA01	bna1105	22.4	BrA01_1977120_301	BrA01	1,977,120	AT4G34060	AT4G34060	U
BnA01	bna1106	22.4	BrA01_2067347_301	BrA01	2,067,347	AT4G33740	AT4G33740	U

BnA01	bna1109	23.1	BrA01_2284682_297	BrA01	2,284,682	AT4G33080	AT4G33080	U
BnA01	bna1112	23.5	BrA01_2359318_213	BrA01	2,359,318	AT4G32910	AT4G32910	U
BnA01	BEN10	23.9	BrA01_2500870_113	BrA01	2,500,870	AT4G32680	AT4G32680	U
BnA01	BGO108	24.4	BrA01_2567859_160	BrA01	2,567,859	AT4G32590	AT4G32590	U
BnA01	bna1115	26	BrA01_2667541_301	BrA01	2,667,541	AT4G32320	AT4G32320	U
BnA01	bna1116	26	BrA01_2694173_301	BrA01	2,694,173	AT4G32280	AT4G32280	U
BnA01	bna1117	26.2	BrA01_2862804_302	BrA01	2,862,804	AT5G55550		
BnA01	bna1022	26.4	BoC04_18829093_301			AT2G14190		
BnA01	Ol10-D03A	27.5	BrA01_3282738_133	BrA01	3,282,738	AT1G71120		
BnA01	bna1121	28.2	BrA01_3304097_297	BrA01	3,304,097	AT2G24050		
BnA01	bna1123	28.2	BrA01_3640041_301	BrA01	3,640,041	AT4G29940	AT4G29940	U
BnA01	bna5499	28.6	BrA09_35709086_301			AT3G29572		
BnA01	bna0123	29	BrA01_3744574_303	BrA01	3,744,574	AT4G29730	AT4G29730	U
BnA01	bna5325	29	BoC01_4854933_314			AT4G29670	AT4G29670	U
BnA01	bna3175	30.1	BoC01_5700363_302			AT4G28670	AT4G28670	U
BnA01	bna3176	30.1	BoC01_5910378_284			AT2G07170		
BnA01	bna1125	30.1	BrA01_3969016_301	BrA01	3,969,016	AT4G14750		
BnA01	bna4825	30.1	BrA01_4027253_301	BrA01	4,027,253	AT5G38260		
BnA01	bna5033	30.1	BrA01_4077993_301	BrA01	4,077,993	AT4G29010	AT4G29010	U
BnA01	bna1129	30.1	BrA01_4246436_301	BrA01	4,246,436	AT4G28680	AT4G28680	U
BnA01	BEN2	31.1	BrA01_4276372_187	BrA01	4,276,372	AT4G28610	AT4G28610	U
BnA01	BnEMS1012	31.1	BrA01_4276389_211	BrA01	4,276,389	AT4G28610	AT4G28610	U
BnA01	bna1131	32.5	BrA01_4612731_282	BrA01	4,612,731	AT4G17890	AT4G17890	U
BnA01	bna5497	33.6	BrA01_5022756_301	BrA01	5,022,756	AT2G28053		
BnA01	bna1142	37	BrA01_5971798_301	BrA01	5,971,798	AT4G21050	AT4G21050	U
BnA01	bna1145	37.3	BrA01_6379546_301	BrA01	6,379,546	AT4G21895	AT4G21895	U

BnA01	bna1146	37.3	BrA01_6388281_309	BrA01	6,388,281	AT5G57800		
BnA01	bna1147	37.3	BrA01_6502314_301	BrA01	6,502,314	AT4G22120	AT4G22120	U
BnA01	bna5365	37.8	BrA01_6681885_301	BrA01	6,681,885	AT4G22540	AT4G22540	U
BnA01	BGR65	38.3	BrA01_6684759_104	BrA01	6,684,759	AT4G22540	AT4G22540	U
BnA01	bna1150	38.6	BrA01_6898141_301	BrA01	6,898,141	AT3G42565		
BnA01	bna4633	40.8	BrA01_7399677_301	BrA01	7,399,677	AT3G45200		
BnA01	bna1154	40.8	BrA01_7473115_301	BrA01	7,473,115	AT4G23750	AT4G23750	U
BnA01	bna1155	40.8	BrA01_7486801_301	BrA01	7,486,801	AT1G68750		
BnA01	bna1156	40.8	BrA01_7530818_301	BrA01	7,530,818	AT5G43530		
BnA01	bna5269	40.8	BrA01_7554524_301	BrA01	7,554,524	AT4G23950	AT4G23950	U
BnA01	bna1157	40.8	BrA01_7721347_301	BrA01	7,721,347	AT4G24220	AT4G24220	U
BnA01	bna1159	41.8	BrA01_7855239_303	BrA01	7,855,239	AT4G24490	AT4G24490	U
BnA01	bna1160	41.8	BrA01_7991655_302	BrA01	7,991,655	AT4G14030		
BnA01	bna1168	42.4	BrA01_8514979_301	BrA01	8,514,979	AT3G59410		
BnA01	BnEMS57	45.6	BrA01_8639083_251	BrA01	8,639,083	AT4G26080	AT4G26080	U
BnA01	bna1181	49.1	BrA01_9203246_301	BrA01	9,203,246	AT4G26190	AT4G26190	U
BnA01	bna1880	53.7	BrA01_11302933_301	BrA01	11,302,933	AT2G17370		
BnA01	bna1202	56.9	BrA01_14804405_301	BrA01	14,804,405	AT3G48660	AT3G48660	M
BnA01	bna5331	56.9	BrA01_14915561_301	BrA01	14,915,561	AT3G48810	AT3G48810	M
BnA01	Na12-C06	61.7						
BnA01	BGR6	65.4	BrA01_14939760_138	BrA01	14,939,760	AT3G48900	AT3G48900	M
BnA01	bna1205	66.3	BrA01_14960083_301	BrA01	14,960,083	AT3G48930	AT3G48930	M
BnA01	bna4847	67.2	BrA01_16129488_301	BrA01	16,129,488	AT2G03720		
BnA01	bna5087	67.2	BrA01_16228867_278	BrA01	16,228,867	AT1G58520	AT1G58520	D
BnA01	bna1952	67.2	BrA01_16253613_301	BrA01	16,253,613	AT1G59520	AT1G59520	D
BnA01	bna1951	67.5	BrA01_16260435_301	BrA01	16,260,435	ATCG00430		

BnA01	bna1949	68.9	BrA01_16433096_275	BrA01	16,433,096	AT1G59820	AT1G59820	D
BnA01	bna1948	68.9	BrA01_16472423_301	BrA01	16,472,423	AT5G17690		
BnA01	bna1946	69.9	BrA01_16530023_301	BrA01	16,530,023	AT5G39660		
BnA01	bna5381	69.9	BrA01_16945994_301	BrA01	16,945,994	AT1G53360		
BnA01	bna5604	69.9	BrA01_17112397_301	BrA01	17,112,397	AT1G60830	AT1G60830	D
BnA01	bna1947	69.9	BrScaffold004113_153_300			AT1G59910	AT1G59910	D
BnA01	bna1938	69.9	BrA01_17296297_299	BrA01	17,296,297	AT3G21870		
BnA01	bna1216	71.8	BrA01_18097124_301	BrA01	18,097,124	AT3G56275		
BnA01	bna5508	72.6	BoC01_25604906_301			AT3G23940	AT3G23940	F
BnA01	bna1215	72.6	BrA01_18102853_269	BrA01	18,102,853	AT5G49840		
BnA01	BGR84	74.5	BrA01_19723537_119	BrA01	19,723,537	AT3G23130	AT3G23130	F
BnA01	bna4621	75.2				AT1G45020		
BnA01	bna1234	75.6	BrA01_20436893_250	BrA01	20,436,893	AT5G29000		
BnA01	bna5510	75.6	BoC01_13892489_301					
BnA01	bna1240	77.2	BrA01_21533280_301	BrA01	21,533,280	AT3G19580	AT3G19580	F
BnA01	bna1227	77.8	BrA01_21715312_301	BrA01	21,715,312	AT3G19170	AT3G19170	F
BnA01	bna1226	77.8	BrA01_21726916_301	BrA01	21,726,916	AT3G19130	AT3G19130	F
BnA01	bna5240	78.8	BrA01_22141137_301	BrA01	22,141,137	AT3G18390	AT3G18390	F
BnA01	bna1242	79.4	BrA01_22680380_255	BrA01	22,680,380	AT5G25370		
BnA01	bna5255	81.2	BrA03_2569659_301					
BnA01	bna2684	82.8	BrA01_23024808_206	BrA01	23,024,808	AT5G63620		
BnA01	bna5050	83.3	BrA01_23024808_206	BrA01	23,024,808	AT5G63620		
BnA01	bna1244	83.3	BrA01_23159955_209	BrA01	23,159,955	AT1G64770		
BnA01	bna5184	83.3	BrA01_23169456_302	BrA01	23,169,456	ATCG00050		
BnA01	bna1246	83.3	BrA01_23390600_301	BrA01	23,390,600	AT5G29231		
BnA01	bna1250	83.3	BrA01_23571784_301	BrA01	23,571,784	AT2G13116		

BnA01	BEN68	84.6	BrA01_23659029_106	BrA01	23,659,029			
BnA01	bna3349	85.4	BoC01_32601699_301			AT3G16650	AT3G16650	F
BnA01	bna5513	85.4	BrA01_23718576_301	BrA01	23,718,576	AT3G15980	AT3G15980	F
BnA01	BnGMS87	86.2	BrA01_23784674_221	BrA01	23,784,674	AT5G21100		
BnA01	bna3358	86.7	BoC01_33124503_303			AT3G15930	AT3G15930	F
BnA01	bna1260	87.2	BrA01_24487702_301	BrA01	24,487,702			
BnA01	bna1269	88.8	BoC01_35362787_314			AT3G13530	AT3G13530	F
BnA01	bna4831	88.8	BrA01_24694541_172	BrA01	24,694,541	AT3G05460		
BnA01	bna0776	88.8	BoC01_35286677_301			AT3G13590	AT3G13590	F
BnA01	bna1277	89.8	BrA01_26384627_301	BrA01	26,384,627	AT3G62790		
BnA01	bna5656	89.8	BrScaffold000164_241494_301			AT3G13410	AT3G13410	F
BnA01	bna0344	89.8	BrA01_26442983_301	BrA01	26,442,983	AT3G01185	AT3G01185	F
BnA01	bna1280	90.3	BrA01_26605007_302	BrA01	26,605,007	AT2G35630		
BnA01	bna1281	90.8	BrA01_26622397_301	BrA01	26,622,397	AT3G11910	AT3G11910	F
BnA01	bna1293	90.9	BrA01_27159148_301	BrA01	27,159,148	AT3G11000	AT3G11000	F
BnA01	bna1295	91	BrA01_27389556_298	BrA01	27,389,556	AT4G04690		
BnA01	bna3367	91.1	BoC01_36821459_254			AT3G11000	AT3G11000	F
BnA01	bna1287	91.7	BrA01_27855099_301	BrA01	27,855,099	AT3G07810	AT3G07810	F
BnA01	BEN32A	95.9	BrA01_27859602_165	BrA01	27,859,602	AT3G07790		
BnA01	bna0893	101.5	BoScaffold000425_84667_264			AT3G02710	AT3G02710	F
BnA01	bna0962	101.6	BoScaffold000425_184083_301			AT3G02900	AT3G02900	F
BnA01	bna0963	101.7	BoScaffold000425_184083_301			AT3G02900	AT3G02900	F
BnA01	bna5974	101.8	BoScaffold000438_61616_301			AT1G53163		
BnA01	bna0300	102.5	BrScaffold000191_156554_301			AT1G61420		
BnA01	bna1291	102.6	BrA01_27926342_301	BrA01	27,926,342	AT1G36105		
BnA01	bna2273	102.7	BrA01_28153164_301	BrA01	28,153,164	AT3G06490	AT3G06490	F

BnA01	bna2272	102.7	BrA01_28168240_301	BrA01	28,168,240	AT3G06470	AT3G06470	F
BnA01	Na12-H02	104.6	BrA01_28229113_223	BrA01	28,229,113	AT3G06270	AT3G06270	F
BnA01	BRAS074B	105.1	BrA01_28424481_120	BrA01	28,424,481	AT4G08960		
BnA02	BoGMS795	0						
BnA02	Ol11-H09A	0.5	BrA02_1510618_161	BrA02	1,510,618	AT5G01400	AT5G01400	R
BnA02	sR12095	0.5	BrA02_1598382_324	BrA02	1,598,382	AT5G02030	AT5G02030	R
BnA02	BEN406	11.2				AT5G19510	AT5G19510	R
BnA02	bna5528	15.2	BrA02_4103904_301	BrA02	4,103,904	AT5G16960	AT5G16960	R
BnA02	bna4819	16.1	BoScaffold000328_209205_301			AT5G14110		
BnA02	bna1349	17	BrA02_4120287_301	BrA02	4,120,287	AT5G17050	AT5G17050	R
BnA02	bna3673	17	BoC08_4067834_301			AT3G28400		
BnA02	bna1351	17	BrA02_4180732_301	BrA02	4,180,732	AT5G17320	AT5G17320	R
BnA02	bna5531	18.4	BrA02_5991316_302	BrA02	5,991,316	AT5G59900	AT5G59900	W
BnA02	bna1971	18.4	BrA02_6252346_301	BrA02	6,252,346	AT5G59070	AT5G59070	W
BnA02	bna1367	19.2	BrA02_6466142_299	BrA02	6,466,142	AT5G58300	AT5G58300	W
BnA02	bna1371	20.6	BrA02_6766467_301	BrA02	6,766,467	AT4G23103		
BnA02	sN3761B	21	BrA02_6754912_171	BrA02	6,754,912	AT5G57510	AT5G57510	W
BnA02	sR6293	21.5	BrA02_7173113_124	BrA02	7,173,113	AT5G02880		
BnA02	bna1374	21.9	BrA02_7236554_297	BrA02	7,236,554	AT1G05190		
BnA02	bna5534	22.1	BrA02_7190128_301	BrA02	7,190,128	AT5G56530	AT5G56530	W
BnA02	bna4833	22.2	BrA02_7105284_302	BrA02	7,105,284	AT4G08910		
BnA02	bna5910	22.3	BrA02_7275448_301	BrA02	7,275,448	AT5G56320	AT5G56320	W
BnA02	bna3412	23.4	BrA02_7637663_301	BrA02	7,637,663	AT5G54830	AT5G54830	W
BnA02	bna1387	23.9	BrA02_8179342_301			AT3G17160		
BnA02	bna1388	23.9	BrA02_8249688_301			AT5G48000		
BnA02	bna5072	24.6	BrA02_7750536_301	BrA02	7,750,536	AT5G54510	AT5G54510	W

BnA02	bna1382	24.6	BrA02_7878079_301	BrA02	7,878,079	AT5G40020		
BnA02	bna5119	24.6	BrA02_7881877_301	BrA02	7,881,877	AT2G23060		
BnA02	bna1379	26	BrA02_7756158_301	BrA02	7,756,158	AT2G16670		
BnA02	bna1380	26	BrA02_7788690_301	BrA02	7,788,690	AT5G54490	AT5G54490	W
BnA02	bna5538	27.5	BrA02_8447213_285	BrA02	8,447,213	AT5G53060	AT5G53060	W
BnA02	bna1391	27.5	BrScaffold000318_1244_301			AT1G52920		
BnA02	bna1390	27.5	BoC06_25238316_301			AT1G72000		
BnA02	bna1393	27.5	BrA02_8625702_301	BrA02	8,625,702	AT2G15400		
BnA02	bna0125	27.5	BrA02_8738403_301	BrA02	8,738,403	AT5G52300	AT5G52300	W
BnA02	bna3513	28.2	BoScaffold000001_P2_469158_301			AT2G18721		
BnA02	bna5442	28.3	BrA02_8884562_301	BrA02	8,884,562	AT2G29940		
BnA02	bna1398	28.4	BrA02_8949807_301	BrA02	8,949,807	AT5G51660	AT5G51660	W
BnA02	bna1401	28.5	BrA02_9281581_303	BrA02	9,281,581	AT1G43886		
BnA02	bna0375	29.4	BrA01_3229012_301			AT4G05073		
BnA02	bna1698	30.7	BrA02_9421963_300	BrA02	9,421,963	AT1G65040	AT1G65040	E
BnA02	bna4638	30.7	BrA02_9645918_301	BrA02	9,645,918	AT5G54980		
BnA02	bna1441	32.8	BrA02_10325878_301	BrA02	10,325,878			
BnA02	bna2864	33.1	BoC01_19115033_293			AT3G30235		
BnA02	BEN281B	33.6	BrA02_10444068_202	BrA02	10,444,068	AT1G67730	AT1G67730	E
BnA02	bna0362	36.7	BrA02_10956748_301	BrA02	10,956,748	AT5G18450		
BnA02	bna1447	37.6	BrA02_11015035_301	BrA02	11,015,035	AT3G09390		
BnA02	bna1445	39.4	BrA02_11183543_301	BrA02	11,183,543	AT1G68920	AT1G68920	E
BnA02	bna1406	39.8	BrA02_11666536_303	BrA02	11,666,536	AT1G20240		
BnA02	bna1404	40.2	BrA02_11454601_301	BrA02	11,454,601	AT1G28760		
BnA02	bna1407	40.6	BrA02_11750590_301	BrA02	11,750,590	AT1G70260	AT1G70260	E
BnA02	bna5073	41.8	BrA02_12314392_301	BrA02	12,314,392	AT5G39680		

BnA02	bna5852	42.3	BrA02_12589154_301	BrA02	12,589,154	AT3G20320		
BnA02	bna1418	43.2	BrA02_12688756_301	BrA02	12,688,756	AT1G72460	AT1G72460	E
BnA02	bna1420	43.2	BrA02_12756742_296	BrA02	12,756,742	AT1G72530	AT1G72530	E
BnA02	bna0786	44.6	BoC02_18798720_286			AT5G48810		
BnA02	bna1424	44.9	BrA02_13556242_301	BrA02	13,556,242	AT1G15460		
BnA02	BGR87	45.7	BrA02_14212023_120	BrA02	14,212,023	AT1G76580	AT1G76580	E
BnA02	bna3960	62.8	BoC02_28444056_301			AT3G63070	AT3G63070	N
BnA02	bna3438	63.1	BoC02_25050175_278	BoC02	25,050,175	AT2G37220		
BnA02	bna3456	63.1	BoC02_25719601_239	BoC02	25,719,601	AT3G55610	AT3G55610	N
BnA02	bna4790	63.1	BoC02_27031205_301	BoC02	27,031,205	AT3G43160		
BnA02	bna3269	63.1	BoScaffold000335_230394_301			AT3G25020		
BnA02	bna3272	63.1	BoScaffold000335_100012_301			AT2G31040		
BnA02	bna3496	63.6	BoC02_27104488_302	BoC02	27,104,488	AT2G18850	AT2G18850	H
BnA02	bna4130	63.6	BoC08_9033619_300			AT2G14370	AT2G14370	H
BnA02	bna3281	63.8	BoC07_37417143_299			AT2G11410	AT2G11410	H
BnA02	bna3280	63.9	BoC07_37454319_301			AT5G65920	AT5G65920	X
BnA02	bna3476	64.2	BoC02_26310873_301	BoC02	26,310,873	AT5G63090	AT5G63090	X
BnA02	bna5838	64.2	BoC07_36658059_301			AT5G51360	AT5G51360	W
BnA02	bna3965	64.7	BoC02_28292620_301	BoC02	28,292,620	AT5G49870	AT5G49870	W
BnA02	bna3541	65.2	BoC02_31762247_301			AT5G49790	AT5G49790	W
BnA02	bna3487	65.9	BoC02_26757922_301	BoC02	26,757,922	AT5G45230	AT5G45230	W
BnA02	bna3284	66.4	BoC09_27155188_301			AT1G34570	AT1G34570	B
BnA02	bna3296	66.4	BoC07_36508723_301			AT1G26310	AT1G26310	B
BnA02	bna3962	67.2	BoC02_28386263_301	BoC02	28,386,263			
BnA02	bna3964	67.2	BoC02_28311847_301	BoC02	28,311,847	AT1G12680	AT1G12680	A
BnA02	bna3470	67.6	BoC03_41885114_301			AT1G07940	AT1G07940	A

BnA02	bna3959	67.9	BoC02_28482686_301	BoC02	28,482,686	AT5G35413	AT5G35413	S
BnA02	bna5837	68.2	BoC07_37452969_301			AT5G35205	AT5G35205	S
BnA02	bna1021	68.5	BoC02_28822177_301	BoC02	28,822,177	AT5G28145		
BnA02	bna3298	68.8	BoC06_1908498_229			AT5G10480	AT5G10480	R
BnA02	bna5846	71.4	BoC02_29084192_301			AT5G09463	AT5G09463	R
BnA02	bna3468	74.6	BoC02_26067961_300	BoC02	26,067,961	AT5G03280	AT5G03280	R
BnA02	bna3277	74.6	BoC07_37555131_301			AT5G02390	AT5G02390	R
BnA02	bna3240	74.9	BoC02_29075223_301			AT4G38760	AT4G38760	U
BnA02	bna3457	75.2	BoC02_25741664_301	BoC02	25,741,664	AT4G29420	AT4G29420	U
BnA02	bna3290	75.2	BoC07_36672987_301			AT4G26190	AT4G26190	U
BnA02	sR10417	75.5	BrA02_17125071_227			AT4G23900	AT4G23900	U
BnA02	bna3467	76	BoC02_26044159_301	BoC02	26,044,159	AT4G10300	AT4G10300	P
BnA02	BGR52	76.5	BrA02_17526120_144			AT4G10140	AT4G10140	P
BnA02	bna3289	76.8	BoC07_36714679_301			AT4G07664	AT4G07664	P
BnA02	bna3263	76.8	BrA05_8520929_145			AT4G06630	AT4G06630	P
BnA02	bna3530	77.9	BrA10_62140_301			AT4G06626	AT4G06626	P
BnA02	bna3255	78.6	BoC02_23357144_301			AT4G04620	AT4G04620	O
BnA02	bna0612	79.3	BoScaffold000233_755201_170			AT4G03390	AT4G03390	O
BnA02	bna3956	80.2	BoC02_28779755_301	BoC02	28,779,755	AT5G44020	AT5G44020	V
BnA02	bna3955	80.2	BoC02_28805541_301	BoC02	28,805,541	AT5G44070	AT5G44070	V
BnA02	bna3548	80.8	BoC02_31576602_301			AT4G32330	AT4G32330	P
BnA02	bna3474	81.4	BoC02_26265513_301	BoC02	26,265,513	AT2G01140		
BnA02	bna4720	81.4	BoC02_21299338_301			AT4G23800	AT4G23800	P
BnA02	bna3257	81.4	BoC02_23381964_301	BoC02	23,381,964	AT3G49130		
BnA02	bna3491	83.3	BoC02_26921898_264	BoC02	26,921,898	AT4G23070	AT4G23070	P
BnA02	bna3291	84.6	BoC07_36664505_301			AT2G39130		

BnA02	bna3493	84.7	BoC02_27046229_278	BoC02	27,046,229	AT3G33004		
BnA02	bna3256	84.9	BoC03_32323085_302			AT4G22130	AT4G22130	P
BnA02	bna3453	85.2	BoC07_25305432_301			AT3G29180		
BnA02	bna4704	85.2	BoScaffold000419_82799_291			AT1G36670		
BnA02	bna3492	85.2	BoC08_13180288_301			AT5G46240		
BnA02	bna3484	85.2	BoC02_26666221_258	BoC02	26,666,221			
BnA02	bna3452	85.3	BoC02_25442729_300	BoC02	25,442,729	AT4G21680	AT4G21680	P
BnA02	bna3443	85.4	BoC02_25146228_302	BoC02	25,146,228	AT5G56270		
BnA02	bna3449	85.5	BoC02_25222313_294	BoC02	25,222,313	AT1G71970		
BnA02	bna5858	85.6	BrA03_21378186_130			AT5G01110		
BnA02	bna3279	85.7	BoC07_37488062_301	BoC07	37,488,062	AT4G12290	AT4G12290	P
BnA02	bna3282	85.8	BoC07_37335580_301	BoC07	37,335,580	AT5G10940		
BnA02	bna3287	85.9	BoC07_36847163_301	BoC07	36,847,163	AT4G11910	AT4G11910	P
BnA02	bna3292	86	BoC07_36597360_301	BoC07	36,597,360	AT3G59550		
BnA02	bna5840	86.1	BoC06_1845858_301			AT4G11560	AT4G11560	P
BnA02	bna3463	86.1	BoC02_25884580_301					
BnA02	bna3957	86.4	BoC02_28752066_301	BoC02	28,752,066	AT3G09970		
BnA02	bna0734	86.4	BoC02_29924834_311	BoC02	29,924,834	AT3G44713		
BnA02	bna3536	86.7	BoC02_31889051_301	BoC02	31,889,051	AT2G03440		
BnA02	bna3535	87.1	BoC02_31914762_301	BoC02	31,914,762	AT1G31540		
BnA02	bna1053	88				AT3G61790		
BnA02	bna3529	88.9	BrA10_89244_100			AT4G06561	AT4G06561	P
BnA02	bna3551	90.6	BoC02_31483657_301	BoC02	31,483,657	AT2G07420		
BnA02	bna0846	90.6	BoC02_29637108_269	BoC02	29,637,108	AT4G02170		
BnA02	bna3297	92.3	BoC07_36458580_302			AT3G43083	AT3G43083	L
BnA02	bna3478	92.3	BoC02_26377496_250			AT1G37040		

BnA02	bna3451	93.1	BoC02_25351109_301			AT5G25920		
BnA02	bna3461	93.6	BoC06_14216429_301			AT3G30836	AT3G30836	L
BnA02	bna3266	93.6	BoScaffold000335_300181_301			AT5G02230		
BnA02	bna5859	93.9	BoScaffold000003_P1_338520_297			AT3G30716	AT3G30716	L
BnA02	bna1031	93.9	BoScaffold000512_57088_141			AT5G09350		
BnA02	bna3446	93.9	BoC02_25244717_155			AT5G33175		
BnA02	bna3498	93.9	BoC02_27193907_301			AT3G01513		
BnA02	bna3355	94.7	BrA02_4914943_269			AT3G45253	AT3G45253	M
BnA02	bna0269	96.4	BoScaffold000013_P2_1538530_301			AT1G64970		
BnA02	bna0870	96.4	BoScaffold000013_P2_1854300_287			AT3G45850	AT3G45850	M
BnA02	bna4751	96.8	BoScaffold000180_336494_302			AT4G34890		
BnA02	bna0267	96.8	BoScaffold000013_P2_2007823_301			AT4G13730		
BnA02	bna0265	96.8	BoScaffold000013_P2_2445372_302			AT5G14720	AT5G14720	R
BnA02	bna0858	98.4	BoScaffold000393_56192_294			AT5G18540	AT5G18540	R
BnA02	bna3963	98.8	BoC02_28352422_239	BoC02	28,352,422	AT1G24070	AT1G24070	B
BnA02	bna0464	99.3	BoC02_23932652_301			AT1G37040	AT1G37040	B
BnA02	bna5237	99.3	BoC07_36755053_277			AT1G42698	AT1G42698	C
BnA02	bna4705	99.6	BoC02_31504241_301	BoC02	31,504,241	AT1G45010	AT1G45010	C
BnA02	bna3258	99.9	BoC02_23426511_301			AT3G45050	AT3G45050	M
BnA02	bna3436	100.2	BoScaffold000031_P1_149878_301			AT3G31356	AT3G31356	M
BnA02	bna3460	100.2	BoC02_25833179_301			AT5G52110	AT5G52110	W
BnA02	bna3549	100.5	BoC07_32620548_299			AT5G58330	AT5G58330	W
BnA02	bna3485	100.8	BoC02_26694404_299			AT2G38185	AT2G38185	J
BnA02	bna3533	101.6	BoC05_9768588_287			AT2G47490	AT2G47490	J
BnA02	bna3444	102.4	BoC02_25162106_301			AT4G03450	AT4G03450	R
BnA02	bna3490	102.4	BoC02_26900603_297			AT4G04157	AT4G04157	R

BnA02	bna3267	102.5	BoScaffold000335_309595_223			AT5G35910		
BnA02	bna3479	102.6	BoC02_26439434_295			AT4G04316	AT4G04316	O
BnA02	bna3531	103.7	BoC05_7783846_301			AT4G06604	AT4G06604	O
BnA02	bna3546	104	BoC02_31630315_301	BoC02	31,630,315	AT5G46105		
BnA02	bna3544	104	BoC02_31689351_301	BoC02	31,689,351	ATMG00610		
BnA02	bna3539	104	BoC02_31838722_301	BoC02	31,838,722	AT3G59280	AT3G59280	N
BnA02	bna5839	104.6	BoC07_36544565_301			AT3G60170	AT3G60170	N
BnA02	bna0621	105.9	BoC02_35051270_295	BoC02	35,051,270	AT1G70250		
BnA02	bna4764	106.2	BoC05_8530889_302			AT5G62540		
BnA02	bna3528	106.5	BoC02_22655560_300			AT3G62475	AT3G62475	N
BnA02	bna4706	107.1	BoC02_31474599_301			AT2G13860	AT2G13860	G/H
BnA02	bna5969	107.4	BoC02_25568930_301			AT1G04590		
BnA02	bna3286	107.4	BoC07_36909176_301			AT4G11980		
BnA02	bna3294	107.4	BoC07_36545063_301			AT2G11950	AT2G11950	G/H
BnA02	bna3958	107.7	BoC02_28569158_301			AT2G11720	AT2G11720	G/H
BnA02	bna5824	108.5	BoC01_11317620_301			AT4G25030		
BnA02	bna4881	108.8	BoC07_36869769_222			AT3G02930	AT3G02930	F
BnA02	bna3450	109.1	BoC02_25309091_301					
BnA02	bna3462	109.1	BoC02_25869796_301			AT3G10180	AT3G10180	F
BnA02	bna0540	110.5	BoC02_35600452_301	BoC02	35,600,452	AT2G03080	AT2G03080	K
BnA02	BGO153	112.1	BrA02_22858298_238			AT3G25910		
BnA02	bna3552	118.4	BoC02_29369413_301			AT5G38370		
BnA02	bna0519	118.4	BoC07_18065200_298			AT1G26310		
BnA02	bna0587	119.8	BoC02_36701272_301	BoC02	36,701,272	AT2G03810	AT2G03810	K
BnA02	bna0743	122.8	BoC02_43650063_300			AT3G29642		
BnA02	BEN189A	123.7						

BnA02	CB10022B	133.6						
BnA02	BrGMS363	135.2	BrA02_27738746_153			AT5G35880		
BnA02	bna1514	135.5	BrA02_27812564_301			AT5G66200	AT5G66200	X
BnA02	bna3571	136.6	BoC02_41705763_301	BoC02	41,705,763	AT5G65205	AT5G65205	X
BnA02	bna3565	137.4	BoScaffold000121_P2_789519_301			AT5G63990	AT5G63990	X
BnA02	bna3564	138.2	BoScaffold000121_P2_775141_301			AT5G63940	AT5G63940	X
BnA02	bna3568	138.2	BrA02_27439547_301			AT5G65470	AT5G65470	X
BnA02	bna3569	138.5	BoC02_41757926_287	BoC02	41,757,926	AT3G52100		
BnA02	bna0558	138.8	BoScaffold000121_P2_271615_301			AT5G62680	AT5G62680	X
BnA02	bna3566	142.5	BoScaffold000514_31276_301			AT5G66190	AT5G66190	X
BnA02	BEN349	144.8						
BnA03	sN2032	0						
BnA03	bna0051	0.3	BoScaffold000091_1664085_301			AT5G61850		
BnA03	bna3560	0.3	BoScaffold000407_6294_301			AT5G28776		
BnA03	bna0288	0.3	BoC03_32383728_301	BoC03	32,383,728	AT4G39910		
BnA03	bna3780	4.9	BoC03_40477244_304	BoC03	40,477,244	AT5G52220		
BnA03	bna3779	5.9	BoC03_40489273_301	BoC03	40,489,273	AT1G26120	AT1G26120	B
BnA03	CALSSRB	5.9				AT1G26310	AT1G26310	B
BnA03	bna3791	6	BoC03_39863170_301	BoC03	39,863,170	AT2G30690		
BnA03	bna3790	6.1	BoC03_39888365_301	BoC03	39,888,365	AT4G27885		
BnA03	bna5949	6.2	BoC03_40197638_301	BoC03	40,197,638	AT1G75750		
BnA03	bna3789	6.3	BrA06_10261885_283			AT5G38700		
BnA03	bna3782	6.3	BrA08_17627997_303			AT1G26360	AT1G26360	B
BnA03	bna3778	6.3	BoC03_40501352_301	BoC03	40,501,352	AT5G37400		
BnA03	bna3777	6.3	BoC03_40530678_301	BoC03	40,530,678	AT5G03890		
BnA03	bna3775	6.3	BoC03_40591901_303	BoC03	40,591,901	AT4G32714		

BnA03	BoGMS819	9.1						
BnA03	BGO120	13.4				AT5G37680		
BnA03	bna0738	14.1	BoC06_4218118_188			AT1G27620	AT1G27620	B
BnA03	bna0639	14.8	BoC03_43830330_301	BoC03	43,830,330	AT1G27980	AT1G27980	B
BnA03	bna0583	18.9	BoC03_44543356_301	BoC03	44,543,356	AT1G28850	AT1G28850	B
BnA03	bna2666	19.2	BrA08_16479209_192			AT1G30130	AT1G30130	B
BnA03	BoGMS680	21.2						
BnA03	bna5138	21.7	BrA08_16478870_298			AT1G30130	AT1G30130	B
BnA03	bna3811	22.2	BoC03_45939776_300	BoC03	45,939,776	AT3G26480		
BnA03	BoGMS154	22.5						
BnA03	bna3810	24.1	BoC03_46851701_301	BoC03	46,851,701	AT4G38060	AT4G38060	U
BnA03	bna3809	24.1	BoC03_46882078_301	BoC03	46,882,078	AT4G37980	AT4G37980	U
BnA03	bna5847	24.1	BoC03_46968674_301	BoC03	46,968,674	AT4G37930	AT4G37930	U
BnA03	bna3783	25.2	BoC03_40257094_301			AT4G06510		
BnA03	bna4713	27.9				AT4G28640		
BnA03	bna3807	27.9	BoC03_47298054_301	BoC03	47,298,054	AT4G37560	AT4G37560	U
BnA03	bna3806	27.9	BoC03_47311414_301	BoC03	47,311,414	AT2G05980		
BnA03	bna3805	27.9	BoC03_47417434_295	BoC03	47,417,434	AT2G16440		
BnA03	bna3803	27.9	BoC03_47499541_300	BoC03	47,499,541	AT1G14660		
BnA03	bna4962	27.9	BoC03_47616595_270	BoC03	47,616,595	AT4G36930	AT4G36930	U
BnA03	bna3804	27.9	BoScaffold000009_P1_1530670_280			AT5G37150		
BnA03	bna0078	27.9	BoC03_47610468_156	BoC03	47,610,468	AT1G30070		
BnA03	bna0146	27.9	BoC03_47617425_301	BoC03	47,617,425	AT3G31442		
BnA03	BoGMS1295	28.2						
BnA03	bna3802	29.3	BoC03_47584059_280	BoC03	47,584,059	AT5G28490	AT5G28490	Q
BnA03	bna3801	29.3	BoC03_47620248_301	BoC03	47,620,248	AT4G28540		

BnA03	bna4714	29.3	BoC03_47341746_301	BoC03	47,341,746	AT5G29646	AT5G29646	Q
BnA03	bna3800	29.3	BoC03_47645315_307	BoC03	47,645,315	AT5G13181		
BnA03	bna3394	47.4	BoScaffold000098_115349_301			AT2G05610		
BnA03	BEN123	48.5	BrA03_6449351_263			AT5G52380	AT5G52380	W
BnA03	BN12AB	49.3	BrA03_6265845_277			AT5G53280	AT5G53280	W
BnA03	bna3612	50.3	BoC03_7268049_303	BoC03	7,268,049	AT1G16140		
BnA03	bna3613	50.6	BrA03_6261112_302			AT4G14350		
BnA03	bna3614	51.6	BoC03_7073696_301	BoC03	7,073,696	AT4G17520		
BnA03	bna3615	51.6	BoC03_6946602_301	BoC03	6,946,602	AT5G53740	AT5G53740	W
BnA03	bna3616	51.9	BoC03_6931807_301	BoC03	6,931,807	AT3G62060		
BnA03	bna4964	51.9	BoC03_7009435_301	BoC03	7,009,435	AT5G27680		
BnA03	bna3627	55.2	BoC03_6535141_301	BoC03	6,535,141	AT1G53070		
BnA03	bna3628	56.3	BoC03_6513447_305	BoC03	6,513,447	AT5G55000	AT5G55000	W
BnA03	bna3629	56.3	BoC03_6491259_301	BoC03	6,491,259	AT5G55045	AT5G55045	W
BnA03	BEN190A	60				AT5G55860	AT5G55860	W
BnA03	Na14-E02	61.1						
BnA03	Ol10-D03C	68.4						
BnA03	BoGMS1464A	72.1						
BnA03	BGO125	72.1				AT5G18400		
BnA03	bna3603	74.5	BoC03_4914984_300	BoC03	4,914,984	AT5G60790		
BnA03	bna3582	75.1	BrA03_4102152_300			AT5G20680	AT5G20680	R
BnA03	bna3588	76.2	BoC03_4552667_284	BoC03	4,552,667			
BnA03	bna3593	77.1	BoC03_4170187_301	BoC03	4,170,187	AT5G19990	AT5G19990	R
BnA03	bna5036	78.4	BoC03_3574955_301	BoC03	3,574,955	AT5G18010	AT5G18010	R
BnA03	bna4738	80.5	BoC03_2272422_301	BoC03	2,272,422	AT5G13550	AT5G13550	R
BnA03	bna5481	80.8	BoC03_2042670_301	BoC03	2,042,670	AT5G65650		

BnA03	bna5431	80.8	BrA03_2339872_263			AT5G12950	AT5G12950	R
BnA03	bna1289	80.8	BoC01_20781518_301			AT5G27680		
BnA03	bna3689	82	BoC03_1724642_215	BoC03	1,724,642	AT5G09390	AT5G09390	R
BnA03	bna3688	82	BoC03_1704921_301	BoC03	1,704,921	AT5G08790	AT5G08790	R
BnA03	bna3687	82.4	BoC03_1692240_301	BoC03	1,692,240	AT5G08630	AT5G08630	R
BnA03	bna3685	84.6	BrA03_1485228_301			AT5G08280	AT5G08280	R
BnA03	bna3681	84.6	BoC03_1256326_301	BoC03	1,256,326	AT5G07170	AT5G07170	R
BnA03	bna0107	86.2	BoC03_24221_231	BoC03	24,221	AT5G02030	AT5G02030	R
BnA03	bna0197	87.7	BrA03_80381_301			AT5G01640	AT5G01640	R
BnA03	bna0198	88	BrA03_135000_254			AT5G01365	AT5G01365	R
BnA03	BoGMS1201	90.9						
BnA03	BoGMS1373	91.5						
BnA03	BoGMS1320	94						
BnA03	BRMS-006	98.3						
BnA03	BnEMS993	100.5						
BnA03	BrGMS679	102.4	BrA03_43587_209			AT1G66020		
BnA03	BRMS-008	104.1	BrA03_53344_165			AT3G27390		
BnA03	sR12137IA	106.7	BrA03_1699524_355			AT5G09850		
BnA03	BoGMS1307	110.6						
BnA03	CB10413	132.8	BrA03_28648208_215	BrA03	28,648,208	AT1G05080		
BnA03	bna1538	135	BrA03_28222246_301	BrA03	28,222,246	AT4G31320	AT4G31320	U
BnA03	bna1542	135	BrA03_27821377_289	BrA03	27,821,377	AT1G06690		
BnA03	bna1545	135	BrA03_27612941_301	BrA03	27,612,941	AT5G42190		
BnA03	BGR54	135.8	BrA03_28268108_129	BrA03	28,268,108			
BnA03	BrGMS509	136.6	BrA03_27239138_278	BrA03	27,239,138	AT4G02075		
BnA03	bna5278	137.3	BrA03_27399085_301	BrA03	27,399,085	AT1G66750		

BnA03	bna5279	137.3	BrA03_27181901_301	BrA03	27,181,901	AT1G35146		
BnA03	bna1546	137.6	BrA03_27561648_301	BrA03	27,561,648	AT4G30074	AT4G30074	U
BnA03	bna1548	137.6	BrA03_27445975_306	BrA03	27,445,975	AT4G29790	AT4G29790	U
BnA03	bna1549	137.9	BrA03_27385091_301	BrA03	27,385,091	AT5G26120		
BnA03	bna1551	138.4	BrA03_27104883_301	BrA03	27,104,883	AT4G28890	AT4G28890	U
BnA03	bna1552	139.5	BrA03_26637960_298	BrA03	26,637,960	AT4G27657	AT4G27657	U
BnA03	bna1553	139.5	BrA03_26608007_293	BrA03	26,608,007	AT1G37070		
BnA03	bna1802	158.7	BrA03_501255_301	BrA03	501,255	AT5G38150		
BnA03	Na10-G10	160.1	BrA03_672353_135	BrA03	672,353	AT5G04150	AT5G04150	R
BnA03	bna5585	162	BrA03_1596241_300	BrA03	1,596,241	AT5G08570	AT5G08570	R
BnA03	sR12137IB	162.3	BrA03_1699524_355	BrA03	1,699,524	AT5G09850	AT5G09850	R
BnA03	BEN320	162.8				AT5G12120		
BnA03	BoGMS539	164.7	BrA03_1821839_183	BrA03	1,821,839	AT5G10470	AT5G10470	R
BnA03	BGO029	166.6	BrA03_3156872_138	BrA03	3,156,872	AT5G16500	AT5G16500	R
BnA03	BoGMS1464B	167.4						
BnA03	bna1748	170.8	BrA03_4257090_301	BrA03	4,257,090	AT5G22090	AT5G22090	R
BnA03	bna5581	170.8	BrA03_4339043_301	BrA03	4,339,043	AT5G22450	AT5G22450	R
BnA03	bna1747	170.8	BrA03_4399435_301	BrA03	4,399,435	AT5G22760	AT5G22760	R
BnA03	bna1743	173.9	BrA03_5006694_301	BrA03	5,006,694	AT5G58300	AT5G58300	W
BnA03	bna1741	173.9	BrA03_5083348_301	BrA03	5,083,348	AT5G57800	AT5G57800	W
BnA03	bna0462	174.4	BoC03_5872410_301			AT5G57500	AT5G57500	W
BnA03	bna1740	174.9	BrA03_5160080_301	BrA03	5,160,080	AT5G57590	AT5G57590	W
BnA03	bna1738	176.6	BrA03_5415096_301	BrA03	5,415,096	AT5G56530	AT5G56530	W
BnA03	bna1736	176.6	BrA03_5523389_301	BrA03	5,523,389	AT5G56250	AT5G56250	W
BnA03	BEN190B	177.3				AT5G55860	AT5G55860	W
BnA03	bna1730	178.4	BrA03_6018284_301	BrA03	6,018,284	AT5G54170	AT5G54170	W

BnA03	BN12AA	179.2	BrA03_6265845_277	BrA03	6,265,845	AT5G53280	AT5G53280	W
BnA03	BEN251	179.2	BrA03_6283089_162	BrA03	6,283,089	AT5G53280	AT5G53280	W
BnA03	bna1727	179.7	BrA03_6286513_301	BrA03	6,286,513	AT5G53260	AT5G53260	W
BnA03	BnEMS1084	181.6	BrA03_6289126_377	BrA03	6,289,126	AT5G53220	AT5G53220	W
BnA03	bna5580	183.5	BrA03_6423348_301	BrA03	6,423,348	AT5G65660		
BnA03	bna1722	183.8	BrA03_6758687_302	BrA03	6,758,687	AT4G06634		
BnA03	bna1717	184.9	BrA03_7094848_303	BrA03	7,094,848	AT4G02910		
BnA03	bna1716	185.6	BrA03_7178701_155	BrA03	7,178,701	AT2G30690	AT2G30690	I
BnA03	bna1715	185.6	BrA03_7225004_272	BrA03	7,225,004	AT2G30880	AT2G30880	I
BnA03	bna1713	186.1	BrA03_7284240_301	BrA03	7,284,240	AT2G31100	AT2G31100	J
BnA03	bna1712	187.5	BrA03_7521574_301	BrA03	7,521,574	AT1G05520		
BnA03	bna1711	187.5	BrA03_7618218_224	BrA03	7,618,218	AT5G28463		
BnA03	bna1709	187.8	BrA03_7766266_309	BrA03	7,766,266	AT2G12640		
BnA03	BGR80	190	BrA03_7377352_124	BrA03	7,377,352	AT2G31510	AT2G31510	J
BnA03	bna5064	191.4	BrA03_7793224_299	BrA03	7,793,224	AT2G32850	AT2G32850	J
BnA03	bna1706	191.4	BrA03_7935716_301	BrA03	7,935,716	AT2G33830	AT2G33830	J
BnA03	Na12-E02A	191.9	BrA03_7953653_103	BrA03	7,953,653	AT1G17310		
BnA03	bna1700	193.5	BrA03_8396675_308	BrA03	8,396,675	AT2G36010	AT2G36010	J
BnA03	bna5195	193.5	BrA03_8585046_301	BrA03	8,585,046	AT2G27200		
BnA03	bna0092	193.5	BrA03_8627204_301	BrA03	8,627,204	AT2G36910	AT2G36910	J
BnA03	bna0098	193.5	BrA03_8629844_303	BrA03	8,629,844	AT2G36910	AT2G36910	J
BnA03	bna1842	193.8	BrA03_8940224_301	BrA03	8,940,224	AT2G38020	AT2G38020	J
BnA03	BGR75	195.1	BrA03_8963189_141	BrA03	8,963,189	AT2G38090	AT2G38090	J
BnA03	bna5284	199.9	BrA03_9243550_307	BrA03	9,243,550	AT2G38840	AT2G38840	J
BnA03	bna1846	199.9	BrA03_9250681_288	BrA03	9,250,681	AT3G54620		
BnA03	bna1847	199.9	BrA03_9258827_301	BrA03	9,258,827	AT2G38910	AT2G38910	J

BnA03	bna1849	199.9	BoC03_10808656_301			AT2G39260	AT2G39260	J
BnA03	bna5880	199.9	BrA03_9382131_301	BrA03	9,382,131	AT3G49700		
BnA03	bna1851	200.5	BrA03_9627215_301	BrA03	9,627,215	AT2G40004	AT2G40004	J
BnA03	bna5590	200.5	BrA03_9641522_301	BrA03	9,641,522	AT5G13485		
BnA03	bna1852	200.5	BrA03_9657714_299	BrA03	9,657,714	AT2G40080	AT2G40080	J
BnA03	bna5577	201.6	BrA03_10455016_301	BrA03	10,455,016	AT2G43500	AT2G43500	J
BnA03	bna1686	202.9	BrA03_10969259_301	BrA03	10,969,259	AT5G45820		
BnA03	bna1692	203.2	BrA03_10716163_301	BrA03	10,716,163	AT2G44640	AT2G44640	J
BnA03	bna1691	203.2	BrA03_10761610_301	BrA03	10,761,610	AT2G44940	AT2G44940	J
BnA03	bna0057	203.2	BrA03_10900451_301	BrA03	10,900,451	AT2G45660	AT2G45660	J
BnA03	bna5575	204.7	BrA03_11319872_301	BrA03	11,319,872	AT1G21860		
BnA03	bna1681	204.7	BrA03_11365646_301	BrA03	11,365,646	AT5G54320		
BnA03	bna1678	204.7	BrA03_11499674_302	BrA03	11,499,674	AT5G28640		
BnA03	bna1676	204.7	BrA03_11593119_301	BrA03	11,593,119	AT2G27050	AT2G27050	I
BnA03	bna0238	204.7	BoC03_14372308_301			AT4G00040		
BnA03	bna1677	204.7	BrA03_11531312_301	BrA03	11,531,312	AT2G27330	AT2G27330	I
BnA03	bna4986	204.7	BrA03_11534180_301	BrA03	11,534,180	AT2G27310	AT2G27310	I
BnA03	bna1682	204.7	BrScaffold000167_105752_301			AT2G29320	AT2G29320	I
BnA03	bna1685	205	BrA03_11070726_301	BrA03	11,070,726	AT5G54100		
BnA03	bna1683	205	BrA03_11218618_301	BrA03	11,218,618	AT2G47760	AT2G47760	J
BnA03	bna1674	205	BrA03_11685935_300	BrA03	11,685,935	AT2G40100	AT2G40100	J
BnA03	bna1673	205.6	BrA03_12316922_301	BrA03	12,316,922	AT5G19900		
BnA03	bna1232	205.6	BrA03_12548931_305	BrA03	12,548,931	AT4G10180	AT4G10180	P
BnA03	bna1670	206	BrA03_12932549_301	BrA03	12,932,549	AT4G12120	AT4G12120	P
BnA03	bna1669	206.1	BrA03_12933240_294	BrA03	12,933,240	AT1G42745		
BnA03	bna1667	206.2	BrA03_13160678_301	BrA03	13,160,678	AT4G04470	AT4G04470	O

BnA03	bna1665	206.3	BrA03_13257273_260	BrA03	13,257,273	AT5G35240		
BnA03	bna4985	206.4	BrA03_12990760_301	BrA03	12,990,760	AT2G33940		
BnA03	bna1663	206.5	BrA03_13365862_301	BrA03	13,365,862	AT4G03430	AT4G03430	O
BnA03	bna1662	206.6	BrA03_13425088_301	BrA03	13,425,088	AT4G03260	AT4G03260	O
BnA03	bna1660	206.6	BrA03_13579698_250	BrA03	13,579,698	AT4G02725	AT4G02725	O
BnA03	bna2170	206.9	BrA05_3828439_301			AT5G21160		
BnA03	bna4844	207.2	BrA03_13298884_301	BrA03	13,298,884	AT1G61660		
BnA03	BEN133	209.4	BrA03_11054113_157			AT4G00230		
BnA03	BRAS051B	209.9						
BnA03	BRAS087B	211.3				AT5G16220		
BnA03	bna5446	215.5	BrA03_13678885_301	BrA03	13,678,885	AT4G02410	AT4G02410	O
BnA03	bna5903	215.5	BrA07_6044789_301			AT4G16810		
BnA03	bna1659	216.3	BrA03_13658610_263	BrA03	13,658,610	AT4G02480	AT4G02480	O
BnA03	bna1658	216.6	BrA03_13707058_303	BrA03	13,707,058	AT4G02920		
BnA03	bna1656	216.6	BoC03_18995783_301			AT4G01950	AT4G01950	O
BnA03	bna1650	218.1	BrA03_14292099_301	BrA03	14,292,099	AT3G01750	AT3G01750	F
BnA03	BEN25	221.9	BrA03_14628164_132	BrA03	14,628,164	AT3G03550	AT3G03550	F
BnA03	bna1643	224.3	BoScaffold000040_1433582_301			AT3G05610	AT3G05610	F
BnA03	bna1638	225.4	BrA03_15368607_301	BrA03	15,368,607	AT4G24670		
BnA03	bna1637	225.4	BrA03_15421652_301	BrA03	15,421,652	AT3G06940	AT3G06940	F
BnA03	BoGMS1425	226.3						
BnA03	BrGMS216	227.7	BrA03_16125257_283	BrA03	16,125,257	AT2G24700		
BnA03	BrGMS217	228.8	BrA03_16137511_129	BrA03	16,137,511	AT5G02580		
BnA03	bna1631	230.4	BrA03_16229444_301	BrA03	16,229,444	AT3G11510	AT3G11510	F
BnA03	BGR35	233.2	BrA03_16137523_113	BrA03	16,137,523	AT5G02580		
BnA04	bna1167	0	BrA03_15882_301			AT5G41755		

BnA04	bna0702	0	BrA01_22363559_293			AT5G11840	AT5G11840	R
BnA04	bna0817	0.6	BoC04_27594573_294			AT1G21350		
BnA04	bna0556	3.6	BoC08_35189595_301			AT5G06810	AT5G06810	R
BnA04	sN13034	4.2	BrA04_6624964_137			AT5G27100		
BnA04	bna3986	5.2	BoScaffold000192_253750_301			AT5G65830		
BnA04	BRAS021	5.9						
BnA04	Na10-C01A	8.6						
BnA04	BoGMS829	8.6						
BnA04	bna2052	9.2	BoC04_29405992_301			AT3G28400		
BnA04	bna0697	9.2	BoC04_29321703_301			ATMG00510		
BnA04	BnEMS1184	32.8	BrA04_276812_389	BrA04	276,812	AT3G62840	AT3G62840	N
BnA04	bna1810	34.1	BrA04_600073_301	BrA04	600,073	AT3G09400		
BnA04	bna1819	36	BrA04_1213046_301	BrA04	1,213,046	AT3G59820	AT3G59820	N
BnA04	bna1822	37.1	BrA04_1457537_301	BrA04	1,457,537	AT3G28870		
BnA04	bna1827	38.7	BrA04_1735132_301	BrA04	1,735,132	AT3G57690	AT3G57690	N
BnA04	bna1828	38.7	BrA04_1779999_301	BrA04	1,779,999	AT3G57610	AT3G57610	N
BnA04	bna5083	38.7	BrA04_1894868_214	BrA04	1,894,868	AT3G25225		
BnA04	bna1830	38.7	BrA04_1943599_301	BrA04	1,943,599	AT5G33070		
BnA04	bna1832	39.1	BrA04_2048437_301	BrA04	2,048,437	AT3G53590		
BnA04	bna5913	39.5	BrA04_2199893_301	BrA04	2,199,893	AT3G22100		
BnA04	bna4845	39.5	BrA04_2230696_301	BrA04	2,230,696	AT3G56550	AT3G56550	N
BnA04	bna1834	39.5	BrA04_2309487_289	BrA04	2,309,487	AT3G56360	AT3G56360	N
BnA04	bna5592	40.3	BrA04_2497900_301	BrA04	2,497,900	AT3G55850	AT3G55850	N
BnA04	bna1860	40.6	BrA04_2650277_301	BrA04	2,650,277	AT3G55610	AT3G55610	N
BnA04	bna1861	41.4	BrA04_2688974_301	BrA04	2,688,974	AT3G56620		
BnA04	bna5196	41.4	BrA04_2933073_218	BrA04	2,933,073	AT3G47810		

BnA04	bna1865	41.4	BrA04_2968931_301	BrA04	2,968,931	AT3G29078		
BnA04	bna5593	42.2	BrA04_2735498_301	BrA04	2,735,498	AT3G55440	AT3G55440	N
BnA04	sR9411	45						
BnA04	BGR98	48.4				AT3G52770	AT3G52770	N
BnA04	CB10347	48.7	BrA04_4267194_184	BrA04	4,267,194			
BnA04	BnEMS1116	51.5	BrA04_5237214_137	BrA04	5,237,214	AT5G41380		
BnA04	bna5342	55.2	BoC04_7704927_262			AT5G35540		
BnA04	bna5868	55.3	BrA04_4112310_301	BrA04	4,112,310	AT5G53390		
BnA04	bna5595	55.4	BrA04_4259512_185	BrA04	4,259,512	AT3G52220	AT3G52220	N
BnA04	bna1876	55.4	BrA04_4263813_301	BrA04	4,263,813	AT3G52200	AT3G52200	N
BnA04	bna1885	55.4	BrA04_4823706_301	BrA04	4,823,706	AT4G14070		
BnA04	bna1884	55.4	BrA04_4932863_301	BrA04	4,932,863	AT2G19800		
BnA04	bna1882	55.4	BrA04_5287304_301	BrA04	5,287,304	AT4G23260		
BnA04	bna5881	55.4	BrA06_5428029_222			AT4G04880		
BnA04	bna1888	56.5	BrA04_5985363_301	BrA04	5,985,363	AT2G05260		
BnA04	bna1893	58.2	BrA04_7035364_301	BrA04	7,035,364	AT4G26900		
BnA04	bna1901	58.5	BrA04_7588670_301	BrA04	7,588,670	AT5G40240		
BnA04	bna1895	59.1	BrA04_7233828_301	BrA04	7,233,828	AT3G29010		
BnA04	bna1900	59.1	BrA04_7520180_301	BrA04	7,520,180	AT4G13980		
BnA04	bna1894	59.1	BrA08_19316263_301			AT5G66260		
BnA04	bna3976	59.9	BoScaffold000192_615990_301			AT2G22155		
BnA04	bna5380	60.2	BrA04_7643434_301	BrA04	7,643,434	AT3G13772		
BnA04	BEN98	61.8	BrA04_7920797_150	BrA04	7,920,797	AT5G40660		
BnA04	pMR181A	69.5				AT1G47765		
BnA05	BnGMS584B	0				AT2G05780		
BnA05	BGR74	2.5				AT2G05990		

BnA05	BrGMS406	3.3				AT3G24760	AT3G24760	F
BnA05	Ra3-D04	3.3						
BnA05	Na10-A09	3.3						
BnA05	bna1616	4.1	BrA03_18834614_301	BrA03	18,834,614	AT3G21760	AT3G21760	F
BnA05	bna1615	4.1	BrA03_18836652_294	BrA03	18,836,652	AT3G21760	AT3G21760	F
BnA05	bna1617	4.1	BoC06_34818725_300			AT5G54650		
BnA05	bna1613	4.4	BoC03_25470736_300			AT1G47320		
BnA05	bna1608	4.7	BrA03_20080950_301	BrA03	20,080,950	AT2G07690		
BnA05	bna1601	5.3	BrA03_20667595_269	BrA03	20,667,595	AT2G16440		
BnA05	bna5566	10.4	BrA03_21198346_301	BrA03	21,198,346	AT5G61010		
BnA05	bna4349	11.2	BoC06_37133106_225			AT5G06710		
BnA05	bna1599	12.3	BrA03_21504721_301	BrA03	21,504,721	AT3G49790		
BnA05	bna1590	16	BrA03_22300522_303	BrA03	22,300,522	AT4G15530		
BnA05	CB10034	19.4				AT5G27840		
BnA05	bna2843	24	BrA03_23127508_301	BrA03	23,127,508	AT4G18010	AT4G18010	U
BnA05	bna1589	24.3	BrA03_23467471_300	BrA03	23,467,471			
BnA05	bna4642	24.3	BrA03_23503230_234	BrA03	23,503,230	AT3G03940		
BnA05	bna2842	24.4	BrA03_23061650_301	BrA03	23,061,650	AT5G38710		
BnA05	bna5911	24.5	BoC06_41088998_252			AT4G18550	AT4G18550	U
BnA05	bna5886	24.5	BrA03_23160521_301	BrA03	23,160,521	AT5G24870		
BnA05	bna0038	24.5	BrA03_23526906_301	BrA03	23,526,906	AT4G18960	AT4G18960	U
BnA05	bna0037	24.5	BrA03_23528679_301	BrA03	23,528,679	AT4G18960	AT4G18960	U
BnA05	bna1585	24.5	BrA03_23800118_301	BrA03	23,800,118	AT5G05770		
BnA05	bna4840	25.9	BrA03_23887523_299	BrA03	23,887,523	AT1G08650		
BnA05	bna1584	26.9	BrA03_24365533_301	BrA03	24,365,533	AT5G13200		
BnA05	pMR181B	27.7						

BnA05	bna1575	28.8	BrA03_25047900_301	BrA03	25,047,900	AT4G23370	AT4G23370	U
BnA05	bna1572	29.7	BrA03_25340743_301	BrA03	25,340,743	AT5G23750		
BnA05	bna5121	29.7	BrA03_25340743_301	BrA03	25,340,743	AT5G23750		
BnA05	bna5097	29.7	BrA03_25499459_223	BrA03	25,499,459	AT5G56550		
BnA05	bna4838	29.7	BrA03_25504906_301	BrA03	25,504,906	AT4G19925		
BnA05	BGR56	30.6				AT4G23740	AT4G23740	U
BnA05	BEN83	30.6				AT4G24240	AT4G24240	U
BnA05	BnEMS146	30.6				AT4G24240	AT4G24240	U
BnA05	bna1571	31.7	BrA03_25614556_301	BrA03	25,614,556	AT5G28468		
BnA05	bna4389	32.2	BoC06_43594658_301			AT4G24680	AT4G24680	U
BnA05	bna1567	32.2	BoC06_43730699_301			AT4G25120	AT4G25120	U
BnA05	bna0021	33.1	BrA03_26163719_301	BrA03	26,163,719	AT4G26010	AT4G26010	U
BnA05	bna5444	34	BrA03_26272710_301	BrA03	26,272,710	AT4G26300	AT4G26300	U
BnA05	BnGMS265	50	BrA05_22210227_345	BrA05	22,210,227	AT1G70850		
BnA05	sR9477	51.2	BrA05_22498503_263	BrA05	22,498,503	AT4G20000		
BnA05	bna2038	52.3	BrA05_22292108_301	BrA05	22,292,108	AT3G09660	AT3G09660	F
BnA05	bna5616	53	BrA05_22427562_301	BrA05	22,427,562	AT3G09100	AT3G09100	F
BnA05	bna2037	53	BrA05_22427562_301	BrA05	22,427,562	AT3G09100	AT3G09100	F
BnA05	bna2035	54.4	BrA05_22580798_289	BrA05	22,580,798	AT5G23430		
BnA05	bna5092	54.4	BrA05_22631736_297	BrA05	22,631,736	AT3G08030	AT3G08030	F
BnA05	BGR38	55.2	BrA05_22561185_146	BrA05	22,561,185	AT3G32305		
BnA05	bna2030	57.9	BoC05_32350587_297	BoC05	32,350,587	AT3G07130	AT3G07130	F
BnA05	bna5450	57.9	BoC05_32350689_300	BoC05	32,350,689	AT3G07130	AT3G07130	F
BnA05	bna2029	59	BoC05_32515770_301	BoC05	32,515,770	AT3G06930	AT3G06930	F
BnA05	bna5091	59.5	BoC05_32701848_301	BoC05	32,701,848	AT3G06483		
BnA05	bna2033	60.6	BrA05_22766863_301	BrA05	22,766,863	AT5G30545		

BnA05	bna2028	61.1	BrA05_23020982_301	BrA05	23,020,982	AT3G06860	AT3G06860	F
BnA05	bna5288	61.6	BrA05_23082022_302	BrA05	23,082,022	AT3G66658	AT3G66658	F
BnA05	bna2026	61.9	BrA05_23162355_301	BrA05	23,162,355	AT3G06490	AT3G06490	F
BnA05	bna1476	62.2	BrA02_23247623_301			AT3G06330	AT3G06330	F
BnA05	bna5090	63.3	BrA05_23207558_245	BrA05	23,207,558	AT3G23620		
BnA05	BRMS-007	65.5	BrA05_23268922_132	BrA05	23,268,922	AT4G29100		
BnA05	bna5615	67.1	BrA05_23385467_301	BrA05	23,385,467	AT3G05240	AT3G05240	F
BnA05	bna2024	67.4	BrA05_23425922_301	BrA05	23,425,922	AT3G05165	AT3G05165	F
BnA05	bna2025	68.1	BrA05_23502843_301	BrA05	23,502,843	AT3G04980	AT3G04980	F
BnA05	bna0306	68.8	BrScaffold000203_24232_301			AT3G04730	AT3G04730	F
BnA05	bna0286	68.8	BrScaffold000191_59457_298			AT3G04450	AT3G04450	F
BnA05	BoGMS1059	70.6						
BnA05	bna1515	72.2	BrA02_26946155_301			AT3G04050	AT3G04050	F
BnA05	bna5991	72.2	BoScaffold000460_96754_301			AT4G33400		
BnA05	bna1516	74.6	BoScaffold000460_119035_272			AT3G03860	AT3G03860	F
BnA05	bna5371	76.9	BoScaffold000461_93279_301			AT3G03770	AT3G03770	F
BnA05	bna0321	78.8	BrA02_26686182_208			AT5G47150		
BnA05	bna1511	80.7	BrA02_26644147_301			AT3G03090	AT3G03090	F
BnA05	bna2023	82.7	BrA05_23533846_301	BrA05	23,533,846	AT3G14760		
BnA05	BEN200A	83.5	BrA05_23831506_214	BrA05	23,831,506	AT5G15080		
BnA06	bna5650	0	BrA06_2993650_301	BrA06	2,993,650	AT1G08650	AT1G08650	A
BnA06	bna5651	0.3	BrA06_3114668_301	BrA06	3,114,668	AT4G05095		
BnA06	bna4853	0.6	BrA06_3203762_301	BrA06	3,203,762	ATMG00160		
BnA06	bna0130	0.6	BrA06_3279046_301	BrA06	3,279,046	AT1G09570	AT1G09570	A
BnA06	bna2267	0.6	BrA06_3279046_301	BrA06	3,279,046	AT1G09570	AT1G09570	A
BnA06	bna5251	3.6	BrA06_3651623_301	BrA06	3,651,623	AT5G18670		

BnA06	bna2259	3.6	BrA06_3651623_301	BrA06	3,651,623	AT5G18670		
BnA06	sN2837	4.2	BrA06_3578834_153	BrA06	3,578,834	AT5G64710		
BnA06	Na12-D08	5	BrA06_3726880_69	BrA06	3,726,880	AT1G10600	AT1G10600	A
BnA06	bna2257	5.9	BrA06_3759170_301	BrA06	3,759,170	AT1G10650	AT1G10650	A
BnA06	bna5653	5.9	BrA06_3759170_301	BrA06	3,759,170	AT1G10650	AT1G10650	A
BnA06	bna2256	5.9	BrA06_3818571_301	BrA06	3,818,571	AT1G10840	AT1G10840	A
BnA06	bna5040	6.3	BrA06_3867444_242	BrA06	3,867,444	AT1G11040	AT1G11040	A
BnA06	bna2254	6.3	BrA06_3867444_242	BrA06	3,867,444	AT1G11040	AT1G11040	A
BnA06	bna2251	8	BrA06_4035954_301	BrA06	4,035,954	AT2G05710		
BnA06	bna5392	8	BrA06_4026624_322	BrA06	4,026,624	AT5G46325		
BnA06	bna4852	9.4	BrA06_4428809_222	BrA06	4,428,809	AT1G12730	AT1G12730	A
BnA06	Ol10-D01	11.3	BrA06_4399748_208	BrA06	4,399,748	AT2G31250		
BnA06	bna2245	14	BoScaffold000127_203799_227			AT1G14040	AT1G14040	A
BnA06	bna5455	14.1	BoScaffold000127_203799_227			AT1G14040	AT1G14040	A
BnA06	bna4062	14.2	BoC05_3725941_301			AT1G12200		
BnA06	bna2247	14.3	BrA06_4534080_301	BrA06	4,534,080	AT4G05430		
BnA06	bna2246	14.3	BrA06_4738919_263	BrA06	4,738,919	AT1G11990		
BnA06	bna5657	14.6	BrA06_5577784_301	BrA06	5,577,784	AT1G14540	AT1G14540	A
BnA06	bna2279	16.2	BrA06_6520150_300	BrA06	6,520,150	AT1G16970	AT1G16970	A
BnA06	bna5343	17.1	BrA06_6691478_301	BrA06	6,691,478	AT1G17440	AT1G17440	A
BnA06	bna5658	17.4	BrA06_6878642_301	BrA06	6,878,642	AT1G17930	AT1G17930	A
BnA06	bna5932	20.4	BrA09_16121849_288			AT3G56710	AT3G56710	N
BnA06	bna5887	20.7	BrA09_16121849_288			AT3G56710	AT3G56710	N
BnA06	BoGMS1230	30.1						
BnA06	BoGMS1203	34.7						
BnA06	BGR34	36.3	BrA06_11864505_137	BrA06	11,864,505	AT5G03940		

BnA06	BRMS-049A	36.6	BrA06_12580768_141	BrA06	12,580,768	AT5G28640		
BnA06	BEN253	36.9	BrA06_9151367_207			AT3G49640	AT3G49640	M
BnA06	bna5132	37.9	BrA06_10134979_311			AT3G48010	AT3G48010	M
BnA06	bna3796	37.9	BoC03_39601720_301			AT3G47610	AT3G47610	M
BnA06	bna0279	38.2	BrScaffold000796_970_278			AT3G44160	AT3G44160	M
BnA06	bna1897	39.2	BrScaffold000178_232155_303			AT5G12920		
BnA06	bna3792	39.9	BoC03_39765391_301			AT3G31950	AT3G31950	M
BnA06	bna4342	40.5	BrA06_11667624_301	BrA06	11,667,624	AT2G29880		
BnA06	bna5781	40.9	BrScaffold000244_26717_302			AT2G15410		
BnA06	bna5142	42	BrA06_19593663_299			AT5G62880	AT5G62880	X
BnA06	bna0335	42	BrA06_19609664_301			AT5G62900	AT5G62900	X
BnA06	bna2318	43	BrA06_14265955_298	BrA06	14,265,955	AT5G63420	AT5G63420	X
BnA06	bna4958	43.6	BrA06_14594986_291	BrA06	14,594,986	AT5G64030	AT5G64030	X
BnA06	bna2296	48.1	BrA06_16620834_301	BrA06	16,620,834	AT5G23610	AT5G23610	Q
BnA06	bna2297	49.2	BrA06_16659915_301	BrA06	16,659,915	AT5G23740	AT5G23740	Q
BnA06	bna2580	50.3	BrA07_1396686_301			AT5G26680		
BnA06	bna4990	51.7	BoC06_35346921_292			AT5G25960	AT5G25960	Q
BnA06	bna2305	51.7	BrA06_17822965_301	BrA06	17,822,965	AT5G25960	AT5G25960	Q
BnA06	bna2307	51.7	BrA06_18116757_177	BrA06	18,116,757	AT5G26920	AT5G26920	Q
BnA06	bna5898	52.2	BrA06_18657413_302	BrA06	18,657,413	AT4G01950		
BnA06	bna5877	52.2	BrA06_18657413_302	BrA06	18,657,413	AT4G01950		
BnA06	BGR45	53.2	BrA06_17992203_105	BrA06	17,992,203	AT5G11580		
BnA06	BoGMS314	53.5	BrA06_18069372_115	BrA06	18,069,372	AT4G08900		
BnA06	bna2311	57.2	BoScaffold000153_664915_261			AT5G27970	AT5G27970	Q
BnA06	bna5395	60.3	BoScaffold000153_664915_261			AT5G27970	AT5G27970	Q
BnA06	bna2312	60.6	BrA06_18772227_301	BrA06	18,772,227	AT5G49430		

BnA06	bna5665	60.9	BrA06_21475708_303	BrA06	21,475,708	AT5G49120	AT5G49120	V
BnA06	bna2336	60.9	BrA06_21512860_268	BrA06	21,512,860	AT4G22680		
BnA06	bna4655	61.2	BrA06_21247253_219	BrA06	21,247,253	AT5G48520	AT5G48520	V
BnA06	bna2339	61.5	BrA06_21171937_301	BrA06	21,171,937	AT5G48270	AT5G48270	V
BnA06	BnEMS59	64.2	BrA06_21942889_171	BrA06	21,942,889	AT3G28920	AT3G28920	Q
BnA06	BnEMS60	64.5	BrA06_21942889_171	BrA06	21,942,889	AT3G28920	AT3G28920	Q
BnA06	bna5922	66.5	BrA06_21676878_301	BrA06	21,676,878	AT3G30340	AT3G30340	L
BnA06	bna2342	69.7	BrA06_22104886_300	BrA06	22,104,886	AT3G28470	AT3G28470	L
BnA06	bna5298	76.1	BrA06_22807945_301	BrA06	22,807,945	AT3G26370	AT3G26370	L
BnA06	bna2344	76.1	BrA06_22807945_301	BrA06	22,807,945	AT3G26370	AT3G26370	L
BnA06	bna2346	76.1	BrA06_22896930_301	BrA06	22,896,930	AT3G26130	AT3G26130	L
BnA06	sR12156A	77.7	BrA06_23032511_177	BrA06	23,032,511	AT5G16580		
BnA06	bna5923	79.1	BrA06_23656979_285	BrA06	23,656,979	AT2G26650		
BnA06	bna0629	80.5	BoC06_27820808_301			AT5G46100	AT5G46100	V
BnA06	bna2358	80.5	BrA06_24695710_301	BrA06	24,695,710	AT3G48770		
BnA06	BEN278B	81.6				AT5G47210	AT5G47210	V
BnA06	BnEMS695	85.1	BrA06_26241942_212	BrA06	26,241,942	AT4G38250	AT4G38250	U
BnA06	CB10330	86.1	BrA06_26104700_148	BrA06	26,104,700	AT4G38950	AT4G38950	U
BnA06	bna2364	87.4	BrA06_26039984_299	BrA06	26,039,984	AT4G39210	AT4G39210	U
BnA06	bna5347	87.4	BrA06_26039984_299	BrA06	26,039,984	AT4G39210	AT4G39210	U
BnA07	bna0826	0	BoScaffold000462_60360_302			AT3G06370	AT3G06370	F
BnA07	bna0882	2.9	BoScaffold000460_57388_301			AT3G03970	AT3G03970	F
BnA07	bna0084	2.9	BoC05_32774889_301			AT3G03450	AT3G03450	F
BnA07	bna4140	2.9	BoScaffold000461_38186_301			AT2G42590		
BnA07	bna0837	3.4	BoC05_32800593_301			AT3G13140		
BnA07	BEN186	3.9						

BnA07	bna0860	5.2	BoScaffold000485_60469_265			AT3G02832	AT3G02832	F
BnA07	bna1214	5.2	BrA04_9976097_301			AT5G42645		
BnA07	BEN200B	6.3						
BnA07	bna0866	30.5	BoC07_40244944_301			AT2G47800		
BnA07	bna3850	31.6	BoC07_26012560_223	BoC07	26,012,560			
BnA07	bna1038	32.4	BoScaffold000009_P1_226299_301					
BnA07	bna3842	33.2	BoC07_25410267_301	BoC07	25,410,267	AT4G04313		
BnA07	bna3846	33.3	BoC07_25858003_301	BoC07	25,858,003	AT1G49190	AT1G49190	C
BnA07	bna3848	33.4	BoC07_25928145_301	BoC07	25,928,145	AT1G49040	AT1G49040	C
BnA07	bna3851	33.5	BoC07_26057612_301	BoC07	26,057,612	AT3G26483		
BnA07	bna0888	33.6	BoC07_26256585_301	BoC07	26,256,585	AT1G48930	AT1G48930	C
BnA07	bna4502	33.6	BoC07_25424488_301	BoC07	25,424,488	AT1G30560		
BnA07	bna5067	33.6	BoC07_25424488_301	BoC07	25,424,488	AT1G30560		
BnA07	bna3841	35.9	BoC07_13552421_301			AT1G40089		
BnA07	bna0504	37	BoC07_25038543_302	BoC07	25,038,543	AT3G42700		
BnA07	bna0771	37.6	BoC07_13343898_301			AT5G57350		
BnA07	bna0505	39.2	BoC07_24768318_301	BoC07	24,768,318	AT4G36925		
BnA07	BGO161	40.8				AT5G67060		
BnA07	BEN391A	40.8						
BnA07	BGO160B	41.1				AT1G52640	AT1G52640	C
BnA07	bna0951	43	BoC07_21887638_301	BoC07	21,887,638			
BnA07	bna4370	43.8	BoC06_41160354_281					
BnA07	BoGMS490	44.6				AT3G06060		
BnA07	bna0423	46.8	BoC07_20728474_301	BoC07	20,728,474	AT2G47170		
BnA07	bna4786	47.1	BrA05_11057670_301			AT1G49100	AT1G49100	C
BnA07	bna0517	49.3	BoScaffold000284_16130_301			AT5G28340	AT5G28340	Q

BnA07	bna0547	50.1	BoC07_18776685_301	BoC07	18,776,685	AT5G28491	AT5G28491	Q
BnA07	Ol10-D03B	50.6				AT3G18000		
BnA07	bna4086	52.8	BoC03_33143147_301			AT4G00124		
BnA07	bna0036	52.8	BoScaffold000284_63570_300			AT5G35840	AT5G35840	S
BnA07	bna0035	52.8	BoScaffold000284_63051_301			AT5G35840	AT5G35840	S
BnA07	bna4817	55.3	BoScaffold000029_1654518_301			AT1G63680		
BnA07	bna3876	55.3	BoScaffold000029_1736580_301			AT3G02010		
BnA07	bna0351	55.3	BrA01_21633874_301			AT2G10250		
BnA07	bna3877	55.3	BoScaffold000029_1577084_301			AT5G40700	AT5G40700	S
BnA07	bna3878	55.3	BoScaffold000029_1537639_301			AT5G07070	AT5G07070	R
BnA07	bna3875	55.3	BoC06_13001402_300			AT5G05720	AT5G05720	R
BnA07	bna3149	57.4	BoC04_33944565_303			AT4G04316		
BnA07	bna0594	57.7	BoC07_15896409_301	BoC07	15,896,409	AT3G43715		
BnA07	bna0806	58	BoScaffold000401_176117_301			AT1G33813		
BnA07	bna0896	58.3	BoScaffold000401_119918_239			AT5G49580		
BnA07	BoGMS545	79.3	BrA07_277845_210	BrA07	277,845	AT2G20280	AT2G20280	H
BnA07	J0609	79.6						
BnA07	BGO114	79.6						
BnA07	bna5254	80.1	BrA07_287125_301	BrA07	287,125	AT2G14200	AT2G14200	H
BnA07	bna0323	80.1	BrA07_359031_301	BrA07	359,031	AT3G19340		
BnA07	bna2369	80.1	BrA07_367720_301	BrA07	367,720	AT3G42438		
BnA07	sR4047	83.8	BrA07_829294_270	BrA07	829,294	AT4G29510		
BnA07	bna2411	85.7	BrA07_923958_207	BrA07	923,958	AT2G18070	AT2G18070	H
BnA07	bna2410	86.5	BrA07_1197924_299	BrA07	1,197,924	AT5G62370		
BnA07	bna5003	87.8	BrA07_1300601_296	BrA07	1,300,601	AT2G18800	AT2G18800	H
BnA07	bna3045	87.8	BrA09_18277850_301			AT1G56020		

BnA07	bna3044	87.8	BrA07_1372394_300	BrA07	1,372,394	AT5G09920		
BnA07	bna3128	88.1	BrA07_1766618_273	BrA07	1,766,618	AT5G25750		
BnA07	bna3040	88.3	BrA10_11548392_301			AT2G17790	AT2G17790	H
BnA07	bna5056	88.5	BrA10_11839859_301			AT5G17350		
BnA07	bna3036	88.7	BrA10_11872043_301			AT2G17410	AT2G17410	H
BnA07	bna3043	88.9	BrA07_1603000_301	BrA07	1,603,000	AT5G48550		
BnA07	bna5888	88.9	BrA07_1734481_301	BrA07	1,734,481	AT1G07650		
BnA07	bna5002	88.9	BrA07_1595561_301	BrA07	1,595,561	AT2G19010	AT2G19010	H
BnA07	bna5264	88.9	BrA07_1766618_273	BrA07	1,766,618	AT5G25750		
BnA07	BoGMS1575	89.4						
BnA07	sR0282R	89.7						
BnA07	Na12-B02	91.1						
BnA07	bna5777	92.7	BrA10_3160721_301			AT2G15630	AT2G15630	H
BnA07	bna2377	93.1	BrA07_3065564_282	BrA07	3,065,564	AT2G04340		
BnA07	bna3037	94.7	BrA10_11839859_301			AT5G17350	AT5G17350	R
BnA07	bna5661	96	BrA10_3116354_268			ATCG00670		
BnA07	bna3029	96	BrA09_19718503_279			AT5G02030	AT5G02030	R
BnA07	bna3120	96	BrA10_3243515_298			AT1G31870		
BnA07	bna5669	96	BoScaffold000001_P2_2064625_267			AT5G26582		
BnA07	bna2378	96	BrA08_9537088_276			AT4G30210		
BnA07	bna2383	96.3	BrA07_3717065_301	BrA07	3,717,065	AT5G06380	AT5G06380	R
BnA07	bna2386	96.3	BrA07_3935049_301	BrA07	3,935,049	AT3G24530	AT3G24530	F
BnA07	bna2382	96.3	BrA07_3596136_301	BrA07	3,596,136	AT5G06690		
BnA07	bna2391	97.2	BrA07_4180352_301	BrA07	4,180,352	AT5G41120		
BnA07	bna2384	97.2	BrA07_3796323_302	BrA07	3,796,323	AT3G24630		
BnA07	bna2394	98.3	BrA07_4480120_291	BrA07	4,480,120	AT3G24230	AT3G24230	F

BnA07	Ra2-G08	98.8	BrA07_4761169_394	BrA07	4,761,169	AT2G32910		
BnA07	BEN236	98.8	BrA07_4675434_199	BrA07	4,675,434	AT4G21595		
BnA07	bna2396	99.3	BrA07_4911941_301	BrA07	4,911,941	AT2G16280		
BnA07	bna2400	99.3	BrA07_5089975_263	BrA07	5,089,975	AT3G23300	AT3G23300	F
BnA07	bna2390	100.1	BoC06_3357873_302			AT5G50080	AT5G50080	W
BnA07	bna2404	100.9	BrA07_5367229_301	BrA07	5,367,229	AT5G54390	AT5G54390	W
BnA07	bna4856	100.9	BrA01_10550339_299			AT5G09850		
BnA07	BrGMS38	101.8	BrA07_5615635_407	BrA07	5,615,635	AT2G03700		
BnA07	bna2416	103.3	BrA07_6248377_104	BrA07	6,248,377	AT3G52810		
BnA07	BEN45	103.6	BrA07_6280732_122	BrA07	6,280,732	AT1G29350	AT1G29350	B
BnA07	bna2418	104.5	BrA07_6360973_301	BrA07	6,360,973	AT3G20140		
BnA07	bna1324	106	BrA07_7009807_279	BrA07	7,009,807	AT1G27340	AT1G27340	B
BnA07	bna2432	107.1	BrA07_7562178_301	BrA07	7,562,178	AT2G11870		
BnA07	BGR20	107.8	BrA07_7572279_104	BrA07	7,572,279	AT3G33112		
BnA07	bna2433	109.1	BrA07_7610939_301	BrA07	7,610,939	AT1G27090	AT1G27090	B
BnA07	bna2434	109.1	BrA07_7635002_285	BrA07	7,635,002	AT3G23130		
BnA07	bna4857	109.1	BrA07_7713158_301	BrA07	7,713,158	AT2G39700		
BnA07	bna2435	109.6	BrA07_7752491_226	BrA07	7,752,491	AT1G27110	AT1G27110	B
BnA07	bna2436	109.6	BrA07_7874003_301	BrA07	7,874,003	AT1G27120	AT1G27120	B
BnA07	bna1477	109.9	BoC09_38401229_303			AT5G24210	AT5G24210	Q
BnA07	bna2452	110.8	BrA07_9030502_297	BrA07	9,030,502	AT4G10480		
BnA07	bna0287	112.1	BrA01_12936313_302			AT2G31020		
BnA07	CB10299	113.7	BrA07_9168262_154	BrA07	9,168,262	AT1G20470		
BnA07	bna0132	115	BrA07_9841219_301	BrA07	9,841,219	AT5G25752	AT5G25752	Q
BnA07	bna5173	115	BrA07_10076851_301	BrA07	10,076,851	AT5G26310	AT5G26310	Q
BnA07	bna2465	115	BrA07_10070123_301	BrA07	10,070,123	AT1G29730		

BnA07	bna2464	115	BrA07_10009675_301	BrA07	10,009,675	AT5G66770	AT5G66770	X
BnA07	bna2462	115	BrA07_9867651_305	BrA07	9,867,651	AT5G67050	AT5G67050	X
BnA07	bna2467	117.2	BoC06_647138_302			AT5G51200		
BnA07	BEN178	118						
BnA07	BEN288	118.8	BrA07_11178806_135	BrA07	11,178,806	AT2G28650	AT2G28650	I
BnA07	bna1305	119.9	BrA02_14309_301	BrA02	14,309	AT2G29040	AT2G29040	I
BnA07	bna1307	119.9	BrA02_76135_272	BrA02	76,135	AT2G36724	AT2G36724	J
BnA07	bna5521	122.4	BrA02_236832_302	BrA02	236,832	AT5G39000		
BnA07	bna1312	122.7	BrA02_303906_301	BrA02	303,906	AT2G39110	AT2G39110	J
BnA07	bna5368	122.7	BrA02_303906_301	BrA02	303,906	AT2G39110	AT2G39110	J
BnA07	bna5597	124.4	BrA04_9074350_301			AT5G40890	AT5G40890	S
BnA07	bna1315	125.1	BrA02_423955_211	BrA02	423,955	AT5G40340	AT5G40340	S
BnA07	bna5524	125.1	BrA02_613245_301	BrA02	613,245	AT5G41040	AT5G41040	S
BnA07	BEN254	126.2						
BnA07	bna5525	127	BrA02_770869_301	BrA02	770,869	AT5G41790	AT5G41790	S
BnA07	bna1320	127	BrA02_647559_280	BrA02	647,559	AT5G41220	AT5G41220	S
BnA07	bna2617	127	BrA02_6306888_301			AT5G22790		
BnA07	bna1322	127	BrA02_810600_301	BrA02	810,600	AT5G41890	AT5G41890	S
BnA07	bna5188	127.5	BrA07_11472403_301	BrA07	11,472,403	AT3G53235	AT3G53235	N
BnA07	bna2471	128.6	BrA07_11728297_301	BrA07	11,728,297	AT3G54440	AT3G54440	N
BnA07	bna2475	128.9	BrA07_11960515_301	BrA07	11,960,515	AT5G56950		
BnA07	bna2474	128.9	BrA07_11898064_287	BrA07	11,898,064	AT4G16590		
BnA07	bna2473	129.5	BrA07_11896650_302	BrA07	11,896,650	AT3G55120	AT3G55120	N
BnA07	bna2476	130.2	BrA07_12027872_306	BrA07	12,027,872	AT3G56100	AT3G56100	N
BnA07	sR7223	131.3	BrA07_12314707_413	BrA07	12,314,707	AT3G57160	AT3G57160	N
BnA07	Ol09-A06B	132.1						

BnA07	bna4662	134.3	BrA07_12361556_300	BrA07	12,361,556	AT3G57400	AT3G57400	N
BnA07	bna5682	134.7	BrA07_12511408_301	BrA07	12,511,408	AT3G20550		
BnA07	bna2483	134.7	BrA07_12411966_301	BrA07	12,411,966	AT3G57610	AT3G57610	N
BnA07	CB10439	135.1	BrA07_12579775_179	BrA07	12,579,775	AT3G58100	AT3G58100	N
BnA07	sNRA59	137.8	BrA07_13058693_125	BrA07	13,058,693			
BnA07	bna2489	139.4	BrA07_13156451_301	BrA07	13,156,451	AT3G60830	AT3G60830	N
BnA07	bna2504	139.7	BrA07_13654040_301	BrA07	13,654,040	AT5G24550		
BnA07	bna2503	139.7	BrA07_13642281_301	BrA07	13,642,281	AT1G59870		
BnA07	bna2500	139.7	BrA07_13513700_301	BrA07	13,513,700	AT1G54970		
BnA07	bna2498	139.7	BrA07_13347565_301	BrA07	13,347,565	AT3G61730	AT3G61730	N
BnA07	bna2496	139.7	BrA07_13283149_301	BrA07	13,283,149	AT3G61560	AT3G61560	N
BnA07	bna0085	139.7	BrA07_13171897_301	BrA07	13,171,897	AT3G61070	AT3G61070	N
BnA07	bna2502	140.7	BrA07_13568545_301	BrA07	13,568,545	AT3G62980	AT3G62980	N
BnA07	BnGMS147A	141.8	BrA07_13588712_232	BrA07	13,588,712	AT3G63010	AT3G63010	N
BnA07	bna5926	142.3	BrA07_13674921_300	BrA07	13,674,921	AT1G79840	AT1G79840	E
BnA07	bna5302	142.8	BrA07_13840515_301	BrA07	13,840,515	ATCG00180		
BnA07	bna5045	142.8	BrA07_14002506_301	BrA07	14,002,506	AT1G79610	AT1G79610	E
BnA07	bna2510	142.8	BrA07_14002506_301	BrA07	14,002,506	AT1G79610	AT1G79610	E
BnA07	bna2512	142.8	BrA07_14125225_301	BrA07	14,125,225	AT1G79030	AT1G79030	E
BnA07	bna4821	142.8	BrA07_14326373_302	BrA07	14,326,373	AT1G77920	AT1G77920	E
BnA07	bna0167	142.8	BrA07_14594194_301	BrA07	14,594,194	AT1G76878	AT1G76878	E
BnA07	bna4778	142.8	BrA07_14596135_301	BrA07	14,596,135	AT1G76878	AT1G76878	E
BnA07	bna0168	142.8	BrA07_14599861_301	BrA07	14,599,861	AT1G76870	AT1G76870	E
BnA07	bna2521	142.8	BrA07_14792117_299	BrA07	14,792,117	AT1G79750	AT1G79750	E
BnA07	bna2524	142.8	BrA07_14926716_301	BrA07	14,926,716	AT1G75330	AT1G75330	E
BnA07	bna2514	144.1	BrA07_14388103_301	BrA07	14,388,103	AT3G56760		

BnA07	BGR100	145.2	BrA07_14656668_112	BrA07	14,656,668	AT1G76590		
BnA07	BGR99A	145.2	BrA07_14701381_148	BrA07	14,701,381	AT3G26290		
BnA07	BGO156B	145.2	BrA07_15030696_149	BrA07	15,030,696	AT5G61495		
BnA07	bna5112	145.7	BoScaffold000343_161218_301			AT3G12020		
BnA07	bna2526	145.7	BrA07_15006833_301	BrA07	15,006,833	AT1G74970	AT1G74970	E
BnA07	bna2527	145.7	BrA07_15035219_301	BrA07	15,035,219	AT1G74910	AT1G74910	E
BnA07	bna0169	145.7	BrA07_15353942_297	BrA07	15,353,942	AT1G73290	AT1G73290	E
BnA07	bna5688	145.7	BrA07_15354338_301	BrA07	15,354,338	AT1G73290	AT1G73290	E
BnA07	bna0086	145.7	BrA07_13936337_300	BrA07	13,936,337	AT2G13410		
BnA07	bna0088	147.2	BrA07_15381136_300	BrA07	15,381,136	AT1G73177	AT1G73177	E
BnA07	bna0067	149.3	BrA07_16025144_275	BrA07	16,025,144	AT3G22190		
BnA07	bna2540	149.3	BrA07_16164923_302	BrA07	16,164,923	AT1G69580	AT1G69580	E
BnA07	bna2541	149.3	BrA07_16254256_301	BrA07	16,254,256	AT3G55540		
BnA07	Ol12-E03A	150.7	BrA07_16247167_117	BrA07	16,247,167			
BnA07	FITO035B	152.1						
BnA07	bna2547	153	BrA07_16967417_301	BrA07	16,967,417	AT1G65640	AT1G65640	E
BnA07	bna2549	153	BrA07_17047113_301	BrA07	17,047,113	AT1G65960	AT1G65960	E
BnA07	bna2551	153	BrA07_17212352_301	BrA07	17,212,352	AT2G29820		
BnA07	bna2555	153.9	BrA07_17418674_296	BrA07	17,418,674	AT1G67140	AT1G67140	E
BnA07	bna5047	154.6	BrA07_17839683_301	BrA07	17,839,683	AT3G25620		
BnA07	bna0018	155.3	BrA07_18115597_301	BrA07	18,115,597	AT5G24550		
BnA07	bna2562	155.6	BrA07_18088001_301	BrA07	18,088,001	AT5G59990		
BnA07	bna2559	155.6	BrA07_17846151_301	BrA07	17,846,151	AT1G68360	AT1G68360	E
BnA07	bna4955	155.9	BrA07_18261993_301	BrA07	18,261,993	AT1G69220	AT1G69220	E
BnA07	bna0047	155.9	BrA07_18127466_299	BrA07	18,127,466	AT1G68862	AT1G68862	E
BnA08	bna0381	0	BrA08_2513043_305			AT2G11730	AT2G11730	H

BnA08	bna2760	2.5	BrA08_914135_259			AT2G17530	AT2G17530	H
BnA08	BGR2	2.8	BrA08_1088421_148			AT5G55780		
BnA08	bna5304	3.1	BrA08_759127_301	BrA08	759,127	AT1G54000	AT1G54000	C
BnA08	bna2762	3.1	BrA08_814384_301	BrA08	814,384	AT1G53710	AT1G53710	C
BnA08	bna2743	5.8	BrA08_2516101_301	BrA08	2,516,101	AT1G49710	AT1G49710	C
BnA08	bna0378	6.2	BrA08_2500830_301	BrA08	2,500,830	AT1G47290	AT1G47290	C
BnA08	bna0374	6.9	BrA05_15205515_301			AT1G36840	AT1G36840	C
BnA08	bna5876	8.8	BrA08_2973687_301	BrA08	2,973,687	AT4G04290	AT4G04290	O
BnA08	bna5897	9.9	BrA08_2973687_301	BrA08	2,973,687	AT4G04290	AT4G04290	O
BnA08	BoGMS1375	12.4						
BnA08	bna5259	13.5	BrA08_3336709_303	BrA08	3,336,709	AT1G17410		
BnA08	BGR93	17.8	BrA08_5736292_113	BrA08	5,736,292	AT1G46192		
BnA08	bna0221	22.1	BrA08_7092588_301	BrA08	7,092,588	AT5G21222	AT5G21222	R
BnA08	BGR69	23.2	BrA08_6151459_136	BrA08	6,151,459	AT5G19300	AT5G19300	R
BnA08	BGR1A	23.2	BrA08_8946388_150	BrA08	8,946,388	AT5G35660		
BnA08	bna5706	24.6	BrA08_979684_89			AT5G14390	AT5G14390	R
BnA08	sS2331BB	26	BrA08_8588167_116	BrA08	8,588,167	AT5G06810	AT5G06810	R
BnA08	bna2756	27.8	BrA08_7182928_304	BrA08	7,182,928	AT3G53970		
BnA08	bna2753	27.8	BrA08_7410142_301	BrA08	7,410,142	AT5G05840	AT5G05840	R
BnA08	bna2759	27.8	BoScaffold000064_262868_307			AT2G15120	AT2G15120	H
BnA08	bna5411	27.8	BoScaffold000064_262868_307			AT2G15120	AT2G15120	H
BnA08	bna4864	28.3	BrA08_5366755_298	BrA08	5,366,755	AT2G14080	AT2G14080	H
BnA08	bna2710	28.7	BrA08_5664464_294	BrA08	5,664,464	AT1G71820		
BnA08	bna2925	28.8	BrA08_4781235_301	BrA08	4,781,235	AT5G28892	AT5G28892	Q
BnA08	bna2713	29	BrA08_5300196_301	BrA08	5,300,196	AT5G28916	AT5G28916	Q
BnA08	bna5052	29	BrA08_5490202_302	BrA08	5,490,202	AT2G39130		

BnA08	bna5705	29	BrA08_5660516_305	BrA08	5,660,516	AT4G31080		
BnA08	bna0319	30.2	BrA08_7064446_301	BrA08	7,064,446	AT1G63710		
BnA08	bna2752	30.2	BrA08_7444486_309	BrA08	7,444,486	AT1G34000	AT1G34000	B
BnA08	bna2704	30.2	BrA08_7717106_301	BrA08	7,717,106	AT5G50160		
BnA08	BGR61	31.3	BrA08_7614956_149	BrA08	7,614,956	AT1G33110	AT1G33110	B
BnA08	bna0291	31.9	BoC08_9845211_301			AT1G32700	AT1G32700	B
BnA08	bna1296	32.1	BrA08_7698295_301	BrA08	7,698,295	AT1G32361	AT1G32361	B
BnA08	bna0217	32.3	BrA08_7232063_301	BrA08	7,232,063	AT3G32917	AT3G32917	M
BnA08	bna2495	32.5	BoC04_17492930_266			AT3G43955	AT3G43955	M
BnA08	bna1525	33.1	BrScaffold000180_112061_233			AT4G05616		
BnA08	bna2596	35.4	BrA08_8479782_301	BrA08	8,479,782	AT5G33300	AT5G33300	S
BnA08	bna5303	35.6	BrA08_8495863_301	BrA08	8,495,863	AT5G61670		
BnA08	bna2599	35.6	BrA08_8721374_301	BrA08	8,721,374	AT5G44010		
BnA08	bna2598	35.6	BrA08_8589463_301	BrA08	8,589,463	AT5G26749		
BnA08	bna2707	35.9	BoC01_19271492_301			AT5G01849		
BnA08	bna1941	35.9	BrA08_8200184_317	BrA08	8,200,184	AT5G35700	AT5G35700	S
BnA08	BEN43	42.5	BrA08_10397240_227	BrA08	10,397,240	AT4G17950		
BnA08	BEN48	45	BrA08_10398587_201	BrA08	10,398,587	AT2G18300		
BnA08	FITO131	45.5						
BnA08	bna5221	46.9	BrA08_10186467_301	BrA08	10,186,467	AT5G55640	AT5G55640	W
BnA08	bna2694	46.9	BrA08_10186467_301	BrA08	10,186,467	AT5G55640	AT5G55640	W
BnA08	bna2699	48.4	BrA08_10711075_301	BrA08	10,711,075			
BnA08	BGR8	49.6	BrA08_11358558_145	BrA08	11,358,558			
BnA08	bna2686	51.4	BoScaffold000375_162328_301			AT4G25010	AT4G25010	U
BnA08	bna2687	51.4	BrA08_14729798_301	BrA08	14,729,798	AT4G25010	AT4G25010	U
BnA08	BRMS97	53.5	BrA08_14676090_194	BrA08	14,676,090	AT4G25410	AT4G25410	U

BnA08	sR3688	57.2	BrA08_16205351_255	BrA08	16,205,351	AT4G38550	AT4G38550	U
BnA08	BEN116	57.7	BrA08_16117222_181	BrA08	16,117,222	AT4G38900	AT4G38900	U
BnA08	bna2672	58.5	BrA08_16033023_308	BrA08	16,033,023	AT4G39200	AT4G39200	U
BnA08	bna2677	59.4	BrA08_15726752_301	BrA08	15,726,752	AT2G22480	AT2G22480	I
BnA08	bna5699	59.4	BrA08_15726752_301	BrA08	15,726,752	AT2G22480	AT2G22480	I
BnA08	bna4993	61	BrA08_16248783_301	BrA08	16,248,783	AT5G53200		
BnA08	bna2668	61.3	BrA08_16318859_301	BrA08	16,318,859	AT1G30460	AT1G30460	B
BnA08	bna4861	64.8	BrA08_16652551_302	BrA08	16,652,551	AT1G40099		
BnA08	BGO006	67.6	BrA08_16900709_113	BrA08	16,900,709	AT1G28400	AT1G28400	B
BnA08	BGO120B	68.1	BrA08_17125566_219	BrA08	17,125,566	AT5G37680		
BnA08	CALSSRA	68.9	BrA08_17620710_145	BrA08	17,620,710	AT1G26310	AT1G26310	B
BnA08	bna2652	69.9	BrA08_17642831_301	BrA08	17,642,831	AT1G26410	AT1G26410	B
BnA08	bna2653	69.9	BrA08_17586824_299	BrA08	17,586,824	AT1G26190	AT1G26190	B
BnA08	bna2650	70	BrA08_17858014_209	BrA08	17,858,014	AT5G20470		
BnA08	bna5404	70.1	BrA08_17858014_209	BrA08	17,858,014	AT5G20470		
BnA08	bna2654	70.2	BrA08_17579140_301	BrA08	17,579,140	AT1G26120	AT1G26120	B
BnA08	bna2655	70.3	BrA08_17535008_301	BrA08	17,535,008	AT1G25560	AT1G25560	B
BnA08	bna5462	70.3	BrA08_17473735_301	BrA08	17,473,735	AT1G25360	AT1G25360	B
BnA08	bna2649	70.3				AT1G50810		
BnA08	bna2660	71.1	BrA08_17192991_301	BrA08	17,192,991	AT5G26920		
BnA08	bna2661	71.1	BrA08_17158805_304	BrA08	17,158,805	AT4G04020		
BnA08	bna2659	71.1	BrA08_17297854_301	BrA08	17,297,854	AT1G24430	AT1G24430	B
BnA08	bna5856	71.4	BrA08_17050908_266	BrA08	17,050,908	AT3G25725		
BnA08	bna2662	71.4	BrA08_17000653_297	BrA08	17,000,653	AT5G25430		
BnA08	bna2621	78.5	BrA05_8223432_300			AT1G10480	AT1G10480	A
BnA08	bna2623	78.8	BrA05_8139038_304			AT1G10760	AT1G10760	A

BnA08	bna5218	78.8	BrA05_8139038_304			AT1G10760	AT1G10760	A
BnA08	bna2619	79.9	BrA05_8318584_302			AT3G15358		
BnA09	BEN332A	0	BrA09_936877_256	BrA09	936,877	AT4G00660		
BnA09	BEN50B	0.8	BrA09_936928_277	BrA09	936,928	AT1G26761	AT1G26761	B
BnA09	BEN50A	10.3	BrA09_936928_277	BrA09	936,928	AT1G26761	AT1G26761	B
BnA09	BEN332B	10.6	BrA09_936877_256	BrA09	936,877	AT4G00660	AT4G00660	O
BnA09	BN38A	11.4	BrA09_764538_156			AT4G01060	AT4G01060	O
BnA09	bna2964	20.4	BoC09_285052_304			AT4G01270	AT4G01270	O
BnA09	BEN55B	21.3	BrA09_2356689_162	BrA09	2,356,689	AT4G27500		
BnA09	FITO135A	21.9						
BnA09	BrGMS725A	30.9	BrA09_2302831_166	BrA09	2,302,831	AT5G48230		
BnA09	BnGMS133	43.3	BrA09_4198837_248	BrA09	4,198,837	AT5G66640		
BnA09	BnEMS820A	49.6						
BnA09	sR9447	53.6	BrA09_4281574_349	BrA09	4,281,574	AT3G01420		
BnA09	sN1988	54	BrA09_3907955_277	BrA09	3,907,955	AT2G13665	AT2G13665	H
BnA09	bna2954	54.9	BrScaffold000178_33738_301			AT2G14760	AT2G14760	H
BnA09	bna2566	55.1	BrA09_4683310_301	BrA09	4,683,310	AT4G12450		
BnA09	bna2953	55.3	BrScaffold000178_39098_249			AT2G14760	AT2G14760	H
BnA09	bna5309	55.3	BrA09_4905856_301	BrA09	4,905,856	AT2G18330	AT2G18330	H
BnA09	bna2952	55.3	BrA09_5298231_301	BrA09	5,298,231	AT2G18950	AT2G18950	H
BnA09	bna2955	55.8	BrA09_4357188_170	BrA09	4,357,188	AT5G67170		
BnA09	bna5176	57.2	BrA09_4940739_301	BrA09	4,940,739			
BnA09	bna5143	57.2	BrA09_5245915_301	BrA09	5,245,915	AT5G28675		
BnA09	bna0274	63.5	BrA09_6783993_301	BrA09	6,783,993	AT1G64610	AT1G64610	D
BnA09	bna2934	63.5	BrA09_6805269_300	BrA09	6,805,269	AT1G64510	AT1G64510	D
BnA09	bna0725	66.3	BoScaffold000215_564770_302			AT1G62430	AT1G62430	D

BnA09	BrGMS316	67.7	BrA09_9108578_310	BrA09	9,108,578	AT1G59740	AT1G59740	D
BnA09	bna2920	68.8	BrA09_9447053_301	BrA09	9,447,053	AT1G58340	AT1G58340	D
BnA09	bna5873	68.8	BrA09_9452991_253	BrA09	9,452,991	AT5G09520		
BnA09	bna0498	69.5	BoC08_1052043_301			AT3G04820		
BnA09	bna4685	70.2	BrA09_9735024_301	BrA09	9,735,024	AT4G04500		
BnA09	bna5419	70.7	BrA09_9724424_301	BrA09	9,724,424	AT5G11840		
BnA09	bna2916	71.3	BrA09_9845178_301	BrA09	9,845,178	AT1G56670	AT1G56670	D
BnA09	bna2902	71.6	BrA09_13737008_301	BrA09	13,737,008	AT1G19840		
BnA09	BEN284	73.1	BrA09_13822937_96	BrA09	13,822,937	AT5G43460	AT5G43460	V
BnA09	Na12-A01	73.4						
BnA09	BnGMS213B	74.8	BrA09_13917079_149	BrA09	13,917,079	AT1G67330		
BnA09	BnEMS973	77.3	BrA09_13642334_172	BrA09	13,642,334	AT4G38810		
BnA09	bna2905	79.5	BrA09_13278233_292	BrA09	13,278,233	AT3G51850		
BnA09	bna0275	80.1	BrA09_12962010_301			AT5G45340	AT5G45340	V
BnA09	bna2891	80.1	BrA09_16845654_301	BrA09	16,845,654	AT5G45430	AT5G45430	V
BnA09	bna5100	80.4	BrA09_16640161_301	BrA09	16,640,161	AT5G46070	AT5G46070	V
BnA09	bna2895	80.4	BrA09_16640161_301	BrA09	16,640,161	AT5G46070	AT5G46070	V
BnA09	bna2893	80.4	BrA09_16722763_301	BrA09	16,722,763	AT3G03790		
BnA09	bna2894	80.4	BrA09_16690925_301	BrA09	16,690,925	AT5G35495		
BnA09	bna5469	80.7	BrA09_17036026_301	BrA09	17,036,026	AT4G05120	AT4G05120	P
BnA09	bna0245	81	BrA08_9367918_302			AT5G13160		
BnA09	bna0244	81	BrA08_9420581_229			AT4G11280	AT4G11280	P
BnA09	bna0248	81	BrA08_9276658_302			AT3G61570		
BnA09	bna0246	81	BrA08_9326518_301			AT4G23820		
BnA09	bna2887	82.4	BrA07_5758348_284			AT4G11790	AT4G11790	P
BnA09	bna2313	83.5	BrA06_19739366_301			AT5G63820	AT5G63820	X

BnA09	bna5662	83.5	BrA06_19739366_301			AT5G63820	AT5G63820	X
BnA09	bna0328	85.4	BrA09_19111915_301	BrA09	19,111,915	AT3G43540		
BnA09	bna2882	85.4	BrA09_19145713_300	BrA09	19,145,713	AT5G52240		
BnA09	bna5417	85.4	BrA09_19152171_311	BrA09	19,152,171	AT5G27020		
BnA09	bna2872	85.4	BrA09_20115127_301	BrA09	20,115,127	AT1G32410	AT1G32410	B
BnA09	bna2180	86	BrA09_20620287_301	BrA09	20,620,287	AT3G60955		
BnA09	BGR60	87.4	BrA09_20556819_126	BrA09	20,556,819	AT1G31930	AT1G31930	B
BnA09	CB10022A	87.7						
BnA09	BGR42	87.7						
BnA09	sR9251J	88.2	BrA09_15477898_167			AT5G20830		
BnA09	BEN278A	88.7				AT5G47210		
BnA09	BrGMS726B	90.6						
BnA09	BGR43	95.4	BrA09_23392125_137	BrA09	23,392,125	AT1G26550	AT1G26550	B
BnA09	BGR23	99.5	BrA09_24532607_124	BrA09	24,532,607			
BnA09	BnGMS131	100	BrA09_24510113_135	BrA09	24,510,113	AT1G22850		
BnA09	BEN59	100.5	BrA09_23910646_178	BrA09	23,910,646	AT5G61440		
BnA09	BN9A	101						
BnA09	BnEMS1144	102.9	BrA09_23895488_281	BrA09	23,895,488	AT1G24120		
BnA09	bna2859	105.4	BrA09_23717141_301	BrA09	23,717,141	AT1G27045	AT1G27045	B
BnA09	bna2861	105.4	BrA09_23920888_299	BrA09	23,920,888	AT5G54420		
BnA09	bna2862	105.4	BrA09_23926596_301	BrA09	23,926,596			
BnA09	bna2865	105.7	BrA09_24105625_301	BrA09	24,105,625	AT1G23480	AT1G23480	B
BnA09	bna5289	109.3	BrA05_15805956_301			AT5G54470		
BnA09	bna4848	109.6	BrA09_24770365_301	BrA09	24,770,365	AT5G67180		
BnA09	bna2059	109.6	BoC05_17195187_301			AT1G21900	AT1G21900	B
BnA09	bna2603	110.3	BrA09_25224371_302	BrA09	25,224,371	AT5G56930		

BnA09	bna2852	110.3	BrA09_25503885_301	BrA09	25,503,885	AT3G51120	AT3G51120	N
BnA09	bna1598	111	BrA09_25004211_301	BrA09	25,004,211	AT1G12210		
BnA09	bna1596	111	BrA09_25131434_304	BrA09	25,131,434	AT5G51360		
BnA09	sR12777	112.6	BrA09_24782574_242	BrA09	24,782,574	AT3G57190		
BnA09	BGO179A	113.4	BrA09_25571083_231	BrA09	25,571,083	AT3G51240	AT3G51240	N
BnA09	BoGMS788	113.7						
BnA09	bna2825	118.6	BrA09_28241830_308	BrA09	28,241,830	AT3G55510	AT3G55510	N
BnA09	bna5141	118.6	BrA09_28241830_308	BrA09	28,241,830	AT3G55510	AT3G55510	N
BnA09	bna2820	119.7	BrA09_28706021_301	BrA09	28,706,021	AT3G57610	AT3G57610	N
BnA09	bna4867	119.7	BrA09_28910557_301	BrA09	28,910,557	AT5G48110		
BnA09	bna2815	120.4	BrA09_29101489_302	BrA09	29,101,489	AT3G58780	AT3G58780	N
BnA09	bna5468	120.4	BrA09_29310612_301	BrA09	29,310,612	AT2G43410		
BnA09	bna2810	120.4	BrA09_29348737_293	BrA09	29,348,737	AT5G15150		
BnA09	bna5306	122.3	BrA09_29496278_301	BrA09	29,496,278	AT3G59950	AT3G59950	N
BnA09	bna2809	122.5	BrA09_29454769_300	BrA09	29,454,769	AT1G47920		
BnA09	bna4828	122.5	BrA03_22478492_271			AT4G01530		
BnA09	bna2807	122.5	BrA09_29820627_301	BrA09	29,820,627	AT3G60850	AT3G60850	N
BnA09	Na14-B03	124.7						
BnA09	BEN84	126.1	BrA09_29791688_229	BrA09	29,791,688	AT1G80960		
BnA09	FITO018	130.1						
BnA09	BGO179B	143.9	BrA09_25571083_231			AT3G51240		
BnA10	Na12-E09	0	BrA10_5190092_263					
BnA10	Na12-D11	11						
BnA10	BnGMS249	15.9						
BnA10	BnGMS9	19.4	BrA10_1812746_129			AT5G41890		
BnA10	BoGMS1114	19.7						

BnA10	BGR66	19.7	BrA10_135760_150	BrA10	135,760	AT5G50170		
BnA10	sR6083	19.7	BrA10_648054_414	BrA10	648,054	AT1G47640		
BnA10	BGR71A	19.7						
BnA10	BGR64	19.7						
BnA10	BEN210	19.7	BrA10_901957_108	BrA10	901,957	AT1G07420	AT1G07420	A
BnA10	bna3129	20.5	BoC05_2322489_302			AT1G06720	AT1G06720	A
BnA10	sORH13B	21.3	BrA10_1629861_259	BrA10	1,629,861	AT1G06640	AT1G06640	A
BnA10	BoGMS197A	21.8	BrA10_6291918_156			AT5G24540		
BnA10	bna5773	23.6	BoScaffold000034_P1_1356708_56			AT5G35615		
BnA10	bna3127	24	BrA10_1513166_189	BrA10	1,513,166	AT1G06210	AT1G06210	A
BnA10	bna0209	24	BrScaffold000123_387624_301					
BnA10	bna0213	24.1	BrScaffold000123_306903_301			AT2G03570		
BnA10	bna0205	24.2	BrScaffold000291_24321_301			AT3G63250		
BnA10	bna3135	24.3	BrA10_284964_301			AT2G37370	AT2G37370	J
BnA10	bna0364	24.4	BrScaffold000123_221988_293			AT2G45060	AT2G45060	J
BnA10	bna4678	24.4	BrA10_3725683_301	BrA10	3,725,683	AT4G22540		
BnA10	bna3106	24.4	BrA01_18702013_301			AT2G04041		
BnA10	bna5520	24.4	BrScaffold000123_29982_302			AT5G22355	AT5G22355	Q
BnA10	bna4639	24.5	BrA02_22543533_301			AT1G47497		
BnA10	bna3104	24.6	BrA10_4969953_301	BrA10	4,969,953	AT5G28545	AT5G28545	Q
BnA10	bna5551	24.7	BrA02_22682772_310					
BnA10	bna1468	24.8	BrA02_22613210_254			AT5G28593	AT5G28593	Q
BnA10	bna3134	25.1	BrA10_379786_223			AT5G49880	AT5G49880	W
BnA10	bna1815	26	BrA10_6180843_301	BrA10	6,180,843	AT5G54300	AT5G54300	W
BnA10	bna1814	26.4	BrA10_6199138_301	BrA10	6,199,138	AT4G10590		
BnA10	bna2697	27.5	BrA10_6447037_301	BrA10	6,447,037	AT5G54730	AT5G54730	W

BnA10	bna3096	28.2	BoC09_28296469_301			AT4G23580		
BnA10	bna5771	28.2	BoC09_28296469_301			AT4G23580		
BnA10	bna3095	28.5	BrA10_7355531_301	BrA10	7,355,531	AT5G56360	AT5G56360	W
BnA10	bna0262	30.1	BrA04_12452903_297			AT5G12440		
BnA10	bna5857	30.4	BrScaffold000123_203127_301			AT1G52880		
BnA10	bna1469	30.7	BrA02_22693201_301			AT3G37820		
BnA10	bna2899	30.7	BrScaffold000369_8642_301					
BnA10	bna3087	30.7	BrA10_7809191_301	BrA10	7,809,191	AT5G57123	AT5G57123	W
BnA10	bna3086	34.1	BrA10_7906431_301	BrA10	7,906,431	AT5G57250	AT5G57250	W
BnA10	bna5770	34.1	BrA10_7906431_301	BrA10	7,906,431	AT5G57250	AT5G57250	W
BnA10	bna4972	34.2	BrA10_8285199_301	BrA10	8,285,199	AT5G58010	AT5G58010	W
BnA10	bna3091	34.3	BrA10_7607081_299	BrA10	7,607,081	AT2G05700		
BnA10	bna0797	34.4	BoC05_4605240_301			AT4G29900		
BnA10	bna3082	34.5	BrA10_8315472_301	BrA10	8,315,472	AT5G58110	AT5G58110	W
BnA10	bna5314	34.5	BrA10_8315472_301	BrA10	8,315,472	AT5G58110	AT5G58110	W
BnA10	BoGMS1199	36.4						
BnA10	BnEMS1185	36.9	BrA10_8369776_218	BrA10	8,369,776	AT5G58375	AT5G58375	W
BnA10	bna3079	38	BrA10_8387305_300	BrA10	8,387,305	AT5G58440	AT5G58440	W
BnA10	bna3074	38	BrA10_8564289_304	BrA10	8,564,289	AT5G58890	AT5G58890	W
BnA10	bna5704	38.7	BrScaffold000123_244680_301			AT1G67840		
BnA10	bna5936	38.7	BrA10_8766904_259	BrA10	8,766,904	AT5G57380		
BnA10	bna5889	38.7	BrA10_8766904_259	BrA10	8,766,904	AT5G57380		
BnA10	bna3069	39	BrA10_8857702_301	BrA10	8,857,702	AT5G59460	AT5G59460	W
BnA10	bna1597	39	BrA09_25072577_299			AT1G67105		
BnA10	bna5766	39.5	BrA10_9122134_302	BrA10	9,122,134	AT2G19910		
BnA10	bna5765	39.5	BrA10_9187027_301	BrA10	9,187,027	AT5G60210	AT5G60210	W

BnA10	bna3059	40.3	BrA10_9327235_301	BrA10	9,327,235	AT5G60550	AT5G60550	X
BnA10	bna3081	42.3	BoC09_26000052_293			AT5G66330	AT5G66330	X
BnA10	bna5544	49.7	BrA09_31314801_290			AT5G50920		
BnA10	bna5009	61.2	BrA10_13224157_301	BrA10	13,224,157	AT5G05840		
BnA10	bna5761	63.2	BrA10_13407917_302	BrA10	13,407,917	AT5G15550	AT5G15550	R
BnA10	bna5933	63.2	BrA10_13429404_301	BrA10	13,429,404	AT5G15500	AT5G15500	R
BnA10	bna4676	64.7	BoC09_33861997_259			AT5G14690	AT5G14690	R
BnA10	bna5019	64.7	BoC09_33861997_259			AT5G14690	AT5G14690	R
BnA10	BEN312	65.6				AT5G14580	AT5G14580	R
BnA10	BGO090	65.6	BrA10_13726214_212	BrA10	13,726,214	AT5G14460	AT5G14460	R
BnA10	bna3021	66.4	BrA10_13734397_303	BrA10	13,734,397	AT5G14420	AT5G14420	R
BnA10	bna3020	66.4	BrA10_13749676_299	BrA10	13,749,676	AT5G14370	AT5G14370	R
BnA10	bna5310	66.7	BrA10_13938715_302	BrA10	13,938,715	AT5G13670	AT5G13670	R
BnA10	bna3016	66.7	BrA10_13938715_302	BrA10	13,938,715	AT5G13670	AT5G13670	R
BnA10	bna3010	67.8	BrA10_14254838_301	BrA10	14,254,838	AT5G12480	AT5G12480	R
BnA10	bna5177	68.1	BrA10_14313425_301	BrA10	14,313,425	AT5G12400	AT5G12400	R
BnA10	bna3009	68.1	BrA10_14313425_301	BrA10	14,313,425	AT5G12400	AT5G12400	R
BnA10	bna5759	68.7	BrA10_14370230_311	BrA10	14,370,230	AT5G12210	AT5G12210	R
BnA10	bna3000	69	BrA10_14646372_302	BrA10	14,646,372	AT5G11420	AT5G11420	R
BnA10	bna5655	70.7	BrScaffold001837_576_301			AT2G22795		
BnA10	bna2270	70.7	BrScaffold001837_576_301			AT2G22795		
BnA10	bna2997	70.7	BrA10_14697676_302	BrA10	14,697,676	AT5G11260	AT5G11260	R
BnA10	bna5756	70.7	BrA10_14697676_302	BrA10	14,697,676	AT5G11260	AT5G11260	R
BnA10	bna2989	72.5	BrA10_15031960_301	BrA10	15,031,960	AT5G10200	AT5G10200	R
BnA10	bna2974	72.5	BoC09_37549777_301			AT5G09430	AT5G09430	R
BnA10	bna2973	72.5	BrA10_16433391_205	BrA10	16,433,391	AT2G06860		

BnA10	bna5749	72.5	BrA10_16433391_205	BrA10	16,433,391	AT2G06860		
BnA10	BnEMS1173	78	BrA10_15360864_275	BrA10	15,360,864	AT5G05750	AT5G05750	R
BnA10	BnEMS1156	78.3						
BnA10	bna5471	80.5	BrA10_15331714_302	BrA10	15,331,714	AT5G05670	AT5G05670	R
BnA10	bna0874	81	BrA10_16503144_298	BrA10	16,503,144	AT5G04990	AT5G04990	R
BnA10	bna3242	84.3	BrA01_9626079_301			AT4G27020		
BnA10	bna1805	84.6	BrA10_17449798_261	BrA10	17,449,798	AT5G02070	AT5G02070	R
BnA10	bna2968	84.6	BrA10_17501817_301	BrA10	17,501,817	AT5G01120	AT5G01120	R
BnA10	BnGMS175	86						
BnA10	BnEMS812	90.3						
BnC01	bna2225	0	BrA06_1840122_301			AT1G57590		
BnC01	bna2216	1.6	BrA06_1168297_301	BrA06	1,168,297	AT1G51860		
BnC01	bna5716	1.9	BrA06_26268826_297			AT4G38360	AT4G38360	U
BnC01	bna0385	1.9	BrA01_8207233_301			AT5G28635		
BnC01	bna2770	1.9	BrA06_26268826_297			AT4G38360	AT4G38360	U
BnC01	BGR16	2.9						
BnC01	BGO160	3.2						
BnC01	BEN391B	4.6						
BnC01	bna2219	7	BrA06_1480375_301	BrA06	1,480,375	AT1G50920	AT1G50920	C
BnC01	bna5093	7.4	BrA06_1480375_301	BrA06	1,480,375	AT1G50920	AT1G50920	C
BnC01	bna2220	8.5	BrA06_1529976_301	BrA06	1,529,976	AT1G50660	AT1G50660	C
BnC01	bna5645	8.5	BrA06_1613040_317	BrA06	1,613,040	AT1G50460	AT1G50460	C
BnC01	bna2222	8.5	BrA06_1613040_317	BrA06	1,613,040	AT1G50460	AT1G50460	C
BnC01	bna2228	9.4	BrA06_2261818_301	BrA06	2,261,818	AT2G42270		
BnC01	bna4787	9.7	BrA06_2542572_301	BrA06	2,542,572	AT2G04042		
BnC01	BoGMS1303	10.3						

BnC01	bna5649	13.5	BrA06_2902867_301	BrA06	2,902,867	AT1G08400	AT1G08400	A
BnC01	bna2234	15.2	BrA06_2756643_296			AT1G08065	AT1G08065	A
BnC01	sN9425	29.5	BoC01_11302996_367	BoC01	11,302,996	AT5G55920	AT5G55920	X
BnC01	bna5024	31.1	BoC01_11284149_301	BoC01	11,284,149	AT5G65630	AT5G65630	X
BnC01	bna5483	31.1	BoC01_11261704_301	BoC01	11,261,704	AT1G21200		
BnC01	bna3197	31.4	BoC01_11212130_301	BoC01	11,212,130	AT4G18960		
BnC01	bna3196	31.7	BoC06_35205331_302			AT3G60565		
BnC01	bna4694	31.7	BoC01_11284149_301	BoC01	11,284,149	AT5G65630	AT5G65630	X
BnC01	bna5060	31.7	BoC01_11218340_301	BoC01	11,218,340	AT4G24970	AT4G24970	U
BnC01	bna3198	32.2	BoC01_11204317_301	BoC01	11,204,317	AT4G24940	AT4G24940	U
BnC01	bna3199	32.7	BoC01_19550059_174			AT5G45095		
BnC01	bna4878	33.6	BoScaffold000024_2832841_302			AT3G50625		
BnC01	bna3206	33.6	BoC06_31921522_291			AT2G19840		
BnC01	bna3203	33.6	BoC01_11024274_301	BoC01	11,024,274	AT4G24440	AT4G24440	U
BnC01	bna3207	33.6	BoC01_10912713_298	BoC01	10,912,713	AT2G04490		
BnC01	bna5826	33.6	BoC01_10912713_298	BoC01	10,912,713	AT2G04490		
BnC01	bna5025	33.6	BoScaffold000024_2832965_301			AT5G19090		
BnC01	bna5825	33.6	BoC06_31921803_301					
BnC01	BEN6	36.4	BoC01_10755773_281	BoC01	10,755,773	AT4G24110	AT4G24110	U
BnC01	bna5109	38.1	BoC01_10695936_301	BoC01	10,695,936	AT4G24040	AT4G24040	U
BnC01	bna3211	38.1	BoC01_10693467_301	BoC01	10,693,467	AT4G24040	AT4G24040	U
BnC01	bna3212	38.1	BoC01_10671090_301	BoC01	10,671,090			
BnC01	bna3210	38.3	BoC01_10738704_301	BoC01	10,738,704	AT5G54510		
BnC01	bna4695	38.4	BoC09_34391901_189			AT4G07496		
BnC01	bna0605	48.5	BoScaffold000354_123402_295			AT1G43020		
BnC01	BEN348	55.3	BoC01_14211979_313	BoC01	14,211,979	AT5G54510		

BnC01	bna3249	56.2	BoC01_13821490_301	BoC01	13,821,490	AT3G03660		
BnC01	bna2009	56.2	BrA09_6090407_302			AT1G15280		
BnC01	bna0969	56.5	BoScaffold000127_1253028_301			AT4G28380		
BnC01	bna3760	57.8	BoScaffold000424_146746_302			AT4G13575		
BnC01	bna1032	58.4	BoC04_5903411_257			AT3G45253		
BnC01	bna0792	58.4	BoScaffold000127_609513_281			AT2G27760		
BnC01	bna3247	61	BoScaffold000338_130703_302			AT5G23800	AT5G23800	Q
BnC01	bna3244	61.5	BoC05_9312267_188			AT3G02960		
BnC01	bna4696	62	BoC06_12911807_301			AT5G27238	AT5G27238	Q
BnC01	bna3243	62	BrA04_2359134_301			AT5G28300	AT5G28300	Q
BnC01	bna3324	63.4	BoC01_14276456_301	BoC01	14,276,456	AT5G28487	AT5G28487	Q
BnC01	bna3320	63.4	BoScaffold000361_48863_301			AT5G29015	AT5G29015	Q
BnC01	bna0701	63.4	BoScaffold000237_639353_294			AT5G46960	AT5G46960	V
BnC01	bna0532	64.4	BoScaffold000337_4413_301			AT5G42930	AT5G42930	V
BnC01	bna5841	65.5	BoC01_14300995_301	BoC01	14,300,995	AT5G32022	AT5G32022	S
BnC01	bna3326	65.5	BoC01_14301103_301	BoC01	14,301,103	AT5G32022	AT5G32022	S
BnC01	bna3335	65.5	BoC01_16340725_301			AT5G50915		
BnC01	bna2979	67.8	BoC04_10825273_249			AT1G62510		
BnC01	bna0832	68.8	BoC01_17141760_301			AT4G02960		
BnC01	bna3327	69.3	BoC01_14319520_264	BoC01	14,319,520	AT5G34930	AT5G34930	S
BnC01	bna5437	69.3	BoC01_14403643_301	BoC01	14,403,643			
BnC01	bna0891	69.3	BoC01_14522842_301	BoC01	14,522,842	AT4G07760		
BnC01	bna3338	69.3	BoC01_14848536_301	BoC01	14,848,536	AT5G32431	AT5G32431	S
BnC01	bna0665	69.3	BoC01_16040336_301	BoC01	16,040,336	AT3G47740	AT3G47740	M
BnC01	bna3316	69.3	BoScaffold000361_123005_301			AT3G48740	AT3G48740	M
BnC01	bna3336	69.3	BoC08_10783461_307					

BnC01	bna3312	69.5	BoScaffold000361_162937_301			AT3G48740	AT3G48740	M
BnC01	bna3314	69.7	BoScaffold000361_140793_301			AT2G04035		
BnC01	bna3310	69.8	BoScaffold000361_218671_301			AT3G48810	AT3G48810	M
BnC01	bna3319	69.9	BoScaffold000361_58170_301			AT1G13300	AT1G13300	A
BnC01	bna3313	70	BoScaffold000361_152166_300			AT1G02930	AT1G02930	A
BnC01	bna3325	70.1	BoC01_14285456_301			AT4G20095		
BnC01	bna3330	70.2	BoC01_14397348_301			AT1G75680		
BnC01	BoGMS622	73.6						
BnC01	BoGMS1246	76.4						
BnC01	sS1867A	76.7						
BnC01	CB10258	77						
BnC01	Na12-C08	78.5				AT2G36080		
BnC01	bna5239	81.8	BoC01_21703665_246	BoC01	21,703,665	AT4G04920		
BnC01	bna0574	82.8	BoC01_29077523_285	BoC01	29,077,523	AT3G21290	AT3G21290	F
BnC01	BoGMS1399	85.9						
BnC01	bna3342	87.8	BoC01_31074192_301	BoC01	31,074,192	AT3G52200		
BnC01	bna3343	87.8	BoC01_31149562_301	BoC01	31,149,562	AT5G35935		
BnC01	sN3523R	88.3	BoC01_31581658_199	BoC01	31,581,658	AT3G18300	AT3G18300	F
BnC01	bna5879	88.8	BoC01_31086172_301	BoC01	31,086,172	AT2G22990		
BnC01	bna0941	88.8	BoC01_30797471_301	BoC01	30,797,471	AT1G01400		
BnC01	bna0553	89.1	BoC01_31930694_301	BoC01	31,930,694	AT1G23160		
BnC01	bna0988	91	BoC03_24421151_301			AT4G06555		
BnC01	bna3357	91.3	BoC03_50713788_301			AT4G04410		
BnC01	bna3350	92.4	BoC01_32625067_301	BoC01	32,625,067	AT3G16630	AT3G16630	F
BnC01	BoGMS561	98	BoC01_36186383_144	BoC01	36,186,383	AT3G12120	AT3G12120	F
BnC01	bna5517	100.6	BrA01_27667920_301			AT3G09230	AT3G09230	F

BnC01	BEN32B	101.6	BoC01_38372637_153	BoC01	38,372,637	AT3G07790		
BnC01	BEN410	101.9	BoC01_37885135_241	BoC01	37,885,135	AT3G08505	AT3G08505	F
BnC01	bna1286	103.4	BrA01_27902747_299			AT3G07590	AT3G07590	F
BnC01	bna0878	103.7	BoC07_23218661_179			AT2G16420		
BnC01	bna1290	103.7	BrA01_27968734_301			AT3G07280	AT3G07280	F
BnC01	bna3369	104	BrA01_28250786_301			AT3G06190	AT3G06190	F
BnC01	bna1298	104.8	BrA01_28260995_301			AT3G06070	AT3G06070	F
BnC01	bna1272	106.1	BrA01_25120365_93			AT3G02360	AT3G02360	F
BnC01	BRAS074A	108.2				AT4G08960		
BnC01	bna4598	121.8	BoScaffold000338_32239_301			AT2G47790		
BnC01	bna0648	123.8	BoScaffold000232_160965_301			AT1G43770		
BnC01	bna0225	144.9	BrA10_16110047_317			AT5G07830		
BnC01	bna4804	152.1	BrA01_4737322_301			AT4G18130		
BnC01	bna5274	168.7	BrA02_3965136_301			AT5G16490	AT5G16490	R
BnC01	bna1346	168.7	BrA02_3934592_289			AT5G16290	AT5G16290	R
BnC01	bna5366	176.9	BrA01_8122629_301			AT5G51060		
BnC01	bna0317	177.2	BrA01_8388907_301			AT5G52470		
BnC01	bna0318	177.2	BrA01_8395801_301			AT4G25650	AT4G25650	U
BnC01	bna1166	177.2	BrA01_8417463_207			AT4G25720	AT4G25720	U
BnC01	bna1180	178.4	BrA01_9269777_301			AT5G03890		
BnC01	bna1174	180.2	BrA01_10000380_303			AT1G26670		
BnC01	bna5500	180.5	BrA01_11951455_301			AT5G46820		
BnC01	bna1270	190.1	BrScaffold000164_308660_301			AT2G23950		
BnC02	BrGMS231B	0						
BnC02	BGO054	1.1						
BnC02	BrGMS231A	2.2						

BnC02	bna5066	2.8	BrA07_20511170_301	BrA07	20,511,170	AT1G75310	AT1G75310	E
BnC02	bna2588	2.8	BrA07_21918751_301	BrA07	21,918,751	AT3G25610		
BnC02	bna2584	3.5	BrA07_21529099_298	BrA07	21,529,099	AT1G77800	AT1G77800	E
BnC02	bna2585	3.8	BoC07_943422_239			AT1G78020	AT1G78020	E
BnC02	bna5215	3.8	BrA07_21637773_301	BrA07	21,637,773	AT4G09467		
BnC02	BEN425	29.8						
BnC02	MR52a	35.7	BoC02_6520290_134	BoC02	6,520,290	AT5G35680		
BnC02	bna0442	41.2	BoC02_8604252_305			AT3G11880		
BnC02	bna1358	41.5	BrA02_5592528_299			AT5G22760	AT5G22760	R
BnC02	bna1356	41.8	BrA02_5301560_301			AT5G21326	AT5G21326	R
BnC02	bna5529	42.1	BoC02_5955002_301			AT5G21140	AT5G21140	R
BnC02	bna1361	42.3	BrA02_5924413_148			AT5G52520		
BnC02	bna3388	42.6	BoScaffold000024_98819_301			AT3G29078		
BnC02	bna5532	42.6	BrA02_6228686_200			AT5G59290		
BnC02	bna1353	42.9	BrA02_4295817_301			AT5G17710	AT5G17710	R
BnC02	bna1352	42.9	BrA02_4240951_301			AT5G17530	AT5G17530	R
BnC02	bna1350	44	BrA02_4137450_301			AT5G17170	AT5G17170	R
BnC02	BnEMS1119	45.5	BoC02_7359570_209	BoC02	7,359,570	AT2G34820		
BnC02	BrGMS268	45.5	BoC02_7359632_296	BoC02	7,359,632	AT5G31087		
BnC02	BRAS083	45.5	BoC02_7413861_156	BoC02	7,413,861	AT5G17690	AT5G17690	R
BnC02	BEN134	46	BoC02_8463131_197	BoC02	8,463,131	AT3G01400		
BnC02	sN3761A	46.8	BoC02_9227135_179	BoC02	9,227,135	AT5G57510	AT5G57510	W
BnC02	BoGMS328	47.3						
BnC02	BEN396	48.1						
BnC02	bna0551	50.9	BoC02_10311145_301	BoC02	10,311,145	AT5G55520	AT5G55520	W
BnC02	bna4700	51	BoC02_11100426_239	BoC02	11,100,426	AT5G54510	AT5G54510	W

BnC02	bna3410	51.1	BoC02_10914216_301	BoC02	10,914,216	AT5G54730	AT5G54730	W
BnC02	bna3413	51.2	BoC02_10734477_301	BoC02	10,734,477			
BnC02	bna3409	51.2	BoC01_28896212_301			AT2G05915		
BnC02	bna3392	51.2	BoC02_11087706_230	BoC02	11,087,706	AT5G22440		
BnC02	bna4963	51.4	BoScaffold000024_58713_299			AT4G05145		
BnC02	bna3384	51.6	BoScaffold000344_317376_304			AT5G54300	AT5G54300	W
BnC02	bna3385	51.6	BoScaffold000344_348629_302			AT5G54280	AT5G54280	W
BnC02	bna3383	51.6	BrA10_7152347_268			AT2G21590		
BnC02	bna3386	52.3	BoScaffold000024_11794_301			AT5G54250	AT5G54250	W
BnC02	bna3380	52.5	BoC06_26158817_188			AT4G17800		
BnC02	bna3375	52.7	BoC06_26016799_299			AT3G10290		
BnC02	bna3379	52.7	BoC06_26117133_301			AT5G53770	AT5G53770	W
BnC02	bna3371	52.7	BoC06_25893091_286			AT5G53550	AT5G53550	W
BnC02	bna3387	52.7	BoScaffold000024_78841_304			AT5G11660		
BnC02	bna3389	52.7	BoScaffold000024_319346_301			AT1G62940		
BnC02	bna2583	54.8	BrA07_21437273_301			AT1G77390		
BnC02	bna3517	56.5	BoScaffold000001_P2_387136_301			AT3G27330		
BnC02	bna3522	56.7	BoScaffold000001_P2_243108_301			AT5G52410	AT5G52410	W
BnC02	bna3525	56.9	BrA02_8653202_301			AT5G52470	AT5G52470	W
BnC02	bna4983	57.9	BoScaffold000001_P2_168438_302			AT3G05415		
BnC02	bna3524	58	BoScaffold000001_P2_214096_301			AT5G24030		
BnC02	bna3519	58	BoScaffold000001_P2_358314_297			AT1G71480		
BnC02	bna3520	58.8	BoScaffold000001_P2_334963_301			AT2G21410		
BnC02	bna4791	60.1	BoScaffold000001_P2_714220_301			AT5G51812	AT5G51812	W
BnC02	bna5942	61.3	BoScaffold000001_P2_669898_302			AT1G32530		
BnC02	bna3505	62.2	BoScaffold000001_P2_1178512_301			AT2G37670		

BnC02	bna3508	62.2	BoScaffold000001_P2_629586_302			AT3G33197		
BnC02	bna3510	62.2	BoScaffold000001_P2_541721_301			AT5G52050	AT5G52050	W
BnC02	Ol13-G05	63.8						
BnC02	BEN281A	65.6	BoC02_11649330_219	BoC02	11,649,330	AT1G67730	AT1G67730	E
BnC02	Na12-H09	66.8				AT4G21850		
BnC02	Ol09-A06A	67.7	BoC02_26800345_89					
BnC02	bna3862	70.1	BoC02_16785367_301	BoC02	16,785,367	AT1G73650	AT1G73650	E
BnC02	bna3863	70.1	BoC02_16849058_301	BoC02	16,849,058	AT5G59720		
BnC02	bna3864	70.1	BoC02_17022146_301	BoC02	17,022,146	AT2G04520		
BnC02	bna1423	70.4	BrA02_13167839_301			AT1G73680	AT1G73680	E
BnC02	BGO146	95.4				AT1G15460		
BnC02	bna1432	97.2	BrA02_14903333_301			AT3G31442		
BnC02	bna1223	97.6	BoC05_1773758_301			AT4G30872		
BnC02	bna5051	98.6	BrA05_9241979_297			AT5G62370		
BnC02	bna0619	99.4	BrA05_6407217_297			AT2G05040		
BnC02	bna0309	100.5	BoC02_24685579_301	BoC02	24,685,579	AT4G02260		
BnC02	bna5545	100.5	BoC02_24759788_303	BoC02	24,759,788	AT2G22780		
BnC02	bna1439	100.5	BrA02_15879592_265	BrA02	15,879,592	AT1G80370	AT1G80370	E
BnC02	bna1440	100.5	BrA02_16588127_301	BrA02	16,588,127	AT4G39400		
BnC02	bna2134	101.2	BrA05_9166879_314			AT4G02460	AT4G02460	O
BnC02	bna2136	101.5	BrA05_9054410_303			AT4G02600	AT4G02600	O
BnC02	bna2135	101.8	BrA05_9080490_301			AT3G05210		
BnC02	bna4836	102.8	BrA02_19401694_301	BrA02	19,401,694	AT5G37150		
BnC02	bna5209	102.8	BrA02_19573717_301	BrA02	19,573,717	AT5G47420	AT5G47420	V
BnC02	bna2268	102.8	BrA02_19573717_301	BrA02	19,573,717	AT5G47420	AT5G47420	V
BnC02	BGR58	103.3	BoC02_33628790_105			AT5G47500	AT5G47500	V

BnC02	bna5035	104.1	BrA02_19401939_301	BrA02	19,401,939	AT5G19610		
BnC02	bna1455	104.1	BrA02_19646538_297	BrA02	19,646,538	AT5G47530	AT5G47530	V
BnC02	bna5627	105.5	BoC02_33293315_168			AT4G35590		
BnC02	BEN296	108.9				AT5G09790		
BnC02	bna5548	109.9	BoC02_36430470_302			AT3G49650		
BnC02	bna1472	111.5	BrA02_22157503_301	BrA02	22,157,503	AT2G02670	AT2G02670	K
BnC02	bna5552	111.5	BrA02_22157503_301	BrA02	22,157,503	AT2G02670	AT2G02670	K
BnC02	bna1473	111.5	BrA02_22357115_301	BrA02	22,357,115	AT3G26750		
BnC02	bna5554	112.4	BrA02_23362692_299	BrA02	23,362,692	AT5G46590		
BnC02	bna5556	114.5	BrA02_24201837_301	BrA02	24,201,837	AT2G14365	AT2G14365	K
BnC02	bna1490	114.8	BrA02_24269285_301	BrA02	24,269,285	AT5G27970	AT5G27970	Q
BnC02	bna1492	114.8	BrA02_24456622_301	BrA02	24,456,622	AT5G27690	AT5G27690	Q
BnC02	bna1494	114.8	BrA02_24580861_304	BrA02	24,580,861	AT5G26820	AT5G26820	Q
BnC02	bna1088	115.9	BrA02_25898411_314	BrA02	25,898,411	AT5G61540		
BnC02	bna1087	116.3	BrA02_26014141_254	BrA02	26,014,141	AT5G07300		
BnC02	bna1086	117	BrA02_26034636_301	BrA02	26,034,636	AT5G61960	AT5G61960	X
BnC02	bna0664	117.5	BrA02_26034248_221	BrA02	26,034,248	AT5G61960	AT5G61960	X
BnC02	bna0053	117.5	BrA02_26164634_301	BrA02	26,164,634	AT4G07425		
BnC02	BRMS-026	121.5						
BnC02	BEN14	124.9				AT5G62440	AT5G62440	X
BnC02	bna1080	127.1	BrA02_27246329_302	BrA02	27,246,329	AT5G27010		
BnC03	BnEMS824	0						
BnC03	BnEMS1171	0.8	BoC03_56984210_644	BoC03	56,984,210	AT1G54080		
BnC03	bna0488	1.9	BoC03_56831583_301	BoC03	56,831,583	AT5G35920		
BnC03	BGR89	3.8	BoC03_55727445_158	BoC03	55,727,445			
BnC03	BoGMS1153	4.6						

BnC03	bna0578	37	BoC06_40640551_301			AT2G34070	AT2G34070	J
BnC03	BoGMS576	39.8						
BnC03	Na12-E02B	41.6				AT1G17310		
BnC03	BRAS120	44	BoC03_8598996_193	BoC03	8,598,996	ATCG00480		
BnC03	BoGMS693	44						
BnC03	bna0584	49.5	BoC03_8917659_299	BoC03	8,917,659	AT5G45000		
BnC03	bna0109	54.5	BoC03_9403365_301	BoC03	9,403,365	AT2G36910	AT2G36910	J
BnC03	Ol10-E05	56.5	BoC03_9847453_95	BoC03	9,847,453	AT2G37840	AT2G37840	J
BnC03	bna3238	59	BoC03_10671466_301	BoC03	10,671,466	AT2G39060	AT2G39060	J
BnC03	bna5831	59	BoC03_10700524_301	BoC03	10,700,524	AT4G11375		
BnC03	bna3235	61.7	BoC03_10826083_300	BoC03	10,826,083	AT2G39310	AT2G39310	J
BnC03	bna3234	62	BoC03_10837267_301	BoC03	10,837,267	AT4G38640		
BnC03	bna5830	62	BoC03_10837868_301	BoC03	10,837,868	AT4G38170		
BnC03	bna3232	62	BoC03_10876501_299	BoC03	10,876,501	AT2G39620	AT2G39620	J
BnC03	bna0420	62.8	BoScaffold000417_72597_301			AT2G42330	AT2G42330	J
BnC03	bna0815	63.1	BoC03_13119401_301	BoC03	13,119,401	AT2G40360	AT2G40360	J
BnC03	bna3642	64.1	BoC03_14248590_301	BoC03	14,248,590	AT2G25290		
BnC03	CB10057	65.8	BoC03_14249960_197	BoC03	14,249,960	AT3G43250		
BnC03	BRAS051A	65.8						
BnC03	sNRA56	65.8	BoC03_14457833_251	BoC03	14,457,833	AT5G47770		
BnC03	bna4752	68	BoC03_14715730_223	BoC03	14,715,730	AT5G27350	AT5G27350	Q
BnC03	BRAS087A	70.2	BoC03_16290437_171	BoC03	16,290,437	AT5G16220		
BnC03	BRAS005	70.2	BoC03_16290458_196	BoC03	16,290,458	AT5G25370	AT5G25370	Q
BnC03	bna1672	71.3	BrA03_12402499_301			AT4G09160	AT4G09160	P
BnC03	bna3691	71.3	BoC03_16510429_301	BoC03	16,510,429	AT4G11160	AT4G11160	P
BnC03	bna3695	71.5	BoScaffold000445_115904_301			AT4G11660	AT4G11660	P

BnC03	bna3693	71.7	BoScaffold000445_36202_301			AT1G08290		
BnC03	bna3641	71.9	BoC04_4765967_301					
BnC03	bna3694	72.1	BoScaffold000445_93975_301			AT1G31340		
BnC03	bna3696	72.2	BoScaffold000445_129823_301			AT1G65900		
BnC03	bna3697	72.3	BoC03_17189721_301	BoC03	17,189,721	AT4G11910	AT4G11910	P
BnC03	bna3698	72.3	BoC03_17198073_301	BoC03	17,198,073	AT5G48990		
BnC03	bna0810	73.1	BoC03_17384693_301	BoC03	17,384,693	AT4G12460	AT4G12460	P
BnC03	bna0527	73.4	BoC03_18059121_301	BoC03	18,059,121	AT1G80960		
BnC03	BnGMS153	77.3	BoC03_19715716_184	BoC03	19,715,716	AT3G01790	AT3G01790	F
BnC03	bna3665	78.4	BoC03_19898731_291	BoC03	19,898,731	AT3G01070	AT3G01070	F
BnC03	FITO007.2	82.1						
BnC03	bna3725	84.6	BoScaffold000040_247857_301			AT3G09285	AT3G09285	F
BnC03	bna4359	84.6	BoScaffold000040_345382_301			AT1G42655		
BnC03	bna3728	84.9	BoScaffold000040_77369_301			AT5G67550		
BnC03	bna3726	84.9	BoScaffold000040_222077_301			AT3G09200	AT3G09200	F
BnC03	bna3753	87.8	BoScaffold000366_83782_301			AT5G26150		
BnC03	BrGMS556	91.4	BoC03_21360451_159	BoC03	21,360,451	AT3G13810	AT3G13810	F
BnC03	BEN78	96.7	BoC03_26376496_139	BoC03	26,376,496	AT1G04985		
BnC03	BnGMS584A	99.2	BoC03_27306726_219	BoC03	27,306,726	AT5G52545		
BnC03	bna0400	100.1	BoC03_27365297_301	BoC03	27,365,297	AT2G12462		
BnC03	Na10-E02C	102.9						
BnC03	Na10-E02B	106.4						
BnC03	Na12-G05	107.8						
BnC03	bna0842	109.4	BoC07_28315359_306			AT5G32470		
BnC03	bna0989	109.4	BoC07_27998777_301			AT4G06497		
BnC03	bna0711	109.4	BoScaffold000389_119417_304			AT5G05130		

BnC03	bna0545	109.9	BoC03_29474486_301	BoC03	29,474,486	AT1G66960		
BnC03	bna0960	110.4	BoC03_29818084_301	BoC03	29,818,084	AT2G18960		
BnC04	bna4772	0	BoScaffold000479_44720_309			AT1G13600		
BnC04	bna0741	2	BoC04_34927_301	BoC04	34,927	AT3G49290		
BnC04	bna3880	2	BoScaffold000492_36021_301			AT5G56780		
BnC04	bna3881	2	BoScaffold000492_80177_301			AT2G46260	AT2G46260	J
BnC04	BRAS072	2.7						
BnC04	BnEMS634	2.7	BoC04_28573_164	BoC04	28,573	AT2G40430	AT2G40430	J
BnC04	BEN337	2.7	BoC04_28531_179	BoC04	28,531	AT2G40430	AT2G40430	J
BnC04	BEN266	2.7	BoC04_121523_205	BoC04	121,523	AT2G40540	AT2G40540	J
BnC04	BoGMS876	7						
BnC04	sS2277	7	BoC04_2065086_215	BoC04	2,065,086	AT1G65590		
BnC04	BGO140	7.3	BoC04_2985740_184	BoC04	2,985,740	AT3G20640		
BnC04	bna5435	9.8	BoC03_21757372_301			AT3G01720		
BnC04	bna3898	11.4	BoScaffold000152_P2_118199_301			AT2G39950	AT2G39950	J
BnC04	bna3893	11.7	BoC04_7053518_301	BoC04	7,053,518	AT2G39660	AT2G39660	J
BnC04	bna3896	12	BoC04_7283093_289	BoC04	7,283,093	AT4G32880		
BnC04	bna3895	12.4	BoC04_7107888_301	BoC04	7,107,888	AT5G36905		
BnC04	bna3885	13.3	BoC04_6681467_301	BoC04	6,681,467	AT3G50730		
BnC04	bna3890	14	BoC04_6765723_301	BoC04	6,765,723	AT2G39370	AT2G39370	J
BnC04	bna3884	14	BoC04_6662671_301	BoC04	6,662,671	AT2G47040		
BnC04	bna3889	14.3	BoC04_6755490_300	BoC04	6,755,490	AT2G39350	AT2G39350	J
BnC04	bna3886	14.6	BoC04_6692801_301	BoC04	6,692,801	AT2G39220	AT2G39220	J
BnC04	bna3899	17	BoC04_4943209_301	BoC04	4,943,209	AT2G37500	AT2G37500	J
BnC04	bna3901	18.4	BoC04_4853652_301	BoC04	4,853,652	AT2G37370	AT2G37370	J
BnC04	BGR83	19.9	BoC04_4863957_119	BoC04	4,863,957	AT5G13740		

BnC04	bna3902	21.2	BoC04_4832311_301	BoC04	4,832,311	AT2G37280	AT2G37280	J
BnC04	bna3905	21.2	BoC04_4687761_301	BoC04	4,687,761	AT4G32610		
BnC04	bna3903	22.2	BoC04_4811011_301	BoC04	4,811,011	AT2G37260	AT2G37260	J
BnC04	bna3904	22.2	BoC04_4788675_301	BoC04	4,788,675	AT2G37210	AT2G37210	J
BnC04	bna3910	22.2	BoC04_4503191_230	BoC04	4,503,191	AT2G36620	AT2G36620	J
BnC04	bna2154	26	BrA05_5043649_301			AT2G35130	AT2G35130	J
BnC04	bna5967	30.2	BoC04_20851795_301	BoC04	20,851,795	AT3G33004		
BnC04	BGO141	31.8	BoC04_20034307_263	BoC04	20,034,307	AT4G02400		
BnC04	BoGMS560	32.6	BoC04_34176660_276			AT3G09160		
BnC04	bna0695	38	BoScaffold000024_972317_297			AT3G61440		
BnC04	bna0938	38	BoScaffold000024_1207662_301			AT5G23430		
BnC04	BEN269	42.8	BoC04_19590343_175	BoC04	19,590,343	AT3G47450		
BnC04	bna3769	43.7	BoScaffold000498_71526_216					
BnC04	bna4214	48.8	BoScaffold000637_7130_226			AT4G02733		
BnC04	bna4000	63.9	BoC01_25489245_300					
BnC04	bna4001	64.2	BoC01_25461049_301			AT2G25070	AT2G25070	I
BnC04	bna4718	64.2	BoC04_29837685_301	BoC04	29,837,685	AT3G45673		
BnC04	BEN239	66.4	BoC04_30860617_210	BoC04	30,860,617	AT2G27860	AT2G27860	I
BnC04	BGO041	66.4	BoC04_30860620_207	BoC04	30,860,620	AT2G27860	AT2G27860	I
BnC04	bna4135	66.7	BoC04_31011812_301	BoC04	31,011,812	AT2G28160	AT2G28160	I
BnC04	CB10196	67.2	BoC04_33057690_247	BoC04	33,057,690	AT1G43960		
BnC04	bna4022	69.8	BoC04_36389682_301	BoC04	36,389,682	AT4G10570		
BnC04	bna4019	71.4	BoC04_36163611_301	BoC04	36,163,611	AT4G11360		
BnC04	bna4009	71.7	BoC03_20863405_301			AT3G46460		
BnC04	BEN30A	73.6	BoC04_36133841_239	BoC04	36,133,841	AT2G35540	AT2G35540	J
BnC04	bna4032	77.4	BoC04_37567682_301	BoC04	37,567,682	AT2G38060	AT2G38060	J

BnC04	bna4033	78.8	BoC04_37601346_301	BoC04	37,601,346	AT5G61960		
BnC04	BEN330A	83.2	BoC04_39009491_117	BoC04	39,009,491	AT2G40765		
BnC04	bna3654	90.9	BoC04_39447484_301	BoC04	39,447,484	AT2G42760		
BnC04	BEN387	98.4	BoC04_39865802_148	BoC04	39,865,802	AT5G08560		
BnC04	BnEMS439B	101.9				AT5G48230		
BnC04	BEN216	103.3	BoC04_40450483_103	BoC04	40,450,483	AT3G05100		
BnC04	bna4038	104.4	BoC04_37790278_301			AT2G38470	AT2G38470	J
BnC04	bna5613	104.4	BoC04_37966586_296			AT2G38910	AT2G38910	J
BnC04	Ol10-B01C	104.7	BoC04_39600479_169	BoC04	39,600,479	AT5G63440		
BnC04	BEN260	104.7	BoC04_38004702_204	BoC04	38,004,702	AT2G39170	AT2G39170	J
BnC04	bna4042	105.9	BoC04_38020170_301	BoC04	38,020,170	AT2G39200	AT2G39200	J
BnC04	bna4046	106.4	BoC04_38310490_301	BoC04	38,310,490	AT2G39810	AT2G39810	J
BnC04	BEN28	107.2	BoC04_38625818_244	BoC04	38,625,818	AT2G40400	AT2G40400	J
BnC04	bna2013	107.5	BoC04_39112360_301	BoC04	39,112,360	AT2G40930	AT2G40930	J
BnC04	BEN420	107.8						
BnC04	Na12-E05A	107.8	BoC04_39335346_159	BoC04	39,335,346	AT2G41510	AT2G41510	J
BnC04	bna3657	108.9	BoC08_15576714_301			AT2G39190		
BnC04	bna5200	109	BrA04_17055673_303			AT3G47870		
BnC04	bna5088	109.1	BrA04_18941625_301			AT4G38270		
BnC04	bna3659	109.2	BoC04_39408421_301	BoC04	39,408,421	AT2G41820	AT2G41820	J
BnC04	bna4048	109.2	BoC04_39634651_301	BoC04	39,634,651	AT2G43140	AT2G43140	J
BnC04	bna2011	109.2	BrA04_17309194_302			AT5G16350		
BnC04	bna4051	109.2	BoC04_39944696_258	BoC04	39,944,696	AT2G43890	AT2G43890	J
BnC04	bna4054	109.2	BoC04_40220984_301	BoC04	40,220,984	AT2G44940	AT2G44940	J
BnC04	bna5901	111.4	BoScaffold000383_71931_301			AT2G26695		
BnC04	BEN313A	113.8	BoC04_40800903_129	BoC04	40,800,903	AT4G16610		

BnC04	BGO044	114.4	BoC04_40264415_217	BoC04	40,264,415	AT2G45120	AT2G45120	J
BnC04	bna5089	115.4	BrA04_18552225_301			AT2G45420	AT2G45420	J
BnC04	bna2022	115.4	BrA04_18552225_301			AT2G45420	AT2G45420	J
BnC04	bna4050	115.4	BoC04_39746095_301	BoC04	39,746,095	AT4G28110		
BnC04	bna4044	115.7	BoC04_38084313_301	BoC04	38,084,313	AT3G55090		
BnC04	BEN376	119.7	BoC04_40441062_146	BoC04	40,441,062	AT2G45700	AT2G45700	J
BnC04	Na12-E05B	132.1	BoC04_39335346_159	BoC04	39,335,346	AT2G41510		
BnC04	Ol10-B01A	132.5	BoC04_39600479_169	BoC04	39,600,479	AT5G63440		
BnC04	bna1518	137.9	BrA04_18370211_301			AT2G44650	AT2G44650	J
BnC04	Ol10-B01B	138.8	BoC04_39600479_169	BoC04	39,600,479	AT5G63440		
BnC04	bna1519	140.3	BrA04_18248518_214	BrA04	18,248,518	AT2G44060	AT2G44060	J
BnC04	bna1522	141.7	BrA04_18097253_288	BrA04	18,097,253	AT2G43430	AT2G43430	J
BnC04	bna1524	141.7	BrA04_17922813_301	BrA04	17,922,813	AT2G43010	AT2G43010	J
BnC04	bna2015	142.6	BrA04_17628226_85	BrA04	17,628,226	AT2G41830	AT2G41830	J
BnC04	bna5246	143	BrA04_17577550_301	BrA04	17,577,550	AT2G41560	AT2G41560	J
BnC04	bna2004	145.3	BrA04_16799529_301	BrA04	16,799,529	AT2G39470	AT2G39470	J
BnC04	bna1988	146.7	BrA04_15519727_301	BrA04	15,519,727	AT2G35820	AT2G35820	J
BnC04	bna1987	147.5	BrA04_15411549_301	BrA04	15,411,549	AT2G35600	AT2G35600	J
BnC04	BEN30B	148.2	BoC04_36133841_239			AT2G35540	AT2G35540	J
BnC04	bna4017	149.7	BoC04_35968371_301			AT2G34960	AT2G34960	J
BnC04	bna4013	150.4	BoC04_35723468_301			AT3G24982		
BnC04	bna1975	151.2	BrA04_14885528_301	BrA04	14,885,528	AT2G33480	AT2G33480	J
BnC04	bna1816	151.6	BrA04_14899563_301	BrA04	14,899,563			
BnC04	bna1973	151.6	BrA04_14639857_301	BrA04	14,639,857	AT2G32730	AT2G32730	J
BnC04	bna5286	152.6	BrA04_14588034_301	BrA04	14,588,034	AT2G32660	AT2G32660	J
BnC04	bna1964	153.1	BrA04_13541330_301	BrA04	13,541,330	AT2G30362	AT2G30362	J

BnC04	bna1965	153.3	BrA04_13571365_291	BrA04	13,571,365	AT2G30440	AT2G30440	J
BnC04	bna5383	153.5	BrA04_13490597_299	BrA04	13,490,597	AT2G30230	AT2G30230	J
BnC04	bna1963	153.7	BrA04_13435626_298	BrA04	13,435,626	AT2G26720		
BnC04	bna1985	153.7	BrA04_13293395_301	BrA04	13,293,395	AT2G29930	AT2G29930	J
BnC04	bna1984	153.7	BoC04_32547519_302			AT2G29580	AT2G29580	J
BnC04	bna1983	153.7	BoC04_32509105_285			AT2G29390	AT2G29390	J
BnC04	BrGMS426	160.8	BoC04_37779242_205			AT2G38380		
BnC04	Ol11-B05	170.8						
BnC04	BGR55	174.1				AT5G23050		
BnC04	bna1956	175.8	BrA04_12982228_301	BrA04	12,982,228	AT3G57620		
BnC04	bna5039	176.4	BrA04_12759591_267	BrA04	12,759,591	AT2G28507		
BnC04	bna1958	176.4	BrA04_12775106_301	BrA04	12,775,106	AT5G07572		
BnC04	bna4796	180.2	BoScaffold000008_P1_233001_301			AT4G14920		
BnC04	bna5542	180.5	BrA03_24870135_301			AT2G15700		
BnC04	bna1433	180.5	BrA03_24869598_301			AT4G05133		
BnC04	bna1923	180.8	BrA04_10941413_232			AT4G15680		
BnC04	bna5959	181.1	BoScaffold000008_P1_232056_301			AT1G31630		
BnC04	BnEMS843	183.9	BoC04_30578742_303			AT2G27290	AT2G27290	I
BnC04	bna1930	184.6	BrA04_12051217_211	BrA04	12,051,217	AT5G28295		
BnC04	bna5915	184.9	BrA04_11901963_301	BrA04	11,901,963	AT3G22190		
BnC04	bna1929	184.9	BrA04_11857893_301	BrA04	11,857,893	AT2G26980	AT2G26980	I
BnC04	bna5601	185.2	BrA04_11641375_301	BrA04	11,641,375	AT1G65630		
BnC04	bna1927	185.8	BrA04_11512229_301	BrA04	11,512,229	AT2G25660	AT2G25660	I
BnC04	bna1925	185.8	BrA04_11436981_301	BrA04	11,436,981	AT5G43030		
BnC04	BGO078	187.3				AT2G24070	AT2G24070	I
BnC04	sN2025	188.7				AT2G12960		

BnC04	bna1913	193	BrA04_9930221_301	BrA04	9,930,221	AT2G22740	AT2G22740	I
BnC04	bna5598	193	BrA04_9866051_301	BrA04	9,866,051	AT4G09875		
BnC04	bna0006	193	BrA04_7342568_290			AT2G22540	AT2G22540	I
BnC04	bna1912	193.4	BrA04_9866051_301	BrA04	9,866,051	AT4G09875		
BnC04	bna0161	193.8	BoScaffold000053_1407745_301			AT2G22540	AT2G22540	I
BnC04	bna0128	193.8	BrA04_9829689_301	BrA04	9,829,689	AT2G22540	AT2G22540	I
BnC04	bna5199	194.3	BrA04_9543819_301	BrA04	9,543,819	AT3G23085		
BnC04	bna1906	195.1	BoScaffold000301_484897_216			AT4G02480		
BnC04	bna1905	195.1	BrA04_8788332_264	BrA04	8,788,332	AT3G18165		
BnC04	bna1910	196.7	BrA04_9531877_301	BrA04	9,531,877	AT2G22125	AT2G22125	I
BnC04	BEN229	197.5	BoC04_29308814_232			AT2G21230	AT2G21230	I
BnC04	BEN95	198.3				AT4G07960		
BnC04	BGR71B	199.7						
BnC04	bna1903	200.2	BrA04_8431313_305	BrA04	8,431,313	AT5G41612		
BnC05	BGR49	0	BoC05_27137863_144			AT5G11800		
BnC05	bna0559	1.3	BoC05_26882248_301			AT3G17040		
BnC05	bna3300	1.3	BoScaffold000237_761486_300			AT4G08092		
BnC05	bna4794	4.9	BoC04_13352367_301			AT5G35643		
BnC05	bna0448	6.5	BoScaffold000450_12080_301			AT3G51380		
BnC05	BnGMS208	7.4						
BnC05	BnGMS198	7.4						
BnC05	sS2131	8.3	BoC05_8854465_174	BoC05	8,854,465	AT1G78560		
BnC05	BoGMS319	16.6	BoC05_7007360_227	BoC05	7,007,360	AT1G62870		
BnC05	bna4117	19.7	BoC05_13932622_288	BoC05	13,932,622	AT3G18130		
BnC05	bna4125	20.2	BoC05_29708580_318			AT2G07210		
BnC05	bna4124	20.2	BoC05_15224000_302	BoC05	15,224,000	AT1G19835	AT1G19835	A

BnC05	bna4127	20.2	BoC05_15339888_301	BoC05	15,339,888	AT1G19840	AT1G19840	A
BnC05	bna4288	22.1	BoC05_16496431_301	BoC05	16,496,431	AT3G29180		
BnC05	bna4289	22.1	BoC05_16473116_245	BoC05	16,473,116	AT5G01250		
BnC05	bna0194	22.4	BoC09_19095369_301			AT2G11800		
BnC05	Na12-C01	23.2	BoC05_16975819_85	BoC05	16,975,819			
BnC05	bna4065	28.1	BoScaffold000333_229916_301			AT4G00020		
BnC05	bna4072	28.4	BoC05_25517337_305	BoC05	25,517,337	AT1G10640	AT1G10640	A
BnC05	bna4073	28.7	BoC05_25556828_227	BoC05	25,556,828	AT5G51470		
BnC05	bna4075	29	BoC05_25710318_302	BoC05	25,710,318	AT1G10417	AT1G10417	A
BnC05	bna4076	29.6	BoC05_25894318_301	BoC05	25,894,318	AT5G09320		
BnC05	bna4079	29.6	BoC05_26000822_301	BoC05	26,000,822	AT1G09900	AT1G09900	A
BnC05	bna3414	29.6	BoC02_10603202_301			AT3G26670		
BnC05	bna4078	29.6	BoC05_25948119_305	BoC05	25,948,119	AT1G59560		
BnC05	bna4080	30.1	BoC05_26021073_301	BoC05	26,021,073	AT1G09860	AT1G09860	A
BnC05	BEN81	32	BoC05_25942418_171	BoC05	25,942,418	AT4G32900		
BnC05	bna4082	35.7	BoC05_3205578_301	BoC05	3,205,578	AT1G08750	AT1G08750	A
BnC05	BGO151	37.9	BoC05_3149393_257	BoC05	3,149,393	AT1G08650	AT1G08650	A
BnC05	BEN363	37.9	BoC05_3150464_236	BoC05	3,150,464	AT1G08650	AT1G08650	A
BnC05	bna4083	39.3	BoC05_3151125_301	BoC05	3,151,125	AT1G08650	AT1G08650	A
BnC05	bna4084	41	BoC05_2997517_301	BoC05	2,997,517	AT1G08260	AT1G08260	A
BnC05	bna4085	42	BoScaffold000087_P1_63592_301			AT2G43710		
BnC05	bna4090	46.5	BoC05_2553972_301	BoC05	2,553,972	AT4G18880		
BnC05	bna4089	46.5	BoC05_2576267_301	BoC05	2,576,267			
BnC05	bna4093	47.2	BoC05_2440345_300	BoC05	2,440,345	AT1G07110	AT1G07110	A
BnC05	bna4094	47.2	BoC05_2416377_281	BoC05	2,416,377	AT2G39435		
BnC05	sORH13A	49						

BnC05	bna4056	55.3	BoC05_395352_301	BoC05	395,352	AT5G48670		
BnC05	bna4057	55.3	BoC05_283542_301	BoC05	283,542	AT1G01180	AT1G01180	A
BnC05	BEN140	86.8						
BnC05	bna0623	87.6	BoC09_9601331_299			AT3G54910	AT3G54910	W
BnC05	BoGMS1561	88.1						
BnC05	FITO114	92.1						
BnC05	bna3301	92.4	BoScaffold000249_566822_301			AT5G07322		
BnC05	bna4610	95.8	BoScaffold000364_152113_301			AT5G60660	AT5G60660	W
BnC05	BEN285	117.4				AT4G33320		
BnC05	BrGMS387	119.7						
BnC05	bna2197	121.3	BrA05_1492477_301	BrA05	1,492,477	AT4G09190		
BnC05	bna2192	122.1	BrA05_2109698_321	BrA05	2,109,698	AT2G44560		
BnC05	bna0023	124	BrA05_2534365_297	BrA05	2,534,365	AT5G57510	AT5G57510	W
BnC05	bna2182	124	BrA05_2604626_246	BrA05	2,604,626	AT2G45910	AT2G45910	J
BnC05	bna5640	124.6	BrA05_2402280_301	BrA05	2,402,280	AT2G45340	AT2G45340	J
BnC05	bna2187	124.6	BrA05_2287965_301	BrA05	2,287,965	AT2G45010	AT2G45010	J
BnC05	bna2181	125.9	BrA05_2718163_301	BrA05	2,718,163	AT3G26460		
BnC05	bna0371	125.9	BrA05_2752010_301	BrA05	2,752,010	AT2G40230	AT2G40230	J
BnC05	bna2179	128.8	BrA05_3043046_300	BrA05	3,043,046	AT2G39670	AT2G39670	J
BnC05	bna2178	129.7	BrA05_3135216_162	BrA05	3,135,216			
BnC05	bna5639	130	BrA05_3324028_301	BrA05	3,324,028	AT2G38800	AT2G38800	J
BnC05	bna2174	130	BrA05_3550306_265	BrA05	3,550,306	AT4G38070		
BnC05	bna2173	130	BrA05_3610261_301	BrA05	3,610,261	AT2G38170	AT2G38170	J
BnC05	bna5453	130	BrA05_3615531_301	BrA05	3,615,531	AT2G38150	AT2G38150	J
BnC05	bna5205	130.3	BrA05_3782985_299	BrA05	3,782,985	AT2G46370		
BnC05	BnEMS1036	131.9				AT5G25070		

BnC05	BEN211	133.8				AT2G34980	AT2G34980	J
BnC05	BRAS063	135.7				AT5G19550	AT5G19550	R
BnC05	BrGMS351	136				AT5G19550	AT5G19550	R
BnC05	BrGMS124	142.9	BoC05_19788361_158			AT2G21630		
BnC05	BGR51	142.9				AT1G76780	AT1G76780	E
BnC05	BnEMS1072	143.4				AT1G74450	AT1G74450	E
BnC05	Na12-E01	143.4						
BnC05	bna2120	144.5	BrA05_9646616_251	BrA05	9,646,616	AT1G67550	AT1G67550	E
BnC05	bna2069	145	BoC05_8041058_241			AT3G26560	AT3G26560	L
BnC05	bna1165	145.3	BoC09_24054236_321			AT2G14790		
BnC05	bna5130	145.3	BrA05_11714048_301	BrA05	11,714,048	AT3G30110	AT3G30110	L
BnC05	bna5870	145.6	BrA05_12033088_301	BrA05	12,033,088	AT3G32093	AT3G32093	L
BnC05	bna2091	145.6	BrA05_15013435_301			AT3G42886	AT3G42886	L
BnC05	bna5625	145.6	BrA05_15086181_301			AT3G31720	AT3G31720	L
BnC05	bna2138	146.9	BrA05_7855910_301			AT2G29510		
BnC05	bna5629	146.9	BrA05_9900847_301			AT3G44630	AT3G44630	L
BnC05	bna2122	146.9	BrA01_11993737_299			AT1G04150	AT1G04150	A
BnC05	bna2075	148	BrA05_14029064_301			AT1G12330	AT1G12330	A
BnC05	bna2066	148.1	BrA05_15356777_304			AT3G53480		
BnC05	bna2060	148.2	BrScaffold004022_427_301					
BnC05	bna5620	148.2	BrA05_13538777_301	BrA05	13,538,777			
BnC05	bna2113	148.3	BrA05_12011855_301	BrA05	12,011,855	AT1G12870	AT1G12870	A
BnC05	bna2076	148.4	BrA05_14040757_301	BrA05	14,040,757	AT1G34130	AT1G34130	B
BnC05	BGR50	149.8	BoC05_13100763_143			AT1G32500	AT1G32500	B
BnC05	Na10-E02A	149.8				AT3G54090		
BnC05	bna2085	150.9	BrA05_14595356_301	BrA05	14,595,356	AT1G31930	AT1G31930	B

BnC05	bna2087	150.9	BrA05_14742565_301	BrA05	14,742,565	AT1G31772	AT1G31772	B
BnC05	bna1120	150.9	BrScaffold000280_11257_302			AT3G17480	AT3G17480	F
BnC05	bna2118	151.4	BrA05_10292928_301			AT3G17250	AT3G17250	F
BnC05	bna0354	151.9	BrA05_12838818_301	BrA05	12,838,818	AT3G10730	AT3G10730	F
BnC05	bna2061	151.9	BrA05_13227028_301			AT1G38131	AT1G38131	C
BnC05	bna2083	151.9	BrA05_14444029_301			AT1G46840	AT1G46840	C
BnC05	bna2114	151.9	BrA05_11984544_301	BrA05	11,984,544	AT1G49340	AT1G49340	C
BnC05	bna2115	152.7	BrA05_11980424_301	BrA05	11,980,424	AT1G49340	AT1G49340	C
BnC05	bna2116	152.7	BrA05_11945153_301	BrA05	11,945,153	AT1G49480	AT1G49480	C
BnC05	bna2133	153.2	BrA05_11840091_301	BrA05	11,840,091	AT1G50460	AT1G50460	C
BnC05	bna2751	153.2	BrA08_1336839_301			AT4G36791	AT4G36791	U
BnC05	bna0342	154.2	BrA05_13301566_206	BrA05	13,301,566	AT4G24230	AT4G24230	U
BnC05	bna5012	154.2	BrA05_14740842_277	BrA05	14,740,842	AT4G18940	AT4G18940	U
BnC05	bna2111	154.2	BrA05_12052721_301	BrA05	12,052,721	AT4G10115		
BnC05	bna2105	154.2	BrA05_12426734_301	BrA05	12,426,734			
BnC05	bna0252	154.2	BrA02_22725096_301			AT5G43810		
BnC05	bna0253	154.2	BrScaffold000201_41824_301			AT5G66840		
BnC05	bna2101	154.2	BrA05_12888421_299	BrA05	12,888,421	AT5G12085	AT5G12085	R
BnC05	bna2071	154.2	BrA05_13880226_296	BrA05	13,880,226	AT5G16100	AT5G16100	R
BnC05	bna2131	154.6	BrA05_11718716_303			AT5G26890	AT5G26890	Q
BnC05	bna2077	155.1	BrA05_14155289_301	BrA05	14,155,289	AT5G28810	AT5G28810	Q
BnC05	bna5292	156.1	BrA05_12930401_301			AT5G28916	AT5G28916	Q
BnC05	bna2104	156.6	BrA05_12457820_301			AT5G32513	AT5G32513	Q
BnC05	bna2082	156.6	BrA05_14435311_301	BrA05	14,435,311	AT5G35356	AT5G35356	S
BnC05	bna2056	157	BrA05_16844353_301	BrA05	16,844,353	AT5G35413	AT5G35413	S
BnC05	bna2107	157.6	BrA05_12307753_301			AT5G36275	AT5G36275	S

BnC05	bna0668	159.2	BoC03_9427817_301			AT2G11630		
BnC05	bna2128	161.9	BrA05_11607194_301			AT1G50840		
BnC05	bna2124	162.2	BrA05_11106780_301			AT5G38580	AT5G38580	S
BnC05	bna2057	163.1	BrA05_16835720_305	BrA05	16,835,720	AT3G22415	AT3G22415	F
BnC05	bna2375	164	BrA05_17832691_301	BrA05	17,832,691	AT2G06910		
BnC05	bna4801	164.4	BrA05_17757971_229	BrA05	17,757,971	AT3G19508	AT3G19508	F
BnC05	bna5017	165.1	BrA05_17913610_301	BrA05	17,913,610	AT3G19260	AT3G19260	F
BnC05	bna4729	165.8	BoScaffold000509_10901_301			AT3G25750		
BnC05	bna5619	165.8	BrA05_18458288_258	BrA05	18,458,288	AT3G18370	AT3G18370	F
BnC05	bna2049	166.4	BrA05_18654178_301	BrA05	18,654,178	AT3G18140	AT3G18140	F
BnC05	bna4798	166.4	BoC05_23892687_300			AT3G18090	AT3G18090	F
BnC05	bna2866	166.4	BrA09_6357318_261			AT1G60890		
BnC05	BGR88B	168.6						
BnC05	bna0769	170	BoC05_23817122_301			AT1G60180		
BnC05	bna2048	170	BrA05_18778891_301	BrA05	18,778,891	AT5G25475		
BnC05	bna1162	170.6	BoScaffold000106_777496_318			AT3G29260		
BnC05	bna2045	172.4	BrA05_21047756_279	BrA05	21,047,756	AT3G13160	AT3G13160	F
BnC05	bna5617	173	BrA05_21102199_301	BrA05	21,102,199	AT3G12980	AT3G12980	F
BnC05	bna2043	173	BrA05_21481144_301	BrA05	21,481,144	AT3G11910	AT3G11910	F
BnC05	bna2042	173.8	BrA05_21631356_301	BrA05	21,631,356	AT3G11550	AT3G11550	F
BnC05	bna2044	174.3	BrA05_21107331_301	BrA05	21,107,331	AT1G34300	AT1G34300	B
BnC05	bna5011	175.4	BrA05_21646834_301	BrA05	21,646,834	AT1G51840		
BnC05	BGR70	175.4				AT1G26680	AT1G26680	B
BnC05	bna0554	186.9	BoScaffold000091_219034_231			AT5G55940		
BnC06	BoGMS204	0	BoC07_1194234_388			AT2G14843		
BnC06	BEN374B	8.4	BoC07_1042174_213			AT1G77840	AT1G77840	E

BnC06	bna5460	21.2	BoC07_3687528_301			AT1G73400	AT1G73400	E
BnC06	bna2572	21.2	BoC07_3687528_301			AT1G73400	AT1G73400	E
BnC06	bna2568	21.7	BrA07_19154025_301			AT1G71210	AT1G71210	E
BnC06	BEN335	27.6	BoC07_30944062_119			AT1G79160		
BnC06	bna5689	27.6	BrA07_21961835_303	BrA07	21,961,835	AT1G78590	AT1G78590	E
BnC06	bna2589	27.6	BrA07_22034312_301	BrA07	22,034,312	AT1G78882	AT1G78882	E
BnC06	bna2592	27.6	BrA07_22193291_301	BrA07	22,193,291	AT1G79280	AT1G79280	E
BnC06	bna2593	27.6	BoC07_302532_301	BoC07	302,532	AT1G79320	AT1G79320	E
BnC06	bna2587	27.9	BoC07_771518_295	BoC07	771,518	AT1G78290	AT1G78290	E
BnC06	BnEMS994	30.7	BoC07_1198475_240	BoC07	1,198,475	AT1G21610		
BnC06	BEN374A	32.6	BoC07_1042174_213	BoC07	1,042,174	AT1G77840	AT1G77840	E
BnC06	bna2577	33.5	BrA07_20501932_294			AT1G75280	AT1G75280	E
BnC06	bna1012	33.5	BoC07_2451947_301	BoC07	2,451,947	AT1G75080	AT1G75080	E
BnC06	BnEMS82B	34.4	BoC07_2965046_191	BoC07	2,965,046	AT1G74970	AT1G74970	E
BnC06	bna4145	35.7	BoC07_3320152_301	BoC07	3,320,152	AT1G74110	AT1G74110	E
BnC06	bna4144	35.7	BoC07_3469180_301	BoC07	3,469,180	AT2G10555		
BnC06	BnEMS1124	36.5	BoC07_3642978_334	BoC07	3,642,978	AT1G73540	AT1G73540	E
BnC06	bna2570	37	BrA07_19430864_301			AT1G71940	AT1G71940	E
BnC06	bna5863	37.1	BoC01_10128342_270			AT5G53530		
BnC06	BEN16	37.2	BoC07_4905589_121	BoC07	4,905,589	AT1G70470	AT1G70470	E
BnC06	bna5410	37.5	BoC01_25355578_299			AT5G22930		
BnC06	bna4115	37.8	BoC07_4914531_301	BoC07	4,914,531	AT1G70430	AT1G70430	E
BnC06	bna4793	37.8	BoC07_7504465_225	BoC07	7,504,465	AT5G08390		
BnC06	BGO055	38.1	BoC07_7490142_210	BoC07	7,490,142	AT1G69480	AT1G69480	E
BnC06	bna4106	38.4	BoC07_7558675_301	BoC07	7,558,675	AT1G69320	AT1G69320	E
BnC06	bna5135	42.1	BrA07_18992968_301			AT2G27610		

BnC06	bna4116	44	BoC07_4899883_227			AT4G02235		
BnC06	bna4141	44.8	BoC07_3712871_301			AT1G03800		
BnC06	bna0395	45.3	BrA07_21174257_235			AT5G16060		
BnC06	bna2747	45.3	BoC01_25355779_301			AT4G10690		
BnC06	bna0104	46	BoC07_7706128_301	BoC07	7,706,128	AT1G69120	AT1G69120	E
BnC06	bna4901	46.4	BoC07_7711919_297	BoC07	7,711,919	AT2G28560		
BnC06	BEN327	46.9	BoC07_8457556_255	BoC07	8,457,556	AT1G68330	AT1G68330	E
BnC06	BEN271	46.9	BoC07_7556363_123	BoC07	7,556,363	AT3G30214	AT3G30214	L
BnC06	BEN341A	47.2	BoC07_8535142_132	BoC07	8,535,142	AT5G62700		
BnC06	Ap1c5pr	47.5						
BnC06	BGO056	48.6	BoC07_7556363_123	BoC07	7,556,363	AT3G30214	AT3G30214	L
BnC06	bna5046	53.5	BrA07_17488642_228			AT5G13190		
BnC06	bna4754	54	BoC07_2693719_260			AT1G44800		
BnC06	bna3900	54	BoC04_4881367_301			AT3G15350		
BnC06	bna0669	55.9	BoC07_8913646_301	BoC07	8,913,646	AT1G66410	AT1G66410	E
BnC06	BEN228	57	BoC07_2509216_254			AT1G67510	AT1G67510	E
BnC06	BoGMS1186	57						
BnC06	BEN341B	57.3	BoC07_8535142_132	BoC07	8,535,142	AT5G62700		
BnC06	bna0821	58.4	BoScaffold000336_264017_301			AT1G66880		
BnC06	bna2558	58.4	BrA07_17705637_301	BrA07	17,705,637	AT1G67830	AT1G67830	E
BnC06	bna2554	58.4	BrA07_17412606_301	BrA07	17,412,606	AT1G67140	AT1G67140	E
BnC06	bna2553	59.5	BrA07_17388386_301	BrA07	17,388,386	AT4G32610		
BnC06	bna0114	61.7	BrA07_16767833_301	BrA07	16,767,833	AT1G67420	AT1G67420	E
BnC06	bna0175	62.2	BrA07_16759938_301	BrA07	16,759,938	AT3G26940		
BnC06	bna0470	62.2	BoC07_9423305_301			AT1G65060		
BnC06	bna4782	62.7	BrA07_16758082_301	BrA07	16,758,082	AT1G67490	AT1G67490	E

BnC06	bna0113	62.7	BrA07_16757315_301	BrA07	16,757,315	AT1G67490	AT1G67490	E
BnC06	bna0046	62.7	BrA07_16755651_301	BrA07	16,755,651	AT1G67490	AT1G67490	E
BnC06	bna0174	62.9	BrA07_16728857_301	BrA07	16,728,857	AT1G67550	AT1G67550	E
BnC06	bna4952	63.2	BrA07_16722238_285	BrA07	16,722,238	AT5G32702		
BnC06	bna0017	63.2	BrA07_16701988_301	BrA07	16,701,988	AT1G67710	AT1G67710	E
BnC06	bna0034	63.2	BrA07_16695280_301	BrA07	16,695,280	AT5G24350		
BnC06	bna2545	63.2	BrA07_16686772_302	BrA07	16,686,772	AT4G01430		
BnC06	bna2544	63.4	BrA07_16677737_301	BrA07	16,677,737	AT4G06488		
BnC06	bna0133	63.8	BrA07_16558893_301	BrA07	16,558,893	AT1G68020	AT1G68020	E
BnC06	bna4781	64.2	BrA07_16100952_301	BrA07	16,100,952	AT5G13360		
BnC06	bna0033	65.5	BrA07_16100357_301	BrA07	16,100,357	AT4G25090		
BnC06	bna4780	65.5	BrA07_16099540_301	BrA07	16,099,540	AT3G24640		
BnC06	bna0016	65.5	BrA07_16095439_301	BrA07	16,095,439	AT5G24070	AT5G24070	Q
BnC06	bna2539	65.5	BrA07_16095439_301	BrA07	16,095,439	AT5G24070	AT5G24070	Q
BnC06	bna0171	66.7	BrA07_16089881_301	BrA07	16,089,881	AT5G38975		
BnC06	bna0068	67.1	BrA07_16070666_301	BrA07	16,070,666	AT5G24280	AT5G24280	Q
BnC06	bna0015	67.5	BrA07_16040715_301	BrA07	16,040,715	AT5G43710		
BnC06	bna2538	67.5	BrA07_15931600_301	BrA07	15,931,600	AT1G70610	AT1G70610	E
BnC06	bna2528	67.9	BrA07_15058904_301	BrA07	15,058,904	AT1G74720	AT1G74720	E
BnC06	bna2525	67.9	BrA07_14970124_301	BrA07	14,970,124	AT1G75200	AT1G75200	E
BnC06	FITO035A	68.3						
BnC06	BnEMS82A	69.1	BoC07_32508597_207			AT1G74970	AT1G74970	E
BnC06	BnGMS205	70.9						
BnC06	bna0148	73	BoC07_35677613_301	BoC07	35,677,613	AT1G69120	AT1G69120	E
BnC06	bna0009	73	BoC07_35675639_301	BoC07	35,675,639	AT1G53290		
BnC06	Ol12-E03B	74.9	BoC07_35576050_117	BoC07	35,576,050			

BnC06	bna4285	76.2	BoC07_35504618_268	BoC07	35,504,618	AT1G69370	AT1G69370	E
BnC06	bna1052	76.8	BoC07_34184488_301	BoC07	34,184,488	AT3G31720		
BnC06	bna0650	77	BoC07_33598518_273	BoC07	33,598,518	AT1G72960	AT1G72960	E
BnC06	bna2529	77.2	BoC07_32751398_304	BoC07	32,751,398	AT1G74490	AT1G74490	E
BnC06	FITO095	77.4						
BnC06	BEN185	77.4						
BnC06	BGO156A	77.4	BoC07_32557010_172	BoC07	32,557,010	AT1G55610		
BnC06	bna4278	77.4	BoScaffold000356_98841_301			AT5G61330		
BnC06	bna1006	77.9	BoC06_33759140_301			AT2G22930		
BnC06	bna4628	77.9	BoC04_10754934_301			AT3G61190		
BnC06	bna0656	79.5	BoC07_16839511_301			AT3G63080	AT3G63080	N
BnC06	BnGMS147B	79.5				AT3G63010	AT3G63010	N
BnC06	BGR99B	85.8	BoC07_31854860_133	BoC07	31,854,860	AT4G19239		
BnC06	bna0867	96.1	BoC07_29771104_301	BoC07	29,771,104	AT3G19790		
BnC06	Ol10-F09	97.5	BoC07_28931103_119	BoC07	28,931,103	AT3G57870	AT3G57870	N
BnC06	bna0892	98.6	BoC05_7077119_301			AT3G44350		
BnC06	bna4154	99.6	BoC07_27663436_303	BoC07	27,663,436	AT3G59830	AT3G59830	N
BnC06	bna4157	99.9	BoC07_27513541_301	BoC07	27,513,541	AT3G59510	AT3G59510	N
BnC06	bna4965	100.7	BoC07_16180589_301			AT3G61690	AT3G61690	N
BnC07	BoGMS1032	0						
BnC07	bna4251	3.7	BoC06_2074938_285			AT4G15590	AT4G15590	O/P
BnC07	bna4261	3.7	BoC06_2471432_305			AT4G07330	AT4G07330	O/P
BnC07	bna4236	3.7	BoC06_15105978_301	BoC06	15,105,978	AT4G06553	AT4G06553	O/P
BnC07	bna4162	3.7	BoC06_14770118_301	BoC06	14,770,118	AT5G34881		
BnC07	bna4234	3.7	BoC06_15054584_301	BoC06	15,054,584	AT3G19150		
BnC07	bna4726	3.7	BoC06_2100997_301			ATCG00350		

BnC07	bna4188	4.1	BoC06_13579405_301	BoC06	13,579,405	AT5G05180	AT5G05180	R
BnC07	bna4191	4.5	BoScaffold000960_1800_301			AT5G01830	AT5G01830	R
BnC07	bna4164	6.8	BoC06_14723271_301	BoC06	14,723,271	AT3G49810		
BnC07	bna4252	7.1	BoC06_2114909_301			AT1G52610		
BnC07	bna0721	7.4	BoC03_39349623_301			AT5G66630		
BnC07	bna4232	8.2	BoC06_15015127_301	BoC06	15,015,127	AT5G13225	AT5G13225	R
BnC07	bna4167	8.6	BoC06_14324578_301	BoC06	14,324,578	AT5G27590		
BnC07	bna4169	8.6	BoC06_14278577_301	BoC06	14,278,577			
BnC07	bna4177	8.6	BoC06_14027102_301	BoC06	14,027,102	AT3G46183		
BnC07	bna4186	8.6	BoC06_13624868_301	BoC06	13,624,868	AT1G45616		
BnC07	bna4187	8.7	BoC06_13586057_301	BoC06	13,586,057	AT2G31230		
BnC07	bna4903	8.8	BoC06_13687812_193	BoC06	13,687,812	AT3G58010		
BnC07	bna4179	8.9	BoC06_13994462_301	BoC06	13,994,462	AT3G16500		
BnC07	bna4172	9	BoC06_14197030_301	BoC06	14,197,030	AT4G08873		
BnC07	bna4180	10.2	BoC06_13886682_301	BoC06	13,886,682	AT2G16595	AT2G16595	H
BnC07	bna4176	10.2	BoC06_14046580_301	BoC06	14,046,580	AT2G16405	AT2G16405	H
BnC07	bna4165	10.2	BoC06_14627525_301	BoC06	14,627,525	AT2G15970	AT2G15970	H
BnC07	bna4724	11	BrA10_3001069_301			AT2G15860	AT2G15860	H
BnC07	bna4227	11	BoC06_14860771_301	BoC06	14,860,771	AT2G15780	AT2G15780	H
BnC07	bna4184	12	BoC06_13765187_302	BoC06	13,765,187	AT3G14800		
BnC07	bna4182	12	BoC02_25633759_301			AT3G28800		
BnC07	bna4183	12	BoC06_13809881_301	BoC06	13,809,881	AT2G13490		
BnC07	bna4902	12.1	BoC06_14314918_301	BoC06	14,314,918	AT2G11983		
BnC07	bna4181	12.2	BoC06_13872424_301	BoC06	13,872,424	AT2G15010	AT2G15010	H
BnC07	bna4189	12.2	BoC06_13553142_301	BoC06	13,553,142	AT2G15700	AT2G15700	H
BnC07	bna4174	12.2	BoC06_14122045_301	BoC06	14,122,045	AT1G36095		

BnC07	bna0459	12.5	BoC06_12726319_301	BoC06	12,726,319	AT5G28340		
BnC07	bna3768	12.5	BoC07_24416978_301			AT2G06840		
BnC07	bna4194	12.5	BoScaffold000410_19634_301			AT2G16940	AT2G16940	H
BnC07	bna4293	12.5	BoC06_12451594_301	BoC06	12,451,594			
BnC07	bna3967	12.5	BoC06_9908054_301			AT3G04600		
BnC07	bna4193	12.5	BoC06_13409803_301	BoC06	13,409,803	AT1G37110		
BnC07	bna4195	12.5	BoScaffold000410_40826_301			AT5G38285		
BnC07	bna4258	13.1	BoC06_2366458_283			AT5G60090		
BnC07	bna4808	13.1	BoC06_15175329_301	BoC06	15,175,329	AT2G15530	AT2G15530	H
BnC07	bna4255	13.1	BoC06_2247440_301			AT5G37500		
BnC07	bna4907	13.1	BrScaffold000239_42138_304			AT1G48260		
BnC07	bna4238	13.1	BoC06_15360330_301	BoC06	15,360,330	AT2G01220		
BnC07	bna4242	13.1	BoC06_15665403_301	BoC06	15,665,403	AT1G01650		
BnC07	bna4228	13.1	BoC06_14888151_301	BoC06	14,888,151	AT3G25815		
BnC07	bna4226	13.1	BoC06_14828402_301	BoC06	14,828,402	AT2G25970		
BnC07	bna4229	13.1	BoC06_14926384_301	BoC06	14,926,384	AT2G15730	AT2G15730	H
BnC07	bna4266	13.1	BoC06_2778576_301			AT2G07100		
BnC07	bna4240	13.1	BoC06_15620608_301	BoC06	15,620,608	AT4G04040		
BnC07	bna4245	13.1	BoC06_15739861_301	BoC06	15,739,861	AT5G54710		
BnC07	bna4235	13.1	BoC06_15072864_301	BoC06	15,072,864	AT1G22490		
BnC07	bna4906	13.2	BoC06_2275925_301			AT3G22970		
BnC07	bna4725	13.4	BoC06_15756688_271	BoC06	15,756,688	AT2G15220	AT2G15220	H
BnC07	bna4247	13.6	BoC06_15801680_301	BoC06	15,801,680	AT5G02910		
BnC07	bna4248	13.6	BoC06_15878853_301	BoC06	15,878,853	AT2G14692	AT2G14692	H
BnC07	bna4161	13.6	BoC06_14804908_300	BoC06	14,804,908	AT1G06460		
BnC07	bna4263	13.6	BoC06_2490783_301			AT2G13690	AT2G13690	H

BnC07	Na12-F03	14.1	BoC06_2511274_275			AT4G07730	AT4G07730	O
BnC07	sNRH63	14.9						
BnC07	BRAS019	14.9						
BnC07	CB10297	15.2	BoC06_2953737_265			AT4G04840	AT4G04840	O
BnC07	BN25C2	15.7	BoC06_4577757_142			AT1G02910		
BnC07	sN0706	15.7	BoC06_15240254_357	BoC06	15,240,254	AT3G31365	AT3G31365	L
BnC07	CB10217	15.7	BoC06_5914112_163			AT3G57150		
BnC07	Na10-C01B	15.7	BoC06_5650953_186			AT5G01180		
BnC07	bna0901	16	BoC06_4584719_301			AT5G42380		
BnC07	bna4246	16	BoC06_15748825_301	BoC06	15,748,825	AT5G32598		
BnC07	bna4262	16				AT5G04290		
BnC07	bna4254	16	BoC06_2200019_301			AT3G32230	AT3G32230	L
BnC07	bna1003	17.3	BoC08_23087748_239	BoC08	23,087,748			
BnC07	bna4196	17.3	BoC08_23737763_301	BoC08	23,737,763	AT1G71140		
BnC07	bna0935	17.3	BoC06_3403068_301			AT3G33070	AT3G33070	L
BnC07	bna4202	17.8	BoC08_26204631_301	BoC08	26,204,631	AT5G47870		
BnC07	bna4203	17.8	BoC08_26244524_301	BoC08	26,244,524	AT3G23280	AT3G23280	F
BnC07	bna4201	17.9	BoScaffold000719_1655_301			AT4G14342		
BnC07	bna4200	18	BoC08_26146753_247	BoC08	26,146,753	AT3G23370	AT3G23370	F
BnC07	bna4198	18.1	BoC08_25975058_301	BoC08	25,975,058	AT3G23480	AT3G23480	F
BnC07	bna4199	18.2	BoC08_26108471_301	BoC08	26,108,471	AT3G46130	AT3G46130	M
BnC07	bna0731	25.3	BoC06_24097329_299	BoC06	24,097,329	AT4G18950		
BnC07	bna1041	30	BoC06_28284306_301	BoC06	28,284,306	AT3G48770	AT3G48770	M
BnC07	bna0474	31.6	BoC09_25920840_231			AT3G61390		
BnC07	bna1055	34.5	BoC06_31734439_276	BoC06	31,734,439	AT4G05360		
BnC07	sR12156B	35.7	BoC06_31818111_169	BoC06	31,818,111	AT5G16580		

BnC07	Ol10-D03D	37.6						
BnC07	bna4304	39.4	BoC06_32533026_301	BoC06	32,533,026	AT3G26920	AT3G26920	L
BnC07	bna4908	39.4	BoC06_32566425_301	BoC06	32,566,425	AT3G27000	AT3G27000	L
BnC07	bna4313	40.8	BrA06_21862527_241			AT3G29185	AT3G29185	L
BnC07	bna4315	40.8	BoC06_34137121_183	BoC06	34,137,121	AT3G29575	AT3G29575	L
BnC07	bna4328	41.4	BoC06_34261501_301	BoC06	34,261,501	AT4G17610	AT4G17610	U
BnC07	BGO169	44	BoC06_34086182_244	BoC06	34,086,182	AT3G09920		
BnC07	BEN119	62.9				AT5G28910		
BnC07	BEN206	72.4				AT4G30680		
BnC07	bna4346	80.5	BoC04_31242317_293			AT2G24360		
BnC07	bna4344	80.5	BoC06_37800109_301	BoC06	37,800,109	AT4G03420		
BnC07	bna4376	84	BoC06_39464119_301	BoC06	39,464,119	AT4G35060		
BnC07	bna4377	84	BoC06_39441907_301	BoC06	39,441,907	AT1G30050		
BnC07	bna4393	87.4	BoC06_43445980_301	BoC06	43,445,980	AT5G01430		
BnC07	bna4400	87.4	BrA04_2943957_136			AT3G55140		
BnC07	bna4399	87.4	BoC06_43166079_281	BoC06	43,166,079	AT4G08460		
BnC07	bna4394	87.4	BoC06_43427426_301	BoC06	43,427,426	AT4G24220	AT4G24220	U
BnC07	bna4390	87.7	BoC06_43509340_301	BoC06	43,509,340	AT4G24540	AT4G24540	U
BnC07	bna4388	87.7	BoC06_43624619_301	BoC06	43,624,619	AT2G23660		
BnC07	bna4385	88.7	BoC06_43770397_301	BoC06	43,770,397	AT3G25495		
BnC07	bna4384	89.7	BoC06_43790514_301	BoC06	43,790,514	AT4G25340	AT4G25340	U
BnC07	BoGMS373	92.2						
BnC07	bna3404	92.8	BoC06_44356546_277	BoC06	44,356,546	AT4G27070	AT4G27070	U
BnC07	bna3405	92.8	BoC06_44323786_278	BoC06	44,323,786	AT4G26950		
BnC07	bna3403	92.8	BoC06_44410862_275	BoC06	44,410,862	AT4G27320	AT4G27320	U
BnC07	bna3402	93.3	BoC06_44427102_264	BoC06	44,427,102	AT4G27400	AT4G27400	U

BnC07	bna3395	93.7	BoC06_44570444_256	BoC06	44,570,444	AT4G27910	AT4G27910	U
BnC08	bna4401	0	BoC08_1857108_292			AT4G09090	AT4G09090	P
BnC08	bna0848	1.5	BoC04_1469322_301			AT5G51340		
BnC08	bna5477	1.5	BoC08_1501208_309	BoC08	1,501,208	AT5G19420		
BnC08	BEN69	5.7	BoC08_8369969_220	BoC08	8,369,969	AT1G48380		
BnC08	BoGMS38	8.8						
BnC08	BoGMS351	8.8						
BnC08	BGO178	8.8	BoC08_18707834_252			AT4G07350	AT4G07350	P
BnC08	sS2331BA	9.3	BoC08_10903912_116	BoC08	10,903,912	AT5G06810		
BnC08	bna3762	9.8	BoC08_10999144_301	BoC08	10,999,144	AT4G37705	AT4G37705	U
BnC08	bna3761	9.8	BoC08_11122588_301	BoC08	11,122,588	AT5G28076		
BnC08	BGR1B	11.4	BoC08_11779145_169	BoC08	11,779,145	AT1G12790		
BnC08	BEN189B	11.4	BoC08_14236962_229			AT4G34590	AT4G34590	U
BnC08	bna4711	11.9	BoC08_10837538_302	BoC08	10,837,538	AT1G72220		
BnC08	bna3765	12.2	BoC08_10784717_302	BoC08	10,784,717	AT2G22490		
BnC08	bna3767	12.2	BoC08_11203620_301	BoC08	11,203,620	AT2G20630		
BnC08	bna0144	12.2	BoC08_11335222_301	BoC08	11,335,222	AT5G58270		
BnC08	bna0836	12.5	BoC07_6327946_301			AT5G28643		
BnC08	bna3764	13.5	BoC08_10796441_269	BoC08	10,796,441	AT3G46430		
BnC08	bna0435	13.7	BoC08_14452136_301			AT4G16630	AT4G16630	U
BnC08	bna3766	13.9	BoC08_10732119_233	BoC08	10,732,119	AT5G49630		
BnC08	bna0071	22.4	BoScaffold000394_170509_304			AT1G11910	AT1G11910	A
BnC08	bna4403	22.4	BoC08_18489803_301	BoC08	18,489,803	AT3G12780		
BnC08	bna4404	22.4	BoC08_18448987_301	BoC08	18,448,987	AT1G12220	AT1G12220	A
BnC08	bna0808	23	BoC08_18653316_301	BoC08	18,653,316	AT4G34730		
BnC08	BnGMS161	37.4						

BnC08	BnEMS20	43	BoC08_27420490_342	BoC08	27,420,490	AT3G51880	AT3G51880	N
BnC08	BoGMS1200	45.5						
BnC08	BEN235	45.5	BoC08_27486008_243	BoC08	27,486,008	AT3G51940	AT3G51940	N
BnC08	bna2853	45.8	BoScaffold000220_330745_301			AT5G52480		
BnC08	bna5804	56.4	BoC08_33347565_301	BoC08	33,347,565			
BnC08	bna5803	56.6	BoC08_33288852_301	BoC08	33,288,852	AT3G62650	AT3G62650	N
BnC08	bna4525	56.8	BoC08_33349656_301	BoC08	33,349,656	AT5G37665		
BnC08	bna4522	57	BoC08_33288852_301	BoC08	33,288,852	AT3G62650	AT3G62650	N
BnC08	bna4524	57.2	BoC08_33323382_301	BoC08	33,323,382	AT3G19580		
BnC08	bna4523	57.2	BoC08_33309801_301	BoC08	33,309,801	AT3G61530	AT3G61530	N
BnC08	BGO185	58.8	BoC08_33413955_175	BoC08	33,413,955	AT5G67350		
BnC08	bna4466	59.3	BoC08_33414439_301	BoC08	33,414,439	AT3G25890		
BnC08	bna5150	59.6	BoC08_33606017_250	BoC08	33,606,017	AT4G29220		
BnC08	bna0149	60.1	BoC08_33685221_301	BoC08	33,685,221	AT2G26330		
BnC08	bna5790	60.1	BoC08_33626856_301	BoC08	33,626,856	AT3G63460	AT3G63460	N
BnC08	bna4470	60.1	BoC08_33627426_309	BoC08	33,627,426	AT3G63460	AT3G63460	N
BnC08	bna4479	62.3	BoC08_34299811_301	BoC08	34,299,811	AT4G16800		
BnC08	bna4478	62.3	BoC08_34223188_172	BoC08	34,223,188	AT5G15490		
BnC08	bna4480	62.3	BoC08_34330172_271	BoC08	34,330,172	AT3G28210		
BnC08	bna4737	62.3	BoC08_34090345_302	BoC08	34,090,345	AT5G37260	AT5G37260	S
BnC08	bna5319	62.3	BoC08_34149820_297	BoC08	34,149,820	AT5G28643		
BnC08	bna4484	63	BoC08_34490256_301	BoC08	34,490,256	AT5G39590	AT5G39590	S
BnC08	bna4483	63	BoC08_34447669_301	BoC08	34,447,669			
BnC08	bna4485	63.3	BoC08_34515885_301	BoC08	34,515,885	AT3G58930	AT3G58930	N
BnC08	bna4486	63.3	BoC08_34516979_301	BoC08	34,516,979	AT3G58975	AT3G58975	N
BnC08	bna4481	63.9	BoC08_34360358_301	BoC08	34,360,358	AT3G31395	AT3G31395	L

BnC08	bna4490	63.9	BoC08_34974685_301	BoC08	34,974,685	AT3G28810	AT3G28810	L
BnC08	bna5795	64.6	BoC08_34507781_302	BoC08	34,507,781	AT3G28150	AT3G28150	L
BnC08	bna4488	64.6	BoC08_34885790_300	BoC08	34,885,790	AT1G60310		
BnC08	bna4496	64.6	BoC08_35193805_301	BoC08	35,193,805	AT2G22610	AT2G22610	I
BnC08	bna4497	64.6	BoC08_35216428_299	BoC08	35,216,428	AT2G22540	AT2G22540	I
BnC08	bna4498	64.6	BoC08_35252325_276	BoC08	35,252,325	AT5G44680		
BnC08	bna4499	64.6	BoC08_35278607_303	BoC08	35,278,607	AT2G22490	AT2G22490	I
BnC08	BEN52	64.9	BoC08_35597022_161	BoC08	35,597,022	AT2G21870	AT2G21870	I
BnC08	BGO183	69.1	BoC08_34682365_236	BoC08	34,682,365	AT5G11050		
BnC08	BoGMS125	86	BoC08_40929085_506	BoC08	40,929,085	AT1G03000	AT1G03000	A
BnC08	BrGMS394	89.3				AT1G44000		
BnC08	BrGMS375C	90.7				AT1G05180	AT1G05180	A
BnC08	BEN316	93.1						
BnC08	CB10028	95.4	BoC08_39012255_177	BoC08	39,012,255	AT1G16700	AT1G16700	A
BnC08	BnEMS860	96.7	BoC08_40829449_328	BoC08	40,829,449	AT1G03470		
BnC08	BoGMS1558	98.7						
BnC08	bna4554	100.2	BoC08_38862591_301	BoC08	38,862,591	AT2G32560	AT2G32560	J
BnC08	bna0059	100.2	BoC08_38862591_301	BoC08	38,862,591	AT2G32560	AT2G32560	J
BnC08	BEN82	100.9	BoC08_37025192_150	BoC08	37,025,192	AT1G17080	AT1G17080	A
BnC08	bna0480	101.3	BoC08_36969549_301	BoC08	36,969,549	AT5G32345		
BnC08	bna5692	101.8	BrA09_21897067_311					
BnC08	bna5320	101.8	BoC08_38551264_301	BoC08	38,551,264	AT1G22560		
BnC08	bna4551	102.1	BoC08_38792031_300	BoC08	38,792,031	AT3G31300		
BnC08	bna5810	102.4	BoC08_38521325_301	BoC08	38,521,325	AT1G12930	AT1G12930	A
BnC08	bna2602	102.4	BrA09_21897067_311					
BnC08	bna4543	102.4	BoC08_38528116_301	BoC08	38,528,116	AT1G12890	AT1G12890	A

BnC08	bna4544	102.4	BoC08_38555469_301	BoC08	38,555,469	AT1G12740	AT1G12740	A
BnC08	bna4547	102.4	BoC08_38663751_301	BoC08	38,663,751	AT1G12360	AT1G12360	A
BnC08	bna5179	102.9	BoC08_38982814_301	BoC08	38,982,814	AT1G11650	AT1G11650	A
BnC08	bna5813	103.4	BoC08_39218901_301	BoC08	39,218,901	AT1G10750	AT1G10750	A
BnC08	bna4558	103.4	BoC08_39223830_309	BoC08	39,223,830	AT1G10740	AT1G10740	A
BnC08	bna2785	104.6	BrA09_35647627_301			AT1G07910	AT1G07910	A
BnC08	bna5480	104.6	BoScaffold000330_47398_301			AT1G07520	AT1G07520	A
BnC08	bna5808	104.6	BoScaffold000330_198207_301			AT5G63195		
BnC08	bna4540	105.1	BoScaffold000330_49205_301			AT1G07520	AT1G07520	A
BnC08	bna4534	105.5	BoC08_40629883_301	BoC08	40,629,883	AT3G61172		
BnC08	bna4539	106	BoScaffold000330_354434_231			AT5G40150		
BnC08	bna4538	107.3	BoC08_40291476_301	BoC08	40,291,476	AT5G36690		
BnC08	bna2783	107.3	BrA09_35751967_301			AT1G07470	AT1G07470	A
BnC08	bna4537	107.4	BoC08_40327538_301	BoC08	40,327,538	AT5G03870		
BnC08	bna2782	107.5	BrA09_36016899_303			AT1G06440	AT1G06440	A
BnC08	bna5720	107.6	BrA09_36111852_301			AT1G05950	AT1G05950	A
BnC08	bna5807	107.7	BoC08_40306294_301	BoC08	40,306,294	AT1G05360	AT1G05360	A
BnC08	bna5806	107.7	BoC08_40937947_301	BoC08	40,937,947	AT1G02990	AT1G02990	A
BnC08	bna4531	107.7	BoC08_40937947_301	BoC08	40,937,947	AT1G02990	AT1G02990	A
BnC08	bna4527	109.6	BoC08_41383351_301	BoC08	41,383,351	AT1G01950	AT1G01950	A
BnC08	bna5428	109.6	BoC08_41331400_301	BoC08	41,331,400	AT1G01770	AT1G01770	A
BnC08	BoGMS1166	110.7						
BnC08	bna4795	115.6	BoScaffold000330_36569_301			AT3G44091		
BnC08	bna5719	115.6	BrA09_36304337_301			AT1G04860	AT1G04860	A
BnC08	BEN136B	119	BoC08_41173283_131	BoC08	41,173,283	AT1G01090		
BnC08	bna2773	132.4	BrA09_36753704_301			AT1G68080		

BnC08	bna5805	137.6	BoC08_41421132_301	BoC08	41,421,132	AT3G42410		
BnC08	bna4526	140.1	BoC08_41433239_301	BoC08	41,433,239	AT1G02080		
BnC08	BEN136A	141.3	BoC08_41173283_131	BoC08	41,173,283	AT1G01090		
BnC08	BrGMS375A	143.7				AT1G05180	AT1G05180	A
BnC08	BrGMS375B	147.1				AT1G05180	AT1G05180	A
BnC08	bna2772	151.7	BrA09_36860095_301	BrA09	36,860,095	AT1G01070		
BnC08	bna2776	152.7	BrA09_36402548_279	BrA09	36,402,548	AT5G16410		
BnC08	bna4668	152.7	BrA09_36327642_302	BrA09	36,327,642	AT1G04630		
BnC08	bna2784	152.7	BrA09_35707655_301	BrA09	35,707,655	AT1G07560	AT1G07560	A
BnC08	bna2791	154.8	BrA09_35278168_284	BrA09	35,278,168	AT1G51200		
BnC08	bna5099	154.8	BrA09_35278168_284	BrA09	35,278,168	AT1G51200		
BnC08	bna2790	155.1	BrA09_35321451_301	BrA09	35,321,451	AT1G09530	AT1G09530	A
BnC08	bna2789	155.1	BrA09_35389195_301	BrA09	35,389,195	AT3G14025		
BnC08	bna2796	158.2	BrA09_33398631_301	BrA09	33,398,631	AT1G16445	AT1G16445	A
BnC08	bna5427	160.7	BoC08_35949347_304			AT2G20830	AT2G20830	H
BnC08	bna2799	160.7	BrA09_32229790_301	BrA09	32,229,790	AT2G21120	AT2G21120	H
BnC08	bna5723	161.1	BrA09_32177679_301	BrA09	32,177,679	AT2G21230	AT2G21230	I
BnC08	bna4491	162.5	BoC08_34986249_301			AT3G19770		
BnC08	bna2947	162.7	BrA09_5056173_301			AT2G22480	AT2G22480	I
BnC08	bna4493	162.9	BoC08_35073478_301			AT2G22770	AT2G22770	I
BnC08	bna2939	164	BrA09_31788228_301	BrA09	31,788,228	AT1G78500		
BnC08	BoGMS1382	165.4						
BnC08	BnEMS14	165.7	BoC08_36424147_266					
BnC08	BoGMS1308	166.8						
BnC08	BoGMS586	167.9	BoC08_34092882_250			AT2G25052	AT2G25052	I
BnC08	BrGMS5	171.5				AT4G05490		

BnC08	bna5724	172.3	BrA09_30571654_300	BrA09	30,571,654	AT3G63030	AT3G63030	N
BnC08	bna4521	172.3	BoC08_33259513_301			AT3G62620	AT3G62620	N
BnC09	bna1478	0	BrA02_23454785_301			AT3G30775		
BnC09	bna5851	4.4	BoC09_171077_274	BoC09	171,077	AT4G14580		
BnC09	BEN332C	6	BoC09_186601_237	BoC09	186,601	AT4G00660	AT4G00660	O
BnC09	BGO199B	10	BoC09_193617_190	BoC09	193,617	AT4G00710	AT4G00710	O
BnC09	BoGMS1072	16.9						
BnC09	BGO199A	20.3	BoC09_193617_190	BoC09	193,617	AT4G00710	AT4G00710	O
BnC09	BEN343	21.9	BoC09_30393262_235			AT3G23690		
BnC09	bna5746	23.7	BrA09_696711_301			AT4G00900	AT4G00900	O
BnC09	bna4581	24.9	BoC09_2014248_302	BoC09	2,014,248	AT4G07810	AT4G07810	O
BnC09	bna4580	25.7	BoC09_2017095_301	BoC09	2,017,095	AT3G51360		
BnC09	bna4579	26.3	BoC09_2055747_260	BoC09	2,055,747	AT3G27860	AT3G27860	L
BnC09	bna4577	26.4	BoC09_2061471_219	BoC09	2,061,471	AT2G44790		
BnC09	bna5960	26.5	BoC09_2063866_301	BoC09	2,063,866	AT2G35280		
BnC09	bna3638	27.5	BoC09_2186157_301	BoC09	2,186,157	AT3G28580	AT3G28580	L
BnC09	bna4708	28	BoC09_2165074_301	BoC09	2,165,074	AT5G01820		
BnC09	BoGMS1484	30.3						
BnC09	BrGMS725B	31.4				AT5G48230		
BnC09	BEN55A	31.9	BoC09_2708553_177	BoC09	2,708,553	AT4G27500		
BnC09	FITO135B	32.2						
BnC09	FITO136	35.2						
BnC09	bna4585	45.1	BoC09_1990112_301			AT3G27400	AT3G27400	L
BnC09	BnEMS820B	76.9						
BnC09	bna4594	78.5	BoC09_23062819_301			AT3G18165		
BnC09	bna4592	79.1	BoC09_5057885_301	BoC09	5,057,885	AT5G67050	AT5G67050	X

BnC09	bna4591	79.1	BoC09_5058499_301	BoC09	5,058,499	AT5G67050	AT5G67050	X
BnC09	bna4761	85.6	BoC09_6686378_301	BoC09	6,686,378	AT1G48490	AT1G48490	C
BnC09	BnGMS213A	85.6				AT1G67330		
BnC09	BGO196	87.2	BoC09_6732672_119	BoC09	6,732,672	AT2G37110		
BnC09	bna0971	89.1	BoC09_7208308_301	BoC09	7,208,308	AT5G45360		
BnC09	bna4601	89.1	BoScaffold000035_P2_283375_259			AT5G16190		
BnC09	bna4602	89.4	BoScaffold000035_P2_669076_301			AT1G54560	AT1G54560	C
BnC09	bna3857	89.7	BoScaffold000035_P2_1472078_301			AT4G16870		
BnC09	bna1011	89.7	BoC07_13141450_301			AT1G61760	AT1G61760	D
BnC09	Na10-C01C	90	BoC09_8400120_250	BoC09	8,400,120	AT5G25615		
BnC09	BEN203	91.9	BoC09_18992253_333	BoC09	18,992,253	AT5G38600		
BnC09	bna0875	93	BoC09_34857697_301	BoC09	34,857,697	AT1G61215	AT1G61215	D
BnC09	BrGMS726A	102.5						
**Total**	**2115**	**2477.4**	**1923**	**46**	**1309**	**1930**	**1033**	**198**

[a] The left, middle, and right refers the chromosome or scaffold of the *B. rapa* or *B. oleracea*, the physical position of the homoeologous locus in *B. rapa* or *B. oleracea* chromosomes, and the matching base pairs of the homoeologous locus in *B. rapa* or *B. oleracea*, respectively.

[b] The colors of the homoeologous collinear fragments are same as to the colors of the *B. rapa* and *B. oleracea* chromosomes in Figure 2.

[c] The capital letters represent the conserved blocks defined by Schranz et al. (2006). The colors of the conserved blocks are same as to the colors of the conserved blocks in Figure 3.
