# Supplementary material for: A Complex Recombination Pattern in the Genome of Allotetraploid Brassica napus as Revealed by a High-Density Genetic Map
Source: PLoS One. 2014 Oct 30;9(10):e109910. doi: 10.1371/journal.pone.0109910 (PMC4214627; doi:10.1371/journal.pone.0109910)
Supplement: Table S2 — The primer sequences of the SSR markers used in the HJ DH population genetic linkage map construction. (PDF) [file pone.0109910.s002.pdf]

Table S2. The primer sequences of the SSR markers used in the HJ DH population genetic linkage map construction

Marker	F_primer (5'--3')	R_primer (5'--3')
**BEN2**	GGGCAGGGAGCTAAGTAACC	AACAGCTCCACCGTTACTGG
**BEN6**	GTGGAAGATCGAGTGCGAAT	CCTCTGACTCCATCTCAGCC
**BEN9**	TGGAGCAACAACCTGTGAAG	GTGGTGAGGGAAGGAGTTGA
**BEN10**	AACAGCAGAGAAGCGAGGAG	GAAGAGGGATCGGAAGAAGG
**BEN14**	CAAGAGATCGTAGCTTCCGC	CAGTTGGCCAGAAGTGCATA
**BEN16**	CTGGTCCAATGAGCAAACCT	GGAGGTGGTCCTTTCTTTCC
**BEN25**	CGTCTACTGCCCTCAATGGT	CTAGCGAGGATTCCGATGAG
**BEN28**	TCGCAAACGTCATCACTCTC	GCCGTCGCGTCATATATTCT
**BEN30**	GGAGAATGGTAAGCGAGAAGC	TCCACACCTTGAAAGGGTTC
**BEN32**	GAGATGCTTCTATCGCCTGG	CTCGTCGAGGACTCTCATCC
**BEN43**	GTGGTGATGGTACATCTGCG	CCTTTGGTGGTAGTGGTGGT
**BEN45**	TGTTCGTATTCTCGCGACTG	CGATTTCCTTCAAGCTCTGC
**BEN48**	TGGGCTCCTACAACCGTAAC	TCTGATCCATCTGATGCTGC
**BEN50**	TCAATCCTCGAATCTCGCTT	CGATCCCTGGAGGATACCTT
**BEN52**	TTCAAGGATGAGGAAATGCC	ACAGCGAAAGGGAACAAAGA
**BEN55**	ACGCCATTTGCATTCTTCTC	TCTGTGCTTCCACAATCAGC
**BEN59**	GATTCGCTTCCCTTCCTTCT	TTGAGCTTCTCTAGCTCCGC
**BEN68**	CGTCACACGCCACATTATTC	AGTAATCAATCACCTGCGGC
**BEN69**	AACCCTGAAGAATCCCGTCT	GGTTGGCTACCAGTTTCAGG
**BEN78**	CTGTGGTTCCTGCTCTCTCC	AGTGAAATTGGGTTTCGCAC
**BEN81**	GAAAGCGGCAAAGTCATTCT	GAATCCGAAACTCCGAGACA
**BEN82**	AAGGATTTGCTGAGGAAGCA	CAGCATGATAACCATGGACG
**BEN83**	TGATCGGTCAAAGTCAACCA	GATCCACCGTGCTTCTCTGT
**BEN84**	TTGATCCAATAGCCATGACG	TCGAGGACGAGATGAGACCT
**BEN95**	GAAGACGAAGATGCCGTAGC	TGGAATTTGCTGGAGGTTTC
**BEN98**	ATCACTTCCGGTAATGCGAC	CTGATTTGAAGGCTCTTCCG
**BEN116**	ACGCTCAATCTCACAGCCTT	ATGAAACTTTCTCGGAGGCA
**BEN119**	ATGGACAGGAGATGGTGGAG	GGTATCCACGGCTTGTGACT

**BEN123**	AAAGACAAAGACTTGCGCGT	CTCCCACTCAGACTTCTCGG
**BEN133**	TTGGGTGGTAAGAAAGATGGA	ACTTGCTCGTGTGGAAGACC
**BEN134**	AGCTAAGCTAAAGGCGGAGG	AAGCCCAAATCAAGCATCTG
**BEN136**	TGCGAGGATCCCAAGTTTAC	CAAACTGTTTCCTTGTGGCA
**BEN140**	ACAGGACACGATACGGGAGA	GGCTTGCTTCAGTCCGTAAG
**BEN178**	CTCGCTAGGAAACGGCATAG	AGAGATCCAGCCTTTGTTGC
**BEN185**	GGAGGAAGGGAACAGTGTGA	TCCGGAAGTGAAGTAATCGG
**BEN186**	TCTTCTTCCTCCTCAGCTCG	TCTTCCTCTTCTCGCTCTCG
**BEN189**	TTAAACCCGGTCCTGTTCTG	CATGAGCTCAGGAGGAGGAG
**BEN190**	CCGGAAAGATCAGGTAACGA	AGGAGCGCTTGTGTCAATCT
**BEN200**	AAGCAAACAAACACATGCCA	TGCGGAAGAAGAAGAAAGGA
**BEN203**	TGACTCAAATCAACCTCTCACG	TTTGTCACTCGCCATCTCTG
**BEN206**	CGCACGCTTTATGTTCTGTC	CGTCTCCTGGTGGTACCTGT
**BEN210**	GCAAGGCTTTCTCACCAAGT	TGAAACAATCACGCAAAGGA
**BEN211**	ACACGCTCACTGGAGAATCC	TTTCGTCGGTGTAGTTGTCG
**BEN216**	ATTGTCTTTGCTCCGACACC	TTGAGAAGCGGCGTAGAAAT
**BEN228**	GACCAATCCTCCTCCTCCTC	TTGAAGAGCTGAGTCGGGAT
**BEN229**	TTCAAACACGGTCTCTGCAC	GTCCAGCGATTGAAGATGGT
**BEN235**	TCTCGATGGGAAAGTGGAAC	CATGGCACTGATCCAATCAC
**BEN236**	TCGGTGGAAGCTAATCTTGG	GGGATTGATCGGAACTCGTA
**BEN239**	GAAGGACTCGTCAAGATGGC	CAGCTGGAGTACAGATCGCA
**BEN251**	AAATCTGGGATCTGCACGAC	CGTATCCCTGCTGCTTCTTC
**BEN253**	ATCTTCGTGGCTGCGTGT	GCCAACATTCGGAAAGAGAG
**BEN254**	TTCAATGGAAAGATCCCGAG	TTGCAGAAGCGTAAAGCAGA
**BEN260**	AGTCGGCTAAGGAGAAAGCC	AACATCCACATCTCCAAGCC
**BEN266**	AGGAACGGTTGCTTACGAGA	ATCCCTCAGCTCACTCCTCA
**BEN269**	GAATGGGTACACGCCTGAGT	CCGATGTTGATGTTCCTGTG
**BEN271**	AGGTTCGTTGGTCGGTAGTG	TTGTGGAACGAGAACTGCTG
**BEN278**	AGCTGTGAGGGAGTCAAGGA	TTTCGCGGTATCTACGGAAC
**BEN281**	GTGGATTTCTGCTCACCCAT	ATGTGAGGAGGGTGAAGGTG

**BEN284**	CAAGCGGAGAGGATGAAGAG	GGAGCTTGACCTTGAGCAAC
**BEN285**	ATCCGGGTTTGCACATAGAG	CCAGAGGATGTTCAGTGGGT
**BEN288**	CAAACCGGTCACTAAACCGT	GGAAGCTGAAGAAGCACGTC
**BEN296**	ACGCCGTGTCAGAAGAAGAT	AACAGGCTTTAACGAGGTGG
**BEN312**	TAAACCTCCGCTTAAAGCCC	ATCCGACTTCGAATTCCTCC
**BEN313**	CGAGAGTTTGAGTGGCTGTG	TAATTCGCCGAGAGGAAGAA
**BEN316**	GCTTGCTTCTCTTGGCAATC	CCCACGGAGATAGATGGAGA
**BEN320**	CGTTGGTTTTTGCATGTTGA	GCTGCTCTCATTTTGGAAGC
**BEN327**	GCAAGATTCTTCCCGTTCAA	GCGATCTGATTAGCCCTTTG
**BEN330**	CCTTTCTGGGGATTTGATTG	ATCCGCAGATTGGATCTTTG
**BEN332**	CTCCGCAAGTTTCCTTTTTG	CCGACCGAGACTGGAAATTA
**BEN334**	GCGATAGGTGTTCAGGGAGA	TTTCGCAGTCATACTCGTCG
**BEN335**	ATGACTCGTGATCTCCTCGG	AAATCGAGACGTTTCTCCCA
**BEN337**	TTACTGAACCAACCTCGTCG	GGAGAGAGCGTCTTTGGTTG
**BEN341**	TGGAAACATTAGCTTGGAACC	GCAACTCGTCTTGTTTCATT
**BEN343**	TCTTTGGAAGATGACCCCAG	TCCAGCTTCATCATGTCTTACA
**BEN348**	GATTAGCTCTGGTTTCCCCC	AGAAAATCATCACCCCCTCC
**BEN349**	GTGTGGTCGTCAATTTGTGC	AGGAGGAGGAAGAGCAGAGG
**BEN363**	CCTTGGATTCAAAGAGCAGG	TCAGGGCGGAGTTTACAAAG
**BEN374**	CTGACGAGGCGATTAAGAGG	TCACCATCTCAGCAGTAGCG
**BEN376**	ATTAGGGCTGAGTCTGGGCT	GGAGCTTAACGATGTGGAACA
**BEN387**	TGAAAGGGTCGAATTCGTTT	ATGAGTTTCGCCTAACGACG
**BEN391**	ACCACCATGCCTAAGTCGTC	AACTAAGGTGGGGGATTTGG
**BEN396**	GTAACAGCGTCTTGCGACAG	TCCTCTGATCCACTCCATCC
**BEN406**	CGGGGAGGTTTTAAAGCAG	CACGGATTTGACACCCTCTT
**BEN410**	TGTTCCTCCACCACAACAAA	GTGCTTCTGCTTCTTTTGGC
**BEN420**	GCAGCAAAGGCAGGTCTACT	AGGATTGAAGGCGACAGAGA
**BEN425**	CCGGGAGTAACTGACCTCAA	TAGTCTCCTTACAACGCCCC
**BGO001**	TTCGCTATCTCTCGACCGTT	ATCCTCGCCGTGATATTCCT
**BGO006**	AACGGCTATGGCTTGTATGG	GACTCTTCGGTCTCGCTCAG

**BGO029**	GCAACTGCACAGAGTTTGGA	ATCATCGTGATTCCTCTCGC
**BGO041**	GAAGGACTCGTCAAGATGGC	CTGGAGTACAGATCGCAGCA
**BGO044**	TCGCACTTGAATCTCCCTCT	CGAGTCAACTCGGTGAGACA
**BGO054**	TAAGTTCTTCGCCGAGATCC	CAAGCGCAAACTCAAGCATA
**BGO055**	CGATCACTGCTTCACTGCAT	TAGTTAGGTGGTCGGCCAAG
**BGO056**	AGGTTCGTTGGTCGGTAGTG	TTGTGGAACGAGAACTGCTG
**BGO078**	ACTTACACCTCTTGGTGGCG	CAGCTTCTCCAAGCAGATCC
**BGO090**	GAGGTGTGGCTAGTGGTGGT	CGGTGAATGAAGTCCATCCT
**BGO108**	TCGCGTGCCAAACAATAGTA	ATTCACTTCTTCCATTGCGG
**BGO114**	CCTTTATTCCATTTCCGGGT	TGGACCAGCCAAGGTTAGTT
**BGO120**	TGGCATTTGAGTAGTGGCCT	AGTGGCCCACACTTCCATAG
**BGO125**	GAACCACCAAGGGAAAGTCA	CGATTCAGCTAATCCAAGCC
**BGO140**	GGACGTGCGGATCAAGATA	TGACAACGGGATATTCAAAGG
**BGO141**	TTTCCAGGGATGGTTATGGA	CCGGCTCTGATACGCATACT
**BGO146**	CCTAGTCCATGCTGTTTCGG	CCCAAGGAAGATGGTGAAGA
**BGO151**	GAGATCGTCGGTGAGAGAGC	TACAGCGGACTCCAAACCTC
**BGO153**	CGACGAAAGGAAGAAACCCT	AGAAGGAAAGTAGCCGCCTC
**BGO156**	TGTGTCAGCCACAAGAGAGG	TCGCCATGATGTTATCTGGA
**BGO160**	CCAGACACAACAAACATGGC	TTCGAGGAGAGTTTGGGAGA
**BGO161**	TTTGGTTACTAACACGCCCG	AGGACTCGAAATGGATGTGC
**BGO169**	CTAATCCACCACCGTCACCT	TTGTGTTTAGAGGCGCAATG
**BGO178**	ACTAACAAGCACCGGAGACG	AGCACCTGTAGCGGGTTCTA
**BGO179**	TTCTTCGCCTTACCTCCTGA	AACACAAGTGAGACATTGGCA
**BGO183**	AAGAAAGGTCCATGTGTCCG	CGAAATGCCAGATTCCAAAT
**BGO185**	GATAACCGATCGGACATTCG	AAGGCCAGTCCAACCAGAG
**BGO196**	GTGAAGGGAAGTCCCAGACA	TCGACCACAAGACCTGTTCA
**BGO199**	CAATCGAATCGACATGAACG	TGCGTTGTATTAGATGCACGA
**BGR1**	TGACTTTCTTGAGGGTATGC	AGCATTGGTATCCAAGACAA
**BGR2**	CGTACGCTTGTGTGAGTAGA	CAGCAGGTAATAACAGAGGG
**BGR6**	GAAGAAGCTTTACTCGGTGA	TTATGTTAGGGCTTGTCCTG

**BGR8**	ATAGTCATCGGAGGTCGATT	AAATCCACTTTCTCTCTCCC
**BGR16**	TCCGTCTCTTCTTTTCTTGA	CGCTGTAGTGTTCTTCTGGT
**BGR20**	CGTAACTTGTTGTTGTCACG	CCTACCATTGAAGATGGTCT
**BGR23**	GTCGGATCAGCTCTGTGTAT	CTCTTCTTCTCTCTCATCGC
**BGR34**	GGACCTTGGTTCAGTGTGTA	GAGTAGGATGGGTGCATCT
**BGR35**	CCGCCTAAGCTCATTAACTA	CACCTAGTCACCGGAATAAA
**BGR38**	TGCTCTTACTCCTCTGCTCT	CCAACTCTTCTAGGATCGC
**BGR42**	AGTCGCTAGAGGTTTTTCCT	CAGCACTAAGCATCTCATCA
**BGR43**	GTGCTCCTTTCAAATCTACG	GACAGGATGATGTGGTATCC
**BGR45**	ATCGTTTCTCTCTCCCTCTC	ATATCCGTTGGAGAAGACG
**BGR49**	GATATTGGTCGTTGGAGTTC	CCTCTCTCGCCATTACTCTA
**BGR50**	GATGGCCATGGAGTAGATAA	GACCCATTCGTACTTGAGTT
**BGR51**	GAAGGGATAGAGAGAGAGGC	GGAAGAGAAGAACAGAAGCA
**BGR52**	CAGAATCGGAGTAACCAACT	AAAATCACCGATCCTCTTCT
**BGR54**	CTTCCCTTCTTCAGTTGCT	GGTTTGAGAAATGATTCACG
**BGR55**	AGACGAAAAGGTTGCTTCTT	CTTTTAAGCTTCCTTCCTCC
**BGR56**	GGGAGGAAGATGGAGATTTA	GAACACTTATCAGCCGTTTC
**BGR58**	AGCAGTGTATCCGCACTATC	TAATATTGTTTGGAGCAGGC
**BGR60**	GAAACTGGAGAAAGGAAAGG	CCATCGTCACGAACTACATA
**BGR61**	GCCACAAAACTACTAGATGGA	CAAACTATCCATGTCTTCCG
**BGR64**	CTCCTGTAAACCACCATCAT	CTCCGTTAACAACCATCATT
**BGR65**	GTTGAAAGCTTACGCAGTCT	ACCTGAAGCTCTGAAACTCA
**BGR66**	GCTCTTCATGATGTTCCATT	CCTTAGATGAACCTACATTCG
**BGR69**	AGACGAGTCATCTCCATCG	GCAACATGAAGAAGAAGAGG
**BGR70**	AGAGAGTTTCAGGCTTTGGT	GGGAAAAGAGTGGAGAGAGT
**BGR71**	TCTACTTCTCTCCCTCCTCC	AGAGAGAAATGGGAGAAACC
**BGR74**	ACAAACACCTCGATCTCATC	GGGGGAGAGATAGATACCTG
**BGR75**	ACACGTCTCCATGAAAAAGA	GTTCCATGGACAGAAGAAGA
**BGR80**	TCGATATGTATTCAGGGGAG	GAGTCATCAGCATCTTCCTC
**BGR83**	TCTTTCTCAGTCCCTCTTGA	GTTCCATGCAAATCCATAGT

**BGR84**	CCTCCTTACCCTAACCCTAA	CCATAACCAGGTGAGGAAG
**BGR87**	AGGATGGTGAAGTGAAACAG	TAATCTCCGTTGATCTCCAG
**BGR88**	GTTCCAACCATAAGCCCT	GATAAAACCTGCGGTCAC
**BGR89**	ATAGTAAACCACCACAACCG	ACTATAGGCCCTCTCTGTCC
**BGR93**	CAAGTCAAGTCCCAATCAAC	TTGCTGAGAGAGTGATGGA
**BGR98**	GGTTATACCTTTTGGGAAGG	CAGTGTCTCCCTTTGTTCTC
**BGR99**	GGATAAGCGATACACACCTT	TATGAGATACTGGTGCGTGT
**BGR100**	CTTAAAATCCTCCAGGAACC	CTCTTAGCATTGGGATTAGC
**BnEMS14**	TTTGTGAGTGGTCTTGATTT	AATGGATAATACTGATGCTCTT
**BnEMS20**	CTCTATCCGAAGTGAATGATG	GTCGTCAACCCACACAAA
**BnEMS57**	ATGTTGGGTAAAGGCTACTG	CTGCTCTCTGTTCTGCTTATC
**BnEMS59**	ACACCACCGTCCAGAAGA	TATCACTCTCAAACCTCCCA
**BnEMS60**	TATCACTCTCAAACCTCCCA	ACACCACCGTCCAGAAGA
**BnEMS82**	AGAAGGAAGGTTTGCTCACT	CAACACAGAGATTGGTTCATC
**BnEMS146**	AGGTGATTTCTCTTCTCGGT	TACTTTGTCTGAGTGTGGTGG
**BnEMS439**	TCTATAGCCAGCGCTCTTAC	TAGTCATTGCACATTGCTTC
**BnEMS634**	GCACACACCTTTCTTTCTTC	GTCCTCGGTGCTTATGTTAG
**BnEMS695**	GCTGGTAAATTGTTCCATTA	CGTAAGGAAGACCAAGAACA
**BnEMS812**	ATTCTCTGCCTCTAGGCTTT	AAGACATGTTTGATTCCGAC
**BnEMS820**	ATACTGTCAAGAAGCCCAAA	AAGAATCCAACCATGGATAA
**BnEMS824**	CACAATCCCACTCTCAAGTT	TCCATTTCAAGAAGTAGGGA
**BnEMS843**	CGGAGTCCAATACGAATAAG	GGATGTTGCTAGGTAAGCAC
**BnEMS860**	TGGTGACTACTACTAAATCGAGA	GAGAGTAGAATGAAGCCACG
**BnEMS973**	GTCTTCGTTGTGTCCTCAGT	TAATGATGCTTCTCGGTCTT
**BnEMS993**	CCTATTCGACCACAATGAAT	GAGAGTTGGCTCGAGTGTAG
**BnEMS994**	TGATTCCTCTCCTCCTCATA	TGCTAGCTTCCTTCATAAGC
**BnEMS1012**	ACCTAACTCGCACTTCTTCA	TTGGTAGAGAAACCAGAGGA
**BnEMS1036**	CGTACCTGAGGCAGTTACTC	GGAATCCGTTATTTGATTGA
**BnEMS1072**	GATATCTTGATTAGGGTTCTTAT	GTTCTCCTGCATTTAGATCG
**BnEMS1084**	AAGGTGATGATGGTAAATGC	TCTCTCTGTTCGATGTCCTT

**BnEMS1116**	GAAATTGGCCACATGTATTT	CCAAGACCTCCCTATCTTCT
**BnEMS1119**	ACTGGAGTTTCAATTGGATG	CATCATCTTCAGCACTAGCA
**BnEMS1124**	ATGTTTCCTTTGCTCGTTAG	GAAGCCCAACATTATTCAAA
**BnEMS1144**	ATATCTCTCTTTGCGAACCC	TGGATCTATTAGCTGCCATT
**BnEMS1156**	ATCTGAACAATCAATCTCCG	TTACGATACGCTCTCCTCAT
**BnEMS1171**	CTGTGAGGAGAGAAAGGATG	AGCTTACAGCTTTCAACAGG
**BnEMS1173**	CGAAATCCTCACAATCTCAG	CGGAGATTGATTGTTCAGAT
**BnEMS1184**	AACGTCAGAGAAATGTGGAC	GGCATTACTCAACGGTGTAT
**BnEMS1185**	CTTCTTCAACCTCTCCCTTT	GTGTTGAGCTTCTTGAGGAC
**BnGMS9**	GGAGGAGGAAGAGGAAGATA	GGAGCTAGTGAAGCCAATAA
**BnGMS87**	TTCTTCACTGATCCCACTTT	TGTCTGTAGCATTGATTGGA
**BnGMS131**	GAAGAAGAACATGGCTTGAG	GCTTCAATTCAATCTTCACC
**BnGMS133**	CTTAAATTGTCCCTCCTCCT	AAGAAATGAGCTTGAAGCAG
**BnGMS147**	AAGCAGTGAGCACTACACAA	CTTCTTGCCTAACAATGACC
**BnGMS153**	CTCAATGCTAAGGATATCGG	TCTGATCTTTCAGACTCCGT
**BnGMS161**	GAGTCTTGCTTTATCATGCC	GATCTTTCACTTCAGCCTTG
**BnGMS175**	TGATCTTCCTGAGAATCAGC	GAGGAAGAAGGAGAGAGGAA
**BnGMS198**	AGTTTCGGATACTTTCGGAT	CATGTCGACAAGAGAAACAA
**BnGMS205**	AAGAGAGACAGCGTGTTGTT	GACTCTAGGAGAGTAGACGGC
**BnGMS208**	GGGTATTTGAGGGTATTGGT	CATGTCGACAAGAGAAACAA
**BnGMS213**	GTAGTACGGAGATGCGTGAT	AAAGAACGAGTTGACTTTCG
**BnGMS249**	AATAATAAGAAGGGATGGAA	CACCTTAAGTGGTTTCTTGC
**BnGMS265**	ATAATCGTCGGTTCGATGTA	GCTTTGTGTGAAGGAATTTG
**BnGMS584**	GTCCGTGTCGATCTACATTT	CAAGACCATTGAGCTTTCTT
**BoGMS38**	CCTTTGTTCAGCACCATC	CGTGGATTGAGAAGTAGCAGT
**BoGMS125**	GTTGGCTTCACTTCTCCTACT	GCATCTCATACATCAAAGGG
**BoGMS154**	CTACGGCTTCGTCTCGTC	GCTTTGCGTTACATCTTTCT
**BoGMS197**	CCCTCTTGATTACAACGAAA	GTATGTTTACCTGGACCTGACT
**BoGMS204**	CCGATAGTAGTCAAAGCCTG	GAAACCTAAACGGCACAA
**BoGMS314**	AGAAAGATGGAGAAGACGAAG	CGAGAAGAAGAGAGGAAAGAA

**BoGMS319**	GTCTTCCTTCATCCTTCTCC	GGGCAACTTCGTCATTATC
**BoGMS328**	TTTATTCGGTTTATTCGGTTT	GAAGGTGAGTCTTGATGGTG
**BoGMS351**	AGGTGGTGTAGTTGAGGAGAC	TGACAGATGGGATAAGACAAA
**BoGMS373**	CTCTGACGAAGATGGCTG	AAGTCTATGTTTGGATAAGGGA
**BoGMS490**	CTTGAGTCGTTTGTTTGTGTT	CAGAGGGAGTAGTGTTTATGG
**BoGMS539**	ACGACACCGTATTGATTCTC	ATCCACTCACCTCCCAGT
**BoGMS545**	CCTCTGTTTCTTTGCTCTTTG	GATTCATTGTGTGTGTGATGT
**BoGMS560**	ATGAAGAAGTGTTTGGTGAG	AAGATTGATGGAAGCAAGAA
**BoGMS561**	CAAAGACTAAACCAGACCAAG	ATAGGCAAGGTGAGAAGAAAG
**BoGMS576**	ACCTGGAGTTGAGACGGG	CAGTGTTGAGTGTTCTTCTATT
**BoGMS586**	GCTGTATCATCTTCTTGCTCT	TGTTTCTTTGTAGTTTGGTGG
**BoGMS622**	ATCTGCCTTCACCGACTT	TCTGGTTCAGTTCGTTTATTT
**BoGMS680**	CTCTGCGTAAGAACATCACA	CGCTAAACGACCTCTACCA
**BoGMS693**	CATCTCCACATCACTACTCCA	ATTACCCAATCCCAACAAA
**BoGMS788**	CGCATTACTATCTCCATTGTC	GGCTGTCACTCTCTTCGG
**BoGMS795**	AACTACCAAGTCGTGTGAGAA	CAAAGTAAGCAAGTGGAGAAA
**BoGMS819**	AGGGAGATGGACACATTTAG	GAGAGAGGGCAAAGAAGATAG
**BoGMS829**	CACACTCATCTCTGTTTCAGTT	TTCACTTCCAGCATTACGA
**BoGMS876**	TGATGATAACTTGTAAATCTGCT	TTCTTTGGTGGTCCTGGT
**BoGMS1032**	GCCGTCGTCATCTTCTAC	ATTGAGTATCCCTTCTCTGCT
**BoGMS1059**	TAAGCACGCAACTGTGGA	GGGTTATTTGAACGGAGTATG
**BoGMS1072**	CATTGTCTCGTCTGCTCAA	AGCCCATTAGTTCTCTCTCAC
**BoGMS1114**	CACCACTAACCTATGAACCTG	TGTCTCTTCTCCACTTTGTTG
**BoGMS1153**	ATTAGTGCGTATCATTGTGG	GGGTTCTTGCTCTTGGATT
**BoGMS1166**	TAATGGAAACGCACCAAG	AAACAATCTTAGCAGAGGAGG
**BoGMS1186**	GACTGGAACGACAACGACT	GCGGAGGTAGATTAGGGA
**BoGMS1199**	CCTTACTTCTCTGTGGTTGG	CGGGAACTTTGTGGACTT
**BoGMS1200**	CCGTCGTCTCTCCTAAATC	TTATTGGGTCGGTTATTCACT
**BoGMS1201**	GGTAACACCAATCCACTTTCT	CACTTCAAACTTCTCATCTGG
**BoGMS1203**	GTTATCCACGCTTTGGTG	GCAAGACACTTATTATTTGAGAC

**BoGMS1230**	AAGTTGTCCCTGTTCGTG	CATTCTTGGTCGGAGTTAGTT
**BoGMS1246**	CACTACCCGTTCGTTCAG	ACATTCAGCCACTCTGCTT
**BoGMS1295**	CTCCTTCTTCATTCTTCGG	AATCTCCCTATTGCTTGTTC
**BoGMS1303**	ACACGAGCCATCACCTACT	CTCTCCTCCTCTCTTAGCATT
**BoGMS1307**	TGGCGATAAAGAGGAGAAC	CGAAGAGAGAGAAACAAAGAA
**BoGMS1308**	ACGATTTGGTATCTTCTTTCC	GTTCCGACTAACACTCTCTCA
**BoGMS1320**	TCTCCAAATCCTTCTTCTCAC	CCACCTTCTCCACTAATAACC
**BoGMS1373**	CCACCTTCTCCACTAATAACC	TCTCCAAATCCTTCTTCTCAC
**BoGMS1375**	ACACACCATCTCTCTCATCTC	GGAACCTCTCTGCTCTCAA
**BoGMS1382**	CTTCCTGCGTCACTTTACC	AGGCTGTCTCCTCACCCA
**BoGMS1399**	ATGTTCCCATTTCAGAGTCA	TTATCCCAATCCTCATCATC
**BoGMS1425**	GGCGGCAAGACTCAATAA	ACTTTGGTTAGAGAAGAAATGA
**BoGMS1464**	CTGATGAACGGAGACACAG	AAGCAAAGCAGAGCATAAAC
**BoGMS1484**	ACCTGTTGTTCACTTCATCAC	TTGAGTCGTCACTGTTTCATT
**BoGMS1558**	CTACGACCTACAAGAATGCC	CATCAGTAAAGCAGAGAACAAA
**BoGMS1561**	AGATAACGGATGTGAGGAGA	CTTGAGAAGATTGGCTTGTT
**BoGMS1575**	CCAGCAGAGAATGTGCCT	TTGAAATAGACTTTGGTTTGG
**BrGMS5**	ATTTCGGATCATAGCTGTTCCA	TCAGAGAAACACAACACCAAGC
**BrGMS38**	AGGTTTGGGAGATGCAGAGTT	GCCACTGAGACTTCCTCCTCT
**BrGMS124**	CGAGGAGAACAGAAGGGATAGA	CGCTGGGTACAGAAACCTAAAC
**BrGMS216**	ACACGGGCTACAAAACAAGAGT	ATGCTTCTGATTTAACCTGGGA
**BrGMS217**	CATTCCGCCTAAGCTCATTAAC	CAATTTCACCTGCACCTAGTCA
**BrGMS231**	TGAACCACCAACGTGTTCTTAC	TTTCCCTAGATCCGCTGTCTTA
**BrGMS268**	TCACCATGTGTAAAAGACTCGG	TTGGCTTATCTTCTTGGGATTG
**BrGMS316**	TGGGACCATAAATTGGATAAGG	GTGTGGTTTGTGAGAGATGGAA
**BrGMS351**	TGCATCCAGACAGTTACCAAAT	TTAACGCTCATTTCACTTCCTC
**BrGMS363**	AAGAAGTCTCACAAACGAAGCC	AAGATCAAAACCTCATTGCCC
**BrGMS375**	TATTGGAGGCATCACATCTCAA	AGCTGCAACACAGAGAAAGACA
**BrGMS387**	GCGTAATCTCAAGCGAAACAAT	GAAGCCGACAATTCCCCT
**BrGMS394**	ATCTACTTTCCCTTCACCACCA	GAAATGATCTTGCTGACCACAC

**BrGMS406**	TCGATAGACTGAGCAACGGTAA	GGATACACATTTTGGGAGAGGA
**BrGMS426**	CCGTTGAAGTTGTAGAGACGAG	TGCTTGAAATACATGATACCCC
**BrGMS509**	TGGAGACGTGTGTTCATTTCAT	GTTCACCCCTGACATTAGTTGG
**BrGMS556**	GGTTGGTTCCTCCTCTTCTTCT	TTTAACTTCAGCATCTGGGGAT
**BrGMS679**	ACTGGCGAGAAAAGAGATGAAG	GAGAATGGGCTTTGTTAGTTGG
**BrGMS725**	GATCACATATTCGTCGTGTTGC	AGGAAGTGACGTGTTTCCGTAT
**BrGMS726**	GATCATTGCTTATCCTGCGAAT	ACGGCATGGAAACGTCTATAA
**Ap1c 5pr**	GCGTAGGAGGTGGTAAAGATAGC	ACACCCACATTGCATTTTACCC
**BN12A**	GCCGTTCTAGGGTTTGTGGGA	GAGGAAGTGAGAGCGGGAAATCA
**BN25C2**	AAACCTCCTCAAAAACCCCTAAACG	TCCCCTCTTTCCTCTCTCTCTAGGC
**BN38A**	TGGTAACTGGTAACCGACGAAAATC	ACGCTGTCTTCAGGTCCCACTC
**BN9A**	GAGCCATCCCTAGCAAACAAG	CGTGGAAGCAAGTGAGATGAT
**BRAS005**	CGTTCGTTGTTGCCCTCTACT	CTCTACACGACGGCGATAAAA
**BRAS019**	CTCAAGACAAACGACCAGTAA	GAGAAGAAATCGCCAAGA
**BRAS021**	ACCGTTGAGATCAATCCCTAT	CATCTTCCTTAATCGAAACCC
**BRAS051**	GAATAGCCTCGCAGAAGTAGC	CGACGGCGATAAAACGAA
**BRAS063**	GACGCTCATTTCACTTC	TCCTAACTAACATCATTTTGC
**BRAS072**	GCCATCTACACATTTATCCC	CACTAACCTTCTTGCTACCGT
**BRAS074**	AATTTAAACCTCATTTTCTTC	ACCTCCATTGTGTCTGAT
**BRAS083**	GATGTTGTTGGGGAGAATG	AAAAAGTAGGCAAGTTCAAGC
**BRAS087**	GCAGAATAGCCTCGCA	GGAGGAAAAGAACGTGG
**BRAS120**	AAAAATAAATACAGCGAACC	ACCTTTAGCAGCTAATCATC
**BRMS-006**	TGGTGGCTTGAGATTAGTTC	ACTCGAAGCCTAATGAAAAG
**BRMS-007**	AAATTGTTTCTCTTCCCCAT	GTGTTAGGGAGCTGGAGAAT
**BRMS-008**	AGGACACCAGGCACCATATA	CATTGTTGTCTTGGGAGAGC
**BRMS-026**	CCTATCCTCGGACTAATCAGAA	GTGCTTGATGAGTTTCACATTG
**BRMS-049**	GATCTTCTCTCCAAAACTCTCT	AAAGTCCAAGCTAAATTACAAA
**BRMS97**	AAGAGCAAGAGCCAAGACAGCCTAC	TGGTGATGGAGAAGTACATGGACGT
**CALSSR**	GTTAAGTGTGGCGTTAGAGG	CCTTGGTACATGCCACTGAA
**CB10022**	AACAACCAAACATAGTCCC	GTTGACTTTGACCTTGACTT

**CB10028**	CTGCACATTTGAAATTGGTC	AAATCAACGCTTACCCACT
**CB10034**	TACGGGATAGGCGACGAA	CCTAAGCGAGACGTATTG
**CB10057**	CTAGGCTAAGGAAGATTGTCA	TAGTTTCTTCCTCCTGCTATC
**CB10196**	TTGTAGGCAATGATGAGGA	GAGAGAAGGGCTCCTTTG
**CB10217**	CCCCATATCATCCCTACC	GCTGAACAACCCACAAAG
**CB10258**	ATGATGCCTAGCATGTCC	AAGCTAAAGCGAAAGAAGC
**CB10297**	CTCATTCCACTGCCAAAC	ACGAGTCACCATGTCAGG
**CB10299**	TACAGGTTCCTTGCGATG	ATGGACGAGACAACATGG
**CB10330**	AGGCGAGTTTACGAGGAT	ACCTGCACCAGTCATTTG
**CB10347**	ATCTGAACACTTTCGGCA	GGAAGCACCATGTCAGC
**CB10413**	CTTAGCACAACCACTCGG	GGTGATGAAGATGACGATG
**CB10439**	ACCTCGAAGGGTATCTGC	CGTGCAATTTCAACAACA
**FITO007.2**	ggtcggcacgagagctttaag	cctaaaatttaatcgcacgccg
**FITO018**	tgccaagctttgagtagcaa	tgccttcttccatgctttct
**FITO035**	aaagtcgtgggaagtatcgt	aggtgtaaggatggtggtagt
**FITO095**	agatttcatccacagcctc	tttgattcttgcgttctctc
**FITO114**	gcgatacaaagaaatccaaa	caacaaataaccaaacattga
**FITO131**	ctacaacgggcagggtga	aaatcccatctactggtttga
**FITO135**	tgtaagacaacaccattcctt	cacagtcacatccaccataac
**FITO136**	cctcctcctcagacttacact	tcacatccaccataaccttt
**J0609**	GTTGGTTAAAATCGTGTATGC	CCTACAAAAAGCAATAACGTG
**MR52a**	TCGACATGGATTCTACCAAA	GAACTTGCAAGCTGCAATTA
**Na10-A09**	TCTTGAGCAAAGAAACTTGG	CAAACTGAGCCATACACAAAGG
**Na10-C01**	TTTTGTCCCACTGGGTTTTC	GGAAACTAGGGTTTTCCCTTC
**Na10-E02**	TCGCGCATGTAATCAAAATC	TGTGACGCATCCGATCATAC
**Na10-G10**	TGGAAACATTGGTGTTAAGGC	CATAGATTCCATCTCAAATCCG
**Na12-A01**	GCATGCTCTTGATGAACGAA	GCTTCAACCTCTCAATCGCT
**Na12-B02**	CGCGTTGTAACTCCAAGACC	TTTTCTAAATCATCCACTGTATCC
**Na12-C01**	CCAGGTTACTGTTAAAGAATAAGAGAG	ATCGTCTGCGAGTCTCCTTG
**Na12-C06**	AACGGATGAAGAACACATTGC	TAGGGCCTGTTATTCGATGG

**Na12-C08**	GCAAACGATTTGTTTACCCG	CGTGTAGGGTGATCTAGATGGG
**Na12-D08**	ACGACGATTCAACTCATCTTC	TTAACCAACTTCGCTTTTTG
**Na12-D11**	AATTGCATTTTGTATCCAGCG	ACATCCCTAGGATCAATGCC
**Na12-E01**	ATTCCATGACTCCATTGTC	AAATCCCTTGTCTCTGTCG
**Na12-E02**	TTGAAGTAGTTGGAGTAATTGGAGG	CAGCAGCCACAACCTTACG
**Na12-E05**	CGTATGTTTGTTCCACCTGC	ACTAGCAACCACAACGGACC
**Na12-E09**	CATGAGAACAAGATGGGTTCG	CTGAAACTTGAGCAAAGCCC
**Na12-F03**	GGCGACATAGATTTGAACCG	TCCACTTTCTCTCTCTTCCCC
**Na12-G05**	CCGATCATACCTTTTACTCTAGCC	GATGTTCCTCTCGGTGATGC
**Na12-H02**	CTATGGTTCATCTTTCGCCG	GCTGCACATCCATCTCTCG
**Na12-H09**	AGGCGTCTATCTCGAAATGC	CGTTTTTCAGAATCTCGTTGC
**Na14-B03**	GATGGTCGCCGATTCAATGA	CCCATCAGCACTAGAAACCA
**Na14-E02**	ACTGGCTACATGAGTTTCAGTG	GAGGGAAGACAACTGGTCTCA
**NIAB_SSR106**	GTCTCAAGCCAACATCCATC	AACGGAACCATAAGGAGACC
**Ol09-A06**	TGTGTGAAAGCTTGAAACAG	TAGGATTTTTTTGTTCACCG
**Ol10-B01**	CCTCTTCAGTCGAGGTCTGG	AATTTGGAAACAGAGTCGCC
**Ol10-D01**	TCTCTGCCAAAAGCAAATAGC	CTTGGCTCTCTCTCACCACC
**Ol10-D03**	GCCAAAGACCTCAAAGATGG	AAGCCACGTGAAGAAAGTCC
**Ol10-E05**	GCCAGAAACAGGAGAAATGG	GAAGCCGAAGAAAATAAGCG
**Ol10-F09**	AGAGAGCGAGATTGATTGGC	AAACGACCACGAGTGATTCC
**Ol11-B05**	TCGCGACGTTGTTTTGTTC	ACCATCTTCCTCGACCCTG
**Ol11-H09**	CCCTTTTCCCCTTCTATTGG	GTGCGACTTGGAATTTCTCC
**Ol12-E03**	CTTGAAGAGCTTCCGACACC	GACGGCTAACAGTGGTGGAC
**Ol13-G05**	GTGTGCAGGAAACGATGTTC	GGGAGTTTGAAGAGAAAGCG
**pMR181**	AGATTTGCATGTGGTTTGAC	ATTGCTTANTGATGTTGGGAA
**Ra2-G08**	ATGTCCGGATAACCGAATCC	GAAGCTTTTCAATTTTTAAGTTCTCTC
**Ra3-D04**	AAAAGGACCTACCAATTTCGTG	CGACCCAAACTGAGCCATAC
**sN0706**	TCCGACGGTCAAGATTAAGG	GGCTGTGGTGGATCTAGGAA
**sN13034**	AGCATGTGCAGAGTGCAGAC	AAAGGAAGGCCGATGAGATT
**sN1988**	TTCCCTCCCTCCCTCTTAAA	CGTTGGATGCAGAGCTGTTA

**sN2025**	AAGGAGCAAACACAAGCGAT	GCACAAGCACTCTACGGTGA
**sN2032**	CACCATCACCACCTTCACAA	TGGTTCACTCATGTCTCCGA
**sN2837**	CACACCCTTACCACGTTCCT	GCAACAAAGCATACTTCGCA
**sN3523R**	CGTTGGCAAAAAGCCTACTC	CTCAGGGACGTCGTAAGAGC
**sN3761**	CGACAGAGGGTTCAAATGGT	CGGTGTGTAGGTCTGCTCAA
**sN9425**	CGCCGACTTTAAAGAGATGG	TCCTTGGGTTGGTCACTGAT
**sNRA56**	AATCTTCGCATACCCATTCG	GTTTGACGCGGATAACCATC
**sNRA59**	CAGATTCGGATTTGGGAAGA	GGCGGAAGAATCAAAGGAGT
**sNRH63**	GAATCAGTCACCAGGGGAGA	CCATCGCGGTAATTAAACCT
**sORH13**	CCTGATGTTTTGGTTGTGTCA	TCACTGTGTTTACTTGCGCC
**sR0282R**	AGGAAGCCCAACAGGACTTT	AATTCGATTCTCCATCGTGC
**sR10417**	CGGAGAAGAAACGAGCATTC	TAGGGTTTCTGACCCGATTG
**sR12095**	GCTGCGAGGAAATCAGAGTC	TACACACTTGATCGCGCTTC
**sR12137I**	TCGGAATCAGATTAAACCCG	CATTATTGAAAAACACACGCC
**sR12156**	TTCTTGGTGTGTCCTGCAAA	GAGCTATGGCAATCCTCCTG
**sR12777**	CAAGCAGTTTAAGGAACCGC	ATAATTGCATTTTGCTCCGC
**sR3688**	GGAGTCCACTTCATGGAGGA	CTCTTGCTCGTAGGTTTCCG
**sR4047**	AGAGAGGGTGTTGTTCCCAG	ATTCTTTGAATGGCCAGCAC
**sR6083**	CTTGTTCTTGCGTAGCCACA	ACTTTGCTCTGGCTCAGCTC
**sR6293**	CCAAACGCTTTTCTTTCTGC	CCAATGACGCTCCAAGATTT
**sR7223**	AGGACCCGACTTTCCTTGTT	ACCAAACTCGGCGTACAAAT
**sR9251J**	TCTCCAGTACGGGGAAGAGA	CAAAGAAGCCATGTGTGGAA
**sR9411**	GAGCATAGGGCAAAAACTGC	CGATGTCGCTCTCAATCTCA
**sR9447**	AAATTCGAAAATGCAAACGG	CCAATCTTGGAACAATAGAAGATG
**sR9477**	CAGCTGGTTATCCTCGGTTT	CCTCAGGTGGACAGAGAAGC
**sS1867A**	TCACCAGGGCTGATCTATGA	GGGTGATCTAGATGGGTGAGTG
**sS2131**	TTGCTTCCAGAGATCTGGTTC	TGCGACTACTAAATTGTCGTGTT
**sS2277**	GATCTGCGGTAGGAATCGAA	CGTGCTACATAATAGGGAAAAACC
**sS2331B**	TGTCCTGTTTTCTGTGCTGG	GCCAACGCTAGTTTTGCTTC
